# The global, regional, and national burden of urolithiasis in 204 countries and territories, 2000–2021: a systematic analysis for the Global Burden of Disease Study 2021

**DOI:** 10.1016/j.eclinm.2024.102924

**Published:** 2024-11-21

**Authors:** GBD 2021 Urolithiasis Collaborators, Atalel Fentahun Awedew, Hannah Han, Bétyna N. Berice, Maxwell Dodge, Rachel D. Schneider, Mohsen Abbasi-Kangevari, Ziyad Al-Aly, Omar Almidani, Saba Alvand, Jalal Arabloo, Aleksandr Y. Aravkin, Tegegn Mulatu Ayana, Nikha Bhardwaj, Pankaj Bhardwaj, Sonu Bhaskar, Boris Bikbov, Florentino Luciano Caetano dos Santos, Jaykaran Charan, Natalia Cruz-Martins, Omid Dadras, Xiaochen Dai, Lankamo Ena Digesa, Muhammed Elhadi, Mohamed A. Elmonem, Christopher Imokhuede Esezobor, Ali Fatehizadeh, Teferi Gebru Gebremeskel, Motuma Erena Getachew, Seyyed-Hadi Ghamari, Simon I. Hay, Irena M. Ilic, Milena D. Ilic, Umesh Jayarajah, Seyed Behzad Jazayeri, Min Seo Kim, Sang-Woong Lee, Shaun Wen Huey Lee, Stephen S. Lim, Mansour Adam Mahmoud, Ahmad Azam Malik, Alexios-Fotios A. Mentis, Tomislav Mestrovic, Irmina Maria Michalek, Gedefaye Nibret Mihrtie, Erkin M. Mirrakhimov, Ali H. Mokdad, Mohammad Ali Moni, Maryam Moradi, Christopher J.L. Murray, Alberto Ortiz, Shrikant Pawar, Norberto Perico, Mohammad-Mahdi Rashidi, Reza Rawassizadeh, Giuseppe Remuzzi, Austin E. Schumacher, Jasvinder A. Singh, Valentin Yurievich Skryabin, Anna Aleksandrovna Skryabina, Ker-Kan Tan, Musliu Adetola Tolani, Sahel Valadan Tahbaz, Rohollah Valizadeh, Bay Vo, Asrat Arja Wolde, Seyed Hossein Yahyazadeh Jabbari, Fereshteh Yazdanpanah, Arzu Yiğit, Vahit Yiğit, Mazyar Zahir, Michael Zastrozhin, Zhi-Jiang Zhang, Alimuddin Zumla, Awoke Misganaw, M. Ashworth Dirac

**Keywords:** Urolithiasis, Global burden of disease, Nephrolithiasis, Incidence, Disability adjusted life loss

## Abstract

**Background:**

Urolithiasis is a common urological problem that is associated with high morbidity. A comprehensive assessment of the non-fatal and fatal health trends of urolithiasis by age, sex, and geography over time is necessary to inform policy to control this surgically managed non-communicable disease.

**Methods:**

This study was conducted using the standard GBD methodology and analytic tools. Cause-specific mortality rate (CSMR) was estimated using vital registration and verbal autopsy data and the Cause of Death Ensemble model (CODEm) modelling tool. CSMR estimates and incidence data from medical insurance claims and hospital discharges were analysed using a Bayesian meta-regression modelling tool, DisMod-MR 2.1, to estimate age-, sex-, and location-specific incidence of urolithiasis between 2000 and 2021. Disability-adjusted life-years (DALYs) were the sum of years of life lost (YLL) and years lived with disability (YLDs). YLLs due to urolithiasis were calculated by multiplying the estimated number of deaths by the standard life expectancy at the age of death. YLDs were estimated by multiplying the disability weight by the symptomatic proportion of urolithiasis cases. The Global Burden of Diseases study used de-identified data, approved by the University of Washington IRB (Study Number 9060).

**Findings:**

There were 106 million (95% UI 88.3–129.0) incident cases of urolithiasis in 2021, of which 67% were in men (71.1 million [59.4–86.2)]). The global number of incident cases, deaths, and DALYs increased by 26.7% (23.8–29.8), 60.3% (41.5–84.7), and 34.5% (24.6–47.3), respectively, between 2000 and 2021. The global age-standardised incidence rate of urolithiasis experienced a significant decrease of 17.5% (14.7–20.0), while the age-standardised DALYs rate saw a reduction of 15.1% (6.8–21.3). Twelve GBD regions showed declining trends in the age-standardised incidence rate of urolithiasis between 2000 and 2021, and the remaining nine GBD regions had an increasing trend of age-standardised rates of urolithiasis. A significant increase in the age-standardised incidence rate of urolithiasis was observed in Central America, Tropical Latin America, and the Caribbean regions, whereas notable decline was observed in east Asia, eastern Europe, central Europe, and high-income North America. It was observed that the global age-standardised death rate was less than 0.5 per 100,000 across all GBD regions and less than 1 per 100,000 across all SDI quintiles, with fairly stable global age-standardised death rates of urolithiasis between 2000 and 2021. The age-standardised incidence rate of urolithiasis was 837 (688–1034) in low SDI regions and 1443 (12,108–1734) in high-middle SDI regions. Furthermore, the age-standardised DALY rate showed a decreasing trend across all SDI quintiles over the same period: high-middle SDI (−28.9% [–34.4 to −23.0]), middle SDI (−22.6% [–30.5 to −10.9]), and low-middle SDI (−2.9% [–15.8 to 12.9]).

**Interpretation:**

Global urolithiasis incidence and DALY rates have decreased, while the death rate has stabilised worldwide, showing significant variability among regions, SDI levels, and countries. This could be due to effective preventive measures c on urolithiasis risk factors, effective public health education, lifestyle changes, and early interventions and improved health care access at the global level. This analysis offers relevant insights into global, regional, and country-specific urolithiasis trends.

**Funding:**

10.13039/100000865Bill & Melinda Gates Foundation.

**Research in context**

**Evidence before this study**

Our research reviewed studies on urolithiasis incidence, mortality, and DALYs through a PubMed search of English-language papers from 2000 to 2024, including terms like “kidney stone” and “global burden of disease.” While some papers indicate rising cases and deaths attributed to urolithiasis, all identified studies pre-date the COVID-19 pandemic. This study highlights the urgent need for updated global burden data to effectively inform current health policies.

**Added value of this study**

This study offers a comprehensive analysis of the global urolithiasis burden using the latest GBD 2021 data, merging incidence, mortality, and DALYs for a complete picture over the past two decades. Stratified by sex, age, and SDI at various levels, it reveals how urolithiasis impacts different populations. Additionally, it provides valuable insights for evidence-based healthcare strategies and fills a knowledge gap with a thorough evaluation of sex-specific trends from 2000 to 2021.

**Implications of all the available evidence**

Urolithiasis is a costly condition, for which prevention and care have been effective in many parts of the world, particularly for men, but additional effort must be made to improve prevention and care for women and for individuals living in countries with low SDI. These findings underscore the crucial need for sustained prioritisation of policies and interventions aimed at preventing and treating urolithiasis. Additionally, continued efforts to strengthen health systems are essential for ensuring access to timely and effective care for individuals affected by this condition.

## Introduction

Urolithiasis is a formation of stone in the kidneys, ureter, bladder, or urethra.^1^, ^2^, ^3^ It has contributed enormous burden of morbidity, disability, and medical cost worldwide.^4^^,^^5^ Urolithiasis can have serious health consequences, such as severe colicky pain, obstructive uropathy, infection, hypertension, and renal failure.^6^ Urolithiasis is associated with high medical expenditure.^2^^,^^7^^,^^8^ The estimated annual cost of urolithiasis in USA will be $4.57 billion in 2030.^7^ Prior reviews of urolithiasis epidemiology have reported increasing incidence globally, especially in high-income countries.^4^^,^^9^^,^^10^ Proposed explanations for this increase include changes in distribution of predisposing factors^8^^,^^9^ and improved diagnostic capabilities.^10^ Various risk factors contribute to the pathogenesis of urolithiasis including age, male sex,^11^^,^^12^ geographical factors such as climate change and seasonal variation,^13^^,^^14^ diet,^1^^,^^14^^,^^15^ occupation, genetic inheritance, metabolic syndrome, obesity,^15^ diabetes, and hypertension.^1^ These risk factors and associated diseases have a direct or indirect role in the development of hypercalciuria, hypocitraturia, low pH, hyperuricosuria, hyperoxaluria, cystinuria, and infection with urease-producing organisms, increasing the risk of acute urolithiasis.^1^^,^^4^^,^^14^^,^^16^ The chemical composition of uroliths is also heterogeneous,^17^ ranging from more common calcium-containing stones (calcium oxalate, hydroxyapatite, or brushite) to less common uric acid, magnesium phosphate, and cysteine stones,^9^^,^^14^^,^^16^, ^17^, ^18^, ^19^ making overall trends difficult to predict from risk factor trends alone. Previous epidemiological evidence suggested that the incidence of urolithiasis has been increasing worldwide, with considerable variation by race, gender, age, geography, and regions.^20^ Many studies have suggested that rising incidence in recent decades is driven by a relatively greater rise in females, closing an historic sex gap.^11^^,^^21^, ^22^, ^23^, ^24^ However, prevalence, incidence, mortality, and disability of urolithiasis have been measured with inconsistent methods. The Global Burden of Diseases, Injuries, and Risk Factors Study (GBD) are the only global comprehensive source of evidence on burden and temporal trends of urolithiasis that standardises epidemiological measurements across settings. This report expands upon prior GBD 2019-based publications on burden and temporal trend of urolithiasis^25^, ^26^, ^27^ by updating to GBD 2021 results and more deeply exploring temporal trends by sex. Furthermore, this is the first report on urolithiasis burden provided by the GBD Collaborative, which provides more rigorous discussion of input data, methods, limitations, and priorities for future research.

## Methods

### Overview

The GBD project is a comprehensive study that aims to quantify the burden of mortality and morbidity due to 369 diseases and injuries. The analysis presented here followed the standard GBD methodologies, which have been described in detail elsewhere.^28^ This study complies with the Guidelines for Accurate and Transparent Health Estimates Reporting (GATHER).^29^^,^^30^

### Case definition

In our study, we used the following International Classification of Disease version 10 (ICD-10)^31^ codes to define urolithiasis: N20 (“calculus of kidney and ureter”), N20.0 (“calculus of kidney”), N20.1 (“calculus of ureter”), N20.2 (“calculus of kidney with calculus of ureter”), N20.9 (“urinary calculus, unspecified”), N21 (“calculus of lower urinary tract”), N21.0 (“calculus in bladder”), N21.1 (“calculus in urethra”), N21.8 (“other lower urinary tract calculus”), N21.9 (“calculus of lower urinary tract, unspecified”), N22 (“calculus of urinary tract in diseases classified elsewhere”), N22.0, N22.8 (“calculus of urinary tract in other diseases classified elsewhere”), and N23 (“unspecified renal colic”).

### Statistics

#### Fatal estimation

Mortality data comprise over 2500 country-years of data from vital registration systems, verbal autopsy studies, censuses, and surveys between 1980 and 2018. Processing of mortality data has been reported previously.^28^^,^^32^ In brief, cause of death (COD) data were extracted as cause fractions, or cause-specific deaths divided by all-cause deaths in a given source, and combined with GBD estimates of all-cause mortality rates and population to obtain rates.^33^ COD data from various data sources were pooled and standardised to account for different coding practices, age aggregations, misclassification of maternal and HIV/AIDS deaths, completeness, and representativeness. Deaths that were mapped to diseases that cannot be underlying causes of death were proportionately reassigned to valid GBD-defined causes using several methods, including an algorithm derived from multiple causes of death data. To estimate mortality from acute urolithiasis by age, sex, year, and location, we used the GBD Cause of Death Ensemble modelling approach (CODEm).^28^^,^^34^ CODEm is an instrument that tests numerous linear mixed-effects models and spatiotemporal Gaussian process regression models with different sets of predictive covariates.^28^^,^^35^ The individual models with the best predictive validity are weighted and combined to produce the ensemble model that has the greatest out-of-sample predictive validity. The list of predictive covariates tested in CODEm for urolithiasis can be found in the Appendix. The results from the CODEm models were adjusted using the CoDCorrect analysis to ensure internal consistency of cause-specific mortality and all-cause mortality estimates of all diseases within the GBD cause hierarchy.^28^^,^^32^ Years of life lost (YLL) due to acute urolithiasis were calculated by multiplying the estimated number of deaths by the standard life expectancy at the age of death.

### Non-fatal estimation

Data inputs to estimate the non-fatal burden of acute urolithiasis comprised hospital inpatient admission data from 47 locations, health insurance claims data from the USA, Taiwan (province of China), and Poland, and cause-specific mortality rate outputs from the fatal burden estimation process described above. Data processing of incidence inputs has been described in detail elsewhere.^28^ Briefly, in claims data, an enrollee was extracted as an incident case if they had at least one inpatient or outpatient medical encounter with any of the defining codes for urolithiasis in either primary or secondary diagnostic position. Multiple medical encounters of urolithiasis within one year, regardless of setting, were assumed to be of a single episode; the date of the first medical encounter was used as the incident date. Enrollees for that year were used as the denominator. For hospital discharge data, urolithiasis discharges were extracted if a defining code for urolithiasis was the primary discharge diagnosis. The inpatient urolithiasis discharge counts were then adjusted to reflect readmission, secondary diagnoses, and cases not requiring inpatient admission by applying the ratio of inpatient discharges with urolithiasis as primary diagnosis to total incident cases identified in any healthcare setting as observed in insurance claims data.^28^ Where hospital discharge data sets were considered to cover an entire population, these adjusted incident cases were combined with population size estimated by GBD for the same year, age, sex and location to obtain an incidence measurement.^33^ Where hospital discharge data sets were considered to cover only a subset of the population, we calculated cause fractions as the adjusted incident cases of urolithiasis divided by the total discharges in that data set for that year, age-group, and sex combination, and we then multiplied these cause fractions by the hospital admission rate per capita and total population size for the corresponding location, age, sex, and year combination.

Taiwan and Poland claims data and adjusted inpatient data were assumed population-representative in our model. Using a Bayesian regularised, trimmed meta-regression (MR-BRT) analysis, the USA claims data were adjusted toward the USA inpatient data to account for selection bias associated with commercial insurance status. Detailed information about the MR-BRT analysis has been reported previously.^28^ Given the heterogeneity of input data, the data series with an age-standardised incidence greater than two median absolute deviations from the median of the age-standardised incidence of all data were marked as outliers and excluded from the analysis.

In addition to cause-specific mortality rate outputs from the fatal burden estimation, we modelled excess mortality rate (EMR) of acute urolithiasis by age and sex as a function of the Healthcare Access and Quality (HAQ) Index^36^ to impose an expected pattern of EMR, which illustrates a negative association between EMR and the quality of health systems and health care access.

Together, incidence data, modelled EMR estimates, and cause-specific mortality rates (CSMR) from fatal estimation were used as inputs to DisMod-MR version 2.1 to model the incidence of acute urolithiasis for all years, age groups, sexes, and locations. DisMod is a Bayesian mixed-effects meta-regression tool that synthesises various input data to produce internally consistent prevalence, incidence, remission, excess mortality rate, and cause-specific mortality rate estimates.^28^^,^^37^ The DisMod outputs were combined with disability weights to produce YLDs. Specifically, incidence estimates were multiplied by the disease duration (two weeks) and severity state proportions estimated from the Medical Expenditure Panel Survey^38^ to generate asymptomatic, mild, moderate, and severe categories. Each disease severity was assigned a disability weight, which represents the severity of health loss due to a disease ranging from 0 (“perfect health”) to 1 (“death”).^39^, ^40^, ^41^ Disability weights associated with mild, moderate, and severe symptomatic cases of acute urolithiasis were 0.011 (95% uncertainty interval^40^ 0.005–0.021), 0.114 (0.078–0.159), and 0.324 (0.220–0.442), respectively. DALYs were calculated by adding YLLs and YLDs together.

### Uncertainty

The 95% uncertainty intervals (UI) of the final burden estimates were calculated by producing draws of the posterior distribution at every data processing and modelling step, carrying out draw-level calculations for any scaling, and employing the 2.5th percentile and 97.5th percentile of the resulting distribution. For the fatal burden estimates, estimation was carried out using 1000 draws, and sources of uncertainty reflected in final intervals include sampling error and the uncertainty of garbage code redistribution in the input data, uncertainty from regression parameters and the heterogeneity of submodels in CODEm, and the all-cause mortality envelope in CoDCorrect. Nonfatal estimation was carried out using 500 draws, and final uncertainty intervals take into account sampling error in incidence data; uncertainty in model-fitting and between-study heterogeneity when estimating correction factors for incidence data, EMR inputs, and CSMR inputs; uncertainty in the regression parameters in DisMod; and uncertainty in disability weight and severity distribution data and modelling. Final uncertainty estimates do not reflect uncertainty in data and estimation of the values of predictive covariates used in CODEm and DisMod; these are provided to the model as mean estimated values for each year-age-sex-location combination, because of the computational capacity it would take to bootstrap each covariate. Synthesising the fatal and nonfatal estimates, distributions of the age-sex-year-location-specific DALY estimates were calculated by summing draws of YLLs and YLDs, assuming no correlation between the uncertainty in YLLs and YLDs.

### Socio-demographic index

The Socio-demographic Index (SDI) is a composite indicator for development status of each country. It is derived from three GBD indicators: total fertility rate in women under 25 years of age, mean educational attainment for those 15 years or older, and lag-distributed income per capita.^42^

### Ethical approval

The Global Burden of Diseases study used de-identified data, approved by the University of Washington IRB (Study Number 9060) (https://collab2021.healthdata.org/gbd-results/).

### Role of the funding source

The funder of the study had no role in study design, data collection, analysis, interpretation, or the writing of the report. The corresponding author had full access to all of the data and the final responsibility to submit for publication.

## Results

### Global

There were 106 million (95% UI 88.3–129.0) incident cases of urolithiasis in 2021, of which over 67% were in men (71.1 million ([59.4–86.2)]).

**Fig. 1:**
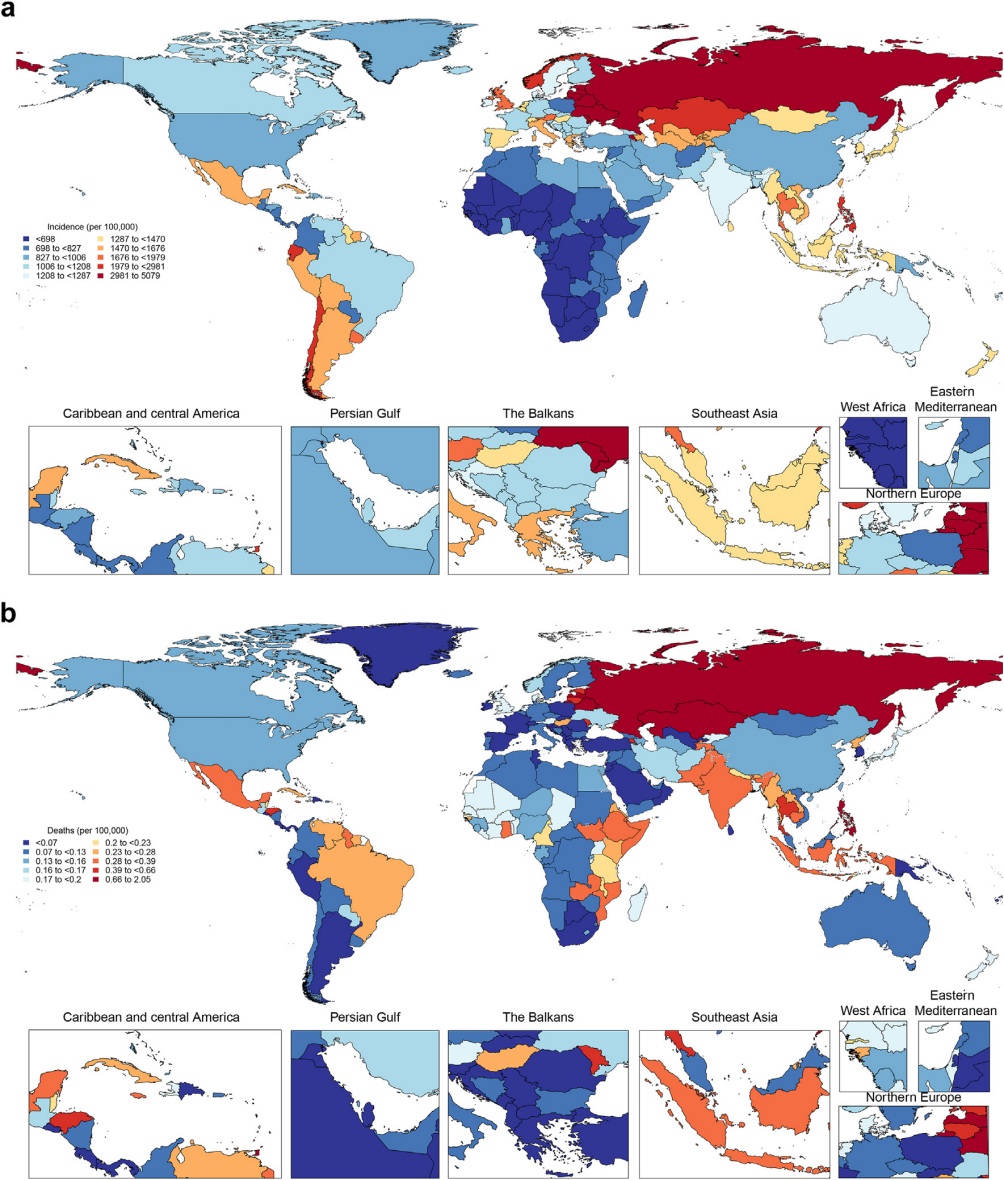

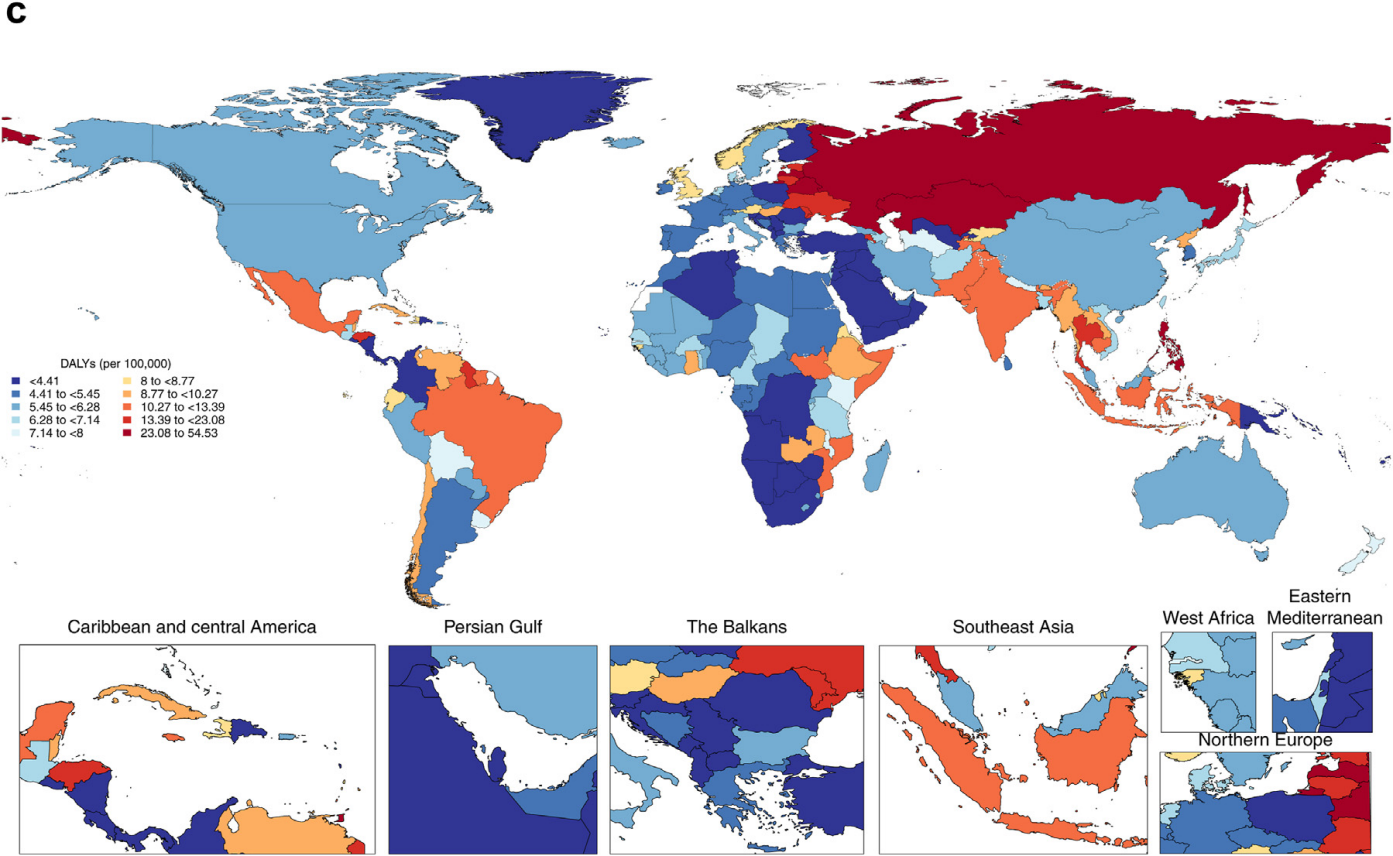
Age-standardised rates of urolithiasis in 2021 (a = age-standardised incidence rate, b = age-standardised death rate, c = age-standardised DALYs rate).

The global number of incident cases increased by 26.7% (95% UI 23.8–29.8) between 2000 and 2021. However, when controlled for population growth and ageing, the global age-standardised incidence decreased from 1450 per 100,000 (1200–1790) to 1240 per 100,000 (1030–1510) during the study period, representing a 17.5% (14.7–20.0) reduction (Fig. 1).

In 2021, the global age-standardised incidence rate of urolithiasis was 815 per 100,000 (95% UI 680–995) in women and 1800 per 100,000 (1405–2040) in men. Over the past three decades, there has been a noticeable rise in the incidence of urolithiasis among females worldwide. The global ratio of female to male incident cases stood at 1:2.21 in 1990, decreased slightly to 1:2.05 in 2010, and further decreased to 1:2.04 by 2021. Adjusting for population size and ageing, the global ratio of age-standardised incidence rates for females to males was 1:2.3 in 1990, declined to 1:2.1 in 2010, and continued to decline to 1:2.07 in 2021.

**Fig. 2:**
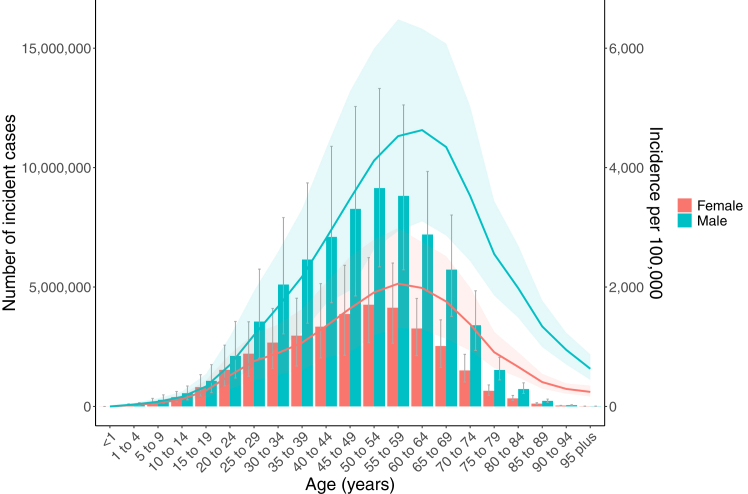
Incidence of Urolithiasis by age and sex, 2021 Bars represent mean incidence and age-standardised incidence rate, and error bars represent 95% uncertainty intervals.

The number of incident cases was highest in the 50–54 age group in both women (4.7 million [95% UI 2.8–6.9]) and men (9.6 million [5.7–14.1]). The age-specific incidence rates, however, peaked in the 55–59 age group in both sexes (Fig. 2).

Globally, urolithiasis accounted for 17,700 (95% UI 13,700–21,300) deaths in 2021 in both sexes. There were 9400 deaths (6600–12,200) in males and 8300 deaths (6300–10,200) in females. Globally, deaths due to urolithiasis increased by 60.3% (41.5–84.7) between 2000 and 2021 in both sexes. The global age-standardised death rate in 2021 was 0.212 deaths (0.165–0.255) per 100,000, which was an 8.5% (−6.6 to 19.1) decrease from 0.22 deaths (0.15–0.26) per 100,000 in 2000.

Urolithiasis accounted for 694,000 DALYs (95% UI 567,000–851,000) globally in both sexes in 2021. Of these, 42% came from YLDs and 58% from YLLs. Globally, the total number of DALYs increased by 34.5% (24.6–47.3) from 2000 to 2021 for both sexes. In 2021, the global age-standardised rate of DALYs was 8.15 per 100,000 (6.68–9.99), which decreased by 15.1% (6.8–21.3) between 2000 and 2021, indicating improved overall burden of urolithiasis, after accounting for the impact of age. Between 2000 and 2021, the global age-standardised rate of female and male DALYs of urolithiasis decreased by 10.8% (−2.2 to 18.2) and 18.4% (7.3–26.2), respectively (Tables 1 and 2, and Supplementary File Table S6).

**Table 1:** Global, super-region, and country-level incidence of acute urolithiasis for both sexes and all locations, and percentage change in 2000 and 2021.

location_name	count.2000	rate.2000	count.2021	rate.2021	Percentage change in counts between 2000 and 2021	Percentage change in age-standardised rates between 2000 and 2021
**Global**	**81,300,000 (66,700,000–99,900,000)**	**1450 (1200–1780)**	**106,000,000 (88,300,000–129,000,000)**	**1240 (1030–1510)**	**30.3 (27.4–33.2)**	**−14.2 (−16.8 to 11.4)**
**Central Europe, Eastern Europe, and Central Asia**	**14,500,000 (12,100,000–17,500,000)**	**2970 (2470–3570)**	**13,600,000 (11,500,000–16,000,000)**	**2500 (2100–2960)**	**−6.65 (−9.20 to** −**4.00)**	**−15.9 (−18.3 to 13.2)**
Central Asia	1,150,000 (949,000–1,380,000)	1790 (1500–2170)	1,790,000 (1,520,000–2,150,000)	1870 (1590–2210)	56.4 (49.9–63.8)	4.36 (1.62–7.62)
Armenia	82,700 (69,400–98,200)	2370 (2000–2800)	122,000 (106,000–139,000)	3090 (2690–3560)	47.1 (37.5–59.4)	30.2 (22.8–38.8)
Azerbaijan	114,000 (90,900–142,000)	1500 (1190–1880)	181,000 (145,000–228,000)	1500 (1200–1870)	58.9 (46.0–74.1)	0.282 (−3.84 to 4.08)
Georgia	74,100 (58,700–93,400)	1380 (1100–1730)	53,200 (48,000–59,200)	1190 (1070–1320)	−28.2 (−38.6 to 15.1)	−13.9 (−26.0 to 1.37)
Kazakhstan	338,000 (287,000–393,000)	2340 (2000–2720)	540,000 (470,000–625,000)	2740 (2390–3150)	59.7 (50.4–70.0)	17.3 (10.5–24.4)
Kyrgyzstan	75,200 (62,600–91,000)	1820 (1500–2200)	105,000 (85,400–129,000)	1620 (1340–1990)	39.1 (31.4–48.3)	−10.8 (−14.7 to −6.06)
Mongolia	29,300 (23,600–36,300)	1500 (1200–1870)	47,300 (38,600–59,100)	1460 (1190–1800)	61.6 (48.8–74.0)	−2.61 (−7.43 to 1.08)
Tajikistan	70,300 (57,200–86,600)	1530 (1250–1870)	143,000 (118,000–176,000)	1610 (1350–1940)	103 (92.2–115)	5.17 (0.325–10.2)
Turkmenistan	50,700 (41,200–62,900)	1470 (1170–1820)	76,700 (62,400–94,500)	1500 (1230–1840)	51.1 (41.8–62.3)	2.18 (−5.53 to 8.49)
Uzbekistan	312,000 (251,000–390,000)	1550 (1250–1950)	525,000 (419,000–655,000)	1540 (1230–1920)	68.5 (56.6–81.1)	−0.648 (−4.28 to 3.32)
Central Europe	2,100,000 (1,730,000–2,580,000)	1420 (1180–1720)	1,580,000 (1,310,000–1,890,000)	1040 (867–1240)	−24.8 (−27.3 to 21.8)	−26.8 (−28.8 to 24.7)
Albania	36,300 (29,500–44,900)	1170 (952–1450)	37,000 (29,100–46,400)	1100 (893–1380)	1.94 (−5.60 to 10.3)	−5.87 (−9.56 to −2.19)
Bosnia and Herzegovina	53,900 (42,800–67,500)	1150 (928–1410)	46,200 (36,800–58,100)	1070 (868–1330)	−14.2 (−18.6 to 9.07)	−6.80 (−10.5 to −2.77)
Bulgaria	138,000 (112,000–173,000)	1310 (1070–1600)	94,700 (76,200–117,000)	1070 (864–1310)	−31.1 (−40.5 to 26.4)	−18.5 (−25.7 to 14.0)
Croatia	98,300 (90,700–106,000)	1650 (1530–1780)	72,200 (60,300–86,100)	1250 (1040–1470)	−26.6 (−36.8 to 15.7)	−24.6 (−34.2 to 14.0)
Czechia	179,000 (148,000–220,000)	1390 (1150–1660)	161,000 (131,000–198,000)	1170 (946–1430)	−10.0 (−17.0 to 4.19)	−15.8 (−21.0 to 11.5)
Hungary	208,000 (173,000–251,000)	1540 (1290–1830)	186,000 (155,000–220,000)	1350 (1130–1610)	−10.5 (−18.1 to 3.29)	−12.5 (−19.4 to 6.28)
Montenegro	8450 (6750–10,600)	1180 (953–1470)	8650 (6820–10,900)	1120 (903–1380)	2.32 (−2.36 to 7.33)	−5.42 (−8.50 to −2.06)
North Macedonia	27,200 (21,800–34,000)	1180 (960–1480)	31,600 (25,000–40,000)	1120 (899–1390)	16.0 (10.7–21.3)	−5.52 (−8.49 to −2.07)
Poland	700,000 (552,000–894,000)	1560 (1240–1970)	405,000 (351,000–464,000)	811 (708–919)	−42.1 (−49.2 to 33.5)	−48.1 (−53.8 to 40.9)
Romania	320,000 (255,000–401,000)	1200 (973–1480)	277,000 (218,000–352,000)	1120 (900–1400)	−13.5 (−17.8 to −8.59)	−5.98 (−9.29 to −2.68)
Serbia	133,000 (106,000–164,000)	1140 (914–1410)	122,000 (97,900–150,000)	1070 (863–1310)	−8.30 (−12.0 to 4.33)	−6.20 (−10.6 to −1.78)
Slovakia	129,000 (101,000–149,000)	2040 (1640–2370)	83,500 (67,500–104,000)	1210 (978–1470)	−35.0 (−44.1 to 24.1)	−41.0 (−49.3 to 31.4)
Slovenia	33,700 (27,700–41,000)	1350 (1110–1640)	28,700 (23,200–36,000)	1050 (849–1290)	−14.9 (−23.5 to 6.56)	−22.1 (−29.2 to 15.9)
Eastern Europe	11,300,000 (9,310,000–13,600,000)	4090 (3400–4920)	10,200,000 (8,620,000–12,000,000)	3560 (2990–4230)	−9.69 (−12.5 to 6.66)	−13.1 (−16.1 to 9.91)
Belarus	437,000 (363,000–522,000)	3470 (2870–4160)	464,000 (394,000–551,000)	3520 (2940–4220)	6.16 (0.621–12.6)	1.28 (−3.08 to 5.88)
Estonia	61,100 (50,500–73,500)	3380 (2800–4080)	54,300 (44,200–67,500)	3130 (2540–3920)	−11.1 (−16.8 to −5.12)	−7.38 (−13.0 to 1.55)
Latvia	123,000 (109,000–140,000)	3920 (3440–4490)	93,800 (79,600–112,000)	3440 (2860–4170)	−23.9 (−29.7 to 18.4)	−12.3 (−19.5 to −5.27)
Lithuania	173,000 (157,000–193,000)	3920 (3570–4380)	119,000 (98,700–145,000)	3160 (2610–3870)	−30.9 (−39.2 to 22.3)	−19.3 (−29.7 to 8.84)
Republic of Moldova	148,000 (121,000–182,000)	3100 (2560–3810)	148,000 (123,000–180,000)	2990 (2490–3680)	−0.148 (−4.37 to 4.36)	−3.43 (−7.04 to 0.213)
Russian Federation	7,570,000 (6,200,000–9,090,000)	4090 (3390–4920)	7,000,000 (5,940,000–8,190,000)	3530 (2960–4180)	−7.53 (−11.0 to 3.90)	−13.9 (−16.9 to 10.6)
Ukraine	2,780,000 (2,290,000–3380,000)	4300 (3550–5270)	2,320,000 (1,940,000–2750,000)	3770 (3150–4480)	−16.6 (−21.5 to 11.4)	−12.5 (−17.9 to −7.11)
**High-income**	**16,100,000 (13,300,000**–**19,800,000)**	**1360 (1130**–**1670)**	**18,600,000 (15,500,000**–**22,500,000)**	**1270 (1060**–**1530)**	**16.0 (11.0**–**20.5)**	**−6.12 (−8.68 to −4.00)**
Australasia	352,000 (284,000–436,000)	1310 (1060–1620)	511,000 (415,000–632,000)	1280 (1040–1590)	45.2 (38.9–51.7)	−2.16 (−6.09 to 1.16)
Australia	293,000 (235,000–368,000)	1310 (1050–1640)	419,000 (336,000–525,000)	1260 (1010–1590)	43.1 (36.3–50.7)	−3.52 (−7.99 to 0.601)
New Zealand	58,800 (49,800–70,000)	1310 (1120–1560)	91,500 (77,800–107,000)	1380 (1170–1610)	55.8 (48.7–63.8)	4.73 (1.33–8.52)
High-income Asia Pacific	3,370,000 (2,650,000–4,370,000)	1450 (1160–1860)	3,700,000 (3,000,000–4,550,000)	1380 (1130–1700)	9.82 (3.64–17.0)	−4.98 (−9.23 to 0.227)
Brunei Darussalam	3980 (3110–5170)	1480 (1200–1840)	7070 (5630–8930)	1450 (1190–1790)	77.8 (65.3–91.4)	−1.68 (−6.01 to 2.57)
Japan	2,600,000 (2,040,000–3,390,000)	1490 (1180–1920)	2,570,000 (2,110,000–3,120,000)	1410 (1160–1720)	−1.20 (−8.80 to 7.35)	−5.40 (−11.2 to 1.61)
Republic of Korea	701,000 (554,000–912,000)	1340 (1070–1710)	1,010,000 (804,000–1,300,000)	1320 (1070–1660)	44.4 (31.1–57.7)	−1.41 (−5.08 to 2.81)
Singapore	61,600 (47,600–79,700)	1380 (1090–1770)	109,000 (85,100–139,000)	1320 (1050–1680)	76.8 (61.9–91.5)	−4.06 (−7.66 to −0.224)
High-income North America	4,420,000 (3,710,000–5,350,000)	1220 (1020–1480)	4,790,000 (4,050,000–5,650,000)	965 (824–1130)	8.43 (0.997–16.1)	−20.9 (−24.5 to 17.9)
Canada	372,000 (302,000–464,000)	1010 (809–1260)	560,000 (453,000–696,000)	1060 (861–1310)	50.5 (40.4–62.9)	4.97 (1.10–10.0)
Greenland	558 (441–715)	968 (771–1220)	629 (492–805)	914 (729–1140)	12.8 (1.36–25.7)	−5.63 (−9.68 to −1.26)
United States of America	4,040,000 (3,400,000–4,900,000)	1240 (1040–1500)	4,230,000 (3,580,000–4,970,000)	954 (817–1110)	4.56 (−2.86 to 12.3)	−23.3 (−27.1 to 20.1)
Southern Latin America	985,000 (831,000–1,200,000)	1790 (1520–2190)	1,500,000 (1,260,000–1,820,000)	1970 (1650–2390)	52.8 (46.9–59.7)	9.68 (5.29–14.2)
Argentina	631,000 (500,000–815,000)	1760 (1390–2280)	834,000 (663,000–1,060,000)	1660 (1320–2120)	32.3 (26.3–38.1)	−5.32 (−10.0 to 1.47)
Chile	293,000 (271,000–314,000)	1860 (1720–1990)	603,000 (529,000–690,000)	2690 (2350–3070)	106 (84.9–129)	44.2 (30.1–59.2)
Uruguay	60,700 (48,000–76,900)	1760 (1390–2240)	66,600 (52,900–85,100)	1690 (1350–2200)	9.71 (5.51–13.9)	−3.53 (−6.75 to −0.0614)
Western Europe	6,960,000 (5,790,000–8,490,000)	1370 (1130–1680)	8,140,000 (6,710,000–9,950,000)	1370 (1130–1660)	17.0 (12.0–21.6)	0.00853 (−2.25 to 1.97)
Andorra	1160 (931–1490)	1370 (1090–1730)	1730 (1370–2190)	1340 (1080–1700)	48.7 (36.5–61.0)	−1.74 (−5.95 to 3.19)
Austria	318,000 (290,000–341,000)	3040 (2780–3250)	221,000 (183,000–273,000)	1800 (1480–2220)	−30.5 (−41.2 to 15.6)	−40.7 (−49.9 to 28.7)
Belgium	243,000 (196,000–301,000)	1870 (1530–2320)	204,000 (163,000–255,000)	1340 (1080–1680)	−15.7 (−26.0 to 6.76)	−28.4 (−36.6 to 21.7)
Cyprus	9470 (7860–11500)	911 (757–1110)	18,800 (15,000–23,600)	1050 (845–1320)	98.4 (84.3–113)	15.3 (6.38–24.5)
Denmark	87,300 (78,200–98,000)	1300 (1170–1460)	101,000 (82,800–124,000)	1230 (1000–1520)	15.6 (−1.16 to 32.5)	−5.30 (−19.3 to 8.35)
Finland	49,300 (45,800–53,500)	706 (658–772)	79,600 (63,000–101,000)	1080 (855–1370)	61.6 (32.5–96.3)	52.7 (25.5–86.7)
France	908,000 (718,000–1,170,000)	1240 (984–1580)	1,020,000 (810,000–1,280,000)	1190 (959–1500)	12.1 (4.46–19.5)	−3.55 (−7.93 to 1.05)
Germany	1,250,000 (1,000,000–1,580,000)	1130 (911–1450)	1,330,000 (1,070,000–1,680,000)	1120 (899–1410)	5.99 (−0.348 to 13.2)	−1.38 (−5.69 to 2.87)
Greece	192,000 (153,000–244,000)	1350 (1090–1730)	221,000 (181,000–269,000)	1560 (1300–1880)	15.3 (4.58–27.4)	15.3 (5.17–27.4)
Iceland	3600 (2890–4550)	1160 (937–1480)	5220 (4260–6550)	1180 (955–1480)	45.0 (35.1–56.3)	1.27 (−2.95 to 6.43)
Ireland	53,700 (42,700–69,300)	1250 (1000–1620)	75,200 (59,800–95,900)	1210 (969–1540)	40.1 (33.2–47.7)	−3.59 (−7.28 to 1.04)
Israel	78,300 (62,600–101,000)	1260 (1010–1620)	127,000 (102,000–159,000)	1250 (999–1580)	62.3 (53.7–71.6)	−0.602 (−5.09 to 3.58)
Italy	1,250,000 (1,030,000–1,520,000)	1660 (1370–2030)	1,340,000 (1,130,000–1,580,000)	1510 (1300–1760)	7.51 (−1.74 to 15.8)	−8.99 (−16.6 to −2.25)
Luxembourg	11,700 (9540–14,200)	2170 (1770–2620)	11,600 (9120–14,900)	1350 (1070–1700)	−0.761 (−12.7 to 11.2)	−37.9 (−44.8 to 31.8)
Malta	8330 (6780–10,300)	1700 (1400–2060)	8320 (6710–10,300)	1350 (1100–1690)	−0.204 (−10.9 to 10.9)	−20.6 (−28.1 to 13.6)
Monaco	585 (460–760)	1240 (984–1610)	641 (498–837)	1180 (945–1510)	9.60 (3.64–15.3)	−5.34 (−9.49 to −1.62)
Netherlands	277,000 (224,000–352,000)	1370 (1110–1730)	314,000 (252,000–395,000)	1310 (1060–1630)	13.3 (6.03–21.8)	−4.39 (−8.88 to 0.752)
Norway	130,000 (106,000–164,000)	2370 (1920–2990)	154,000 (123,000–192,000)	2170 (1750–2720)	18.0 (14.6–21.5)	−8.49 (−10.3 to 6.82)
Portugal	143,000 (114,000–183,000)	1080 (864–1390)	173,000 (147,000–201,000)	1150 (984–1330)	21.0 (6.85–39.3)	6.29 (−5.79 to 21.7)
San Marino	456 (371–581)	1290 (1050–1650)	600 (481–759)	1280 (1040–1600)	31.7 (23.6–39.1)	−0.788 (−5.29 to 3.67)
Spain	574,000 (464,000–731,000)	1120 (900–1430)	856,000 (710,000–1,030,000)	1360 (1140–1630)	49.1 (28.0–71.9)	22.1 (6.46–41.0)
Sweden	134,000 (108,000–170,000)	1150 (927–1460)	177,000 (143,000–221,000)	1260 (1020–1570)	31.9 (26.0–38.2)	9.02 (5.21–12.9)
Switzerland	191,000 (178,000–205,000)	2040 (1910–2200)	157,000 (125,000–198,000)	1290 (1040–1640)	−17.9 (−33.2 to 1.90)	−36.7 (−48.2 to 22.7)
United Kingdom	1,040,000 (875,000–1,240,000)	1410 (1180–1690)	1,540,000 (1,280,000–1,870,000)	1710 (1430–2080)	48.3 (41.9–53.9)	21.1 (17.2–24.6)
**Latin America and Caribbean**	**3,900,000 (3,270,000**–**4,770,000)**	**977 (822**–**1180)**	**7,650,000 (6,400,000**–**9,180,000)**	**1190 (993**–**1420)**	**96.5 (89.3**–**104)**	**21.4 (18.8**–**24.0)**
Andean Latin America	630,000 (526,000–777,000)	1650 (1380–2040)	1,150,000 (961,000–1,400,000)	1730 (1440–2100)	82.4 (75.2–90.4)	4.63 (1.25–8.42)
Bolivia (Plurinational State of)	101,000 (80,600–129,000)	1580 (1250–2030)	175,000 (140,000–220,000)	1540 (1240–1920)	73.2 (65.4–81.2)	−2.74 (−7.58 to 1.55)
Ecuador	179,000 (164,000–196,000)	1740 (1590–1910)	382,000 (338,000–423,000)	2150 (1900–2380)	114 (98.6–128)	23.2 (15.0–30.9)
Peru	350,000 (279,000–451,000)	1630 (1300–2100)	593,000 (471,000–762,000)	1590 (1260–2040)	69.2 (61.1–76.6)	−2.69 (−6.77 to 1.38)
Caribbean	404,000 (328,000–508,000)	1100 (890–1380)	678,000 (561,000–830,000)	1290 (1070–1570)	67.8 (58.4–77.9)	17.5 (12.3–23.0)
Antigua and Barbuda	722 (577–912)	1010 (811–1280)	1200 (956–1500)	1080 (873–1340)	66.1 (52.4–79.3)	6.60 (1.99–11.5)
Bahamas	2920 (2340–3710)	1030 (829–1310)	4910 (3960–6160)	1080 (878–1340)	68.1 (55.6–80.6)	4.53 (−1.38 to 9.55)
Barbados	3180 (2530–4020)	1120 (904–1420)	5370 (4360–6570)	1270 (1050–1550)	68.6 (55.6–83.4)	12.9 (7.66–19.1)
Belize	1760 (1440–2200)	1070 (871–1350)	4580 (3730–5610)	1180 (962–1450)	160 (145–175)	10.7 (4.90–17.1)
Bermuda	826 (653–1050)	1050 (838–1330)	1040 (824–1340)	1100 (885–1390)	26.4 (16.2–36.8)	5.08 (1.38–9.30)
Cuba	149,000 (121,000–189,000)	1160 (942–1480)	254,000 (208,000–310,000)	1520 (1260–1830)	70.2 (53.8–88.8)	30.3 (21.5–39.9)
Dominica	623 (497–783)	984 (785–1250)	817 (644–1040)	1010 (811–1280)	31.0 (20.4–42.1)	3.08 (−1.70 to 7.99)
Dominican Republic	69,300 (55,800–87200)	1010 (805–1290)	108,000 (85,500–137,000)	982 (780–1250)	55.6 (48.3–62.4)	−2.48 (−6.74 to 1.23)
Grenada	1100 (903–1360)	1240 (1030–1540)	2140 (1820–2550)	1740 (1490–2060)	94.6 (80.4–113)	40.2 (30.8–51.6)
Guyana	6930 (5670–8650)	1170 (952–1430)	10,200 (8480–12,100)	1340 (1120–1600)	47.1 (36.1–57.3)	15.1 (8.50–21.9)
Haiti	60,000 (48,900–73,600)	1040 (848–1270)	115,000 (95,100–139,000)	1100 (920–1310)	92.3 (82.4–104)	6.02 (0.130–13.4)
Jamaica	23,100 (18,700–29,000)	1030 (832–1310)	33,900 (27,400–42,200)	1100 (891–1360)	46.9 (37.6–57.6)	6.73 (0.0531–13.0)
Puerto Rico	43,500 (34,200–56,100)	1020 (804–1300)	45,900 (36,000–58,900)	1030 (824–1310)	5.43 (−1.72 to 12.2)	1.26 (−3.39 to 5.86)
Saint Kitts and Nevis	423 (338–529)	1030 (831–1290)	857 (680–1080)	1100 (890–1380)	102 (82.9–124)	6.59 (1.94–12.2)
Saint Lucia	1400 (1130–1750)	1070 (862–1350)	2670 (2140–3370)	1150 (943–1450)	90.5 (75.4–105)	8.00 (2.84–13.3)
Saint Vincent and the Grenadines	1010 (816–1250)	1100 (892–1370)	1830 (1510–2240)	1320 (1100–1610)	82.1 (67.3–99.7)	20.4 (12.3–27.8)
Suriname	4950 (4110–6070)	1270 (1050–1560)	10,800 (9150–12,800)	1610 (1370–1900)	118 (100–137)	26.8 (17.3–36.0)
Trinidad and Tobago	17,400 (14,300–21,600)	1410 (1150–1730)	48,700 (42,600–56,100)	2570 (2240–2980)	181 (147–216)	82.0 (61.2–105)
United States Virgin Islands	1900 (1540–2370)	1450 (1200–1770)	3280 (2840–3770)	2280 (1960–2640)	72.8 (55.4–92.3)	56.9 (44.1–70.2)
Central Latin America	1,410,000 (1,170,000–1,740,000)	891 (739–1090)	3,140,000 (2,600,000–3,790,000)	1180 (978–1420)	123 (113–133)	32.1 (28.1–35.6)
Colombia	288,000 (228,000–368,000)	837 (669–1060)	447,000 (357,000–562,000)	826 (659–1040)	55.3 (46.2–65.2)	−1.28 (−6.43 to 3.70)
Costa Rica	28,600 (22,600–37,200)	847 (670–1100)	42,500 (34,100–54,400)	792 (635–1020)	48.4 (38.4–58.1)	−6.58 (−10.4 to −2.11)
El Salvador	36,600 (29,500–45900)	819 (651–1030)	47,500 (38,200–61,400)	769 (614–989)	29.9 (24.3–36.7)	−6.10 (−9.80 to −2.28)
Guatemala	58,600 (47,700–73,700)	817 (652–1040)	106,000 (85,900–131,000)	778 (633–970)	80.5 (72.3–89.0)	−4.70 (−9.43 to −0.164)
Honduras	36,100 (29,100–44,600)	887 (711–1110)	73,600 (59,900–91,300)	888 (721–1100)	104 (95.6–114)	0.138 (−3.92 to 5.06)
Mexico	726,000 (618,000–872,000)	911 (773–1080)	2,030,000 (1,700,000–2,410,000)	1470 (1240–1740)	180 (168–194)	61.5 (56.3–66.9)
Nicaragua	27,700 (22,400–34,800)	824 (661–1040)	48,800 (39,000–62,300)	791 (634–1000)	76.3 (66.8–85.7)	−4.02 (−8.46 to 0.886)
Panama	21,600 (17,300–27,600)	846 (671–1070)	35,000 (27,900–44,400)	795 (634–1000)	61.8 (52.9–69.7)	−6.03 (−9.72 to −2.24)
Venezuela (Bolivarian Republic of)	187,000 (149,000–235,000)	981 (803–1220)	310,000 (252,000–387,000)	1020 (832–1260)	66.2 (54.8–77.4)	3.43 (−1.14 to 8.07)
Tropical Latin America	1,450,000 (1,240,000–1740,000)	883 (751–1050)	2,680,000 (2,260,000–3,190,000)	1030 (875–1220)	84.8 (75.9–94.2)	16.9 (13.4–20.5)
Brazil	1,420,000 (1,210,000–1,700,000)	884 (753–1050)	2,630,000 (2,220,000–3,120,000)	1040 (880–1230)	85.2 (76.0–95.0)	17.4 (13.8–21.1)
Paraguay	33,600 (26,900–42,400)	843 (671–1060)	56,500 (45,600–70,900)	815 (657–1020)	68.1 (60.5–75.6)	−3.27 (−7.62 to 0.977)
**North Africa and Middle East**	**2,820,000 (2,250,000**–**3,590,000)**	**827 (667**–**1050)**	**5,300,000 (4,220,000**–**6,800,000)**	**851 (687**–**1080)**	**87.9 (79.0**–**95.5)**	**2.93 (1.50**–**4.43)**
Afghanistan	75,000 (59,200–94,500)	742 (593–941)	162,000 (128,000–203,000)	763 (608–959)	116 (96.1–138)	2.92 (−2.42 to 7.90)
Algeria	203,000 (161,000–259,000)	808 (647–1030)	371,000 (294,000–485,000)	824 (656–1050)	82.6 (69.2–97.3)	2.01 (−2.70 to 6.99)
Bahrain	5640 (4290–7470)	891 (710–1150)	17,700 (13,600–23,000)	956 (762–1210)	215 (191–241)	7.36 (2.85–11.9)
Egypt	458,000 (367,000–588,000)	811 (650–1040)	790,000 (637,000–1,010,000)	832 (672–1060)	72.4 (65.3–80.8)	2.70 (−1.00 to 7.60)
Iran (Islamic Republic of)	481,000 (381,000–610,000)	879 (704–1120)	832,000 (663,000–1,070,000)	857 (693–1080)	73.0 (60.4–83.4)	−2.47 (−4.22 to −0.751)
Iraq	154,000 (124,000–192,000)	848 (677–1070)	313,000 (250,000–395,000)	839 (674–1060)	103 (91.2–115)	−1.02 (−5.08 to 2.92)
Jordan	28,900 (23,000–37,100)	817 (653–1050)	125,000 (101,000–158,000)	1060 (854–1340)	332 (290–372)	29.2 (21.7–36.3)
Kuwait	17,300 (13,200–22,700)	925 (738–1190)	51,000 (39,100–67,400)	874 (702–1110)	194 (166–220)	−5.44 (−9.20 to −1.52)
Lebanon	26,400 (21,200–33,500)	794 (641–1020)	48,900 (39,400–62,400)	813 (655–1030)	85.3 (74.8–95.8)	2.42 (−1.98 to 7.24)
Libya	32,600 (26,000–41,800)	828 (666–1080)	65,200 (50,700–85,200)	830 (667–1060)	100 (79.3–119)	0.164 (−4.52 to 5.36)
Morocco	200,000 (159,000–257,000)	792 (633–1020)	317,000 (254,000–403,000)	806 (649–1020)	58.3 (48.0–69.4)	1.74 (−3.28 to 6.28)
Oman	16,700 (13,200–21,600)	907 (727–1160)	48,300 (36,800–63,700)	953 (764–1220)	189 (168–208)	5.09 (0.647–9.97)
Palestine	15,900 (12,700–20,400)	800 (642–1040)	34,700 (27,900–44,700)	826 (669–1050)	118 (106–130)	3.27 (−2.30 to 7.57)
Qatar	6320 (4730–8530)	975 (775–1240)	38,400 (28,500–51,400)	1020 (816–1320)	507 (467–549)	4.22 (−0.899 to 9.46)
Saudi Arabia	153,000 (121,000–200,000)	886 (708–1140)	404,000 (309,000–532,000)	912 (729–1150)	164 (147–184)	2.95 (−2.18 to 7.94)
Sudan	146,000 (118,000–185,000)	803 (651–1020)	271,000 (216,000–344,000)	805 (645–1010)	85.7 (77.4–97.4)	0.273 (−4.09 to 6.49)
Syrian Arab Republic	97,800 (77,900–124,000)	829 (666–1060)	122,000 (95,400–158,000)	807 (640–1040)	25.0 (9.31–40.0)	−2.74 (−6.92 to 1.60)
Tunisia	72,100 (57,800–92,400)	807 (650–1030)	111,000 (88,700–141,000)	818 (657–1030)	54.3 (42.4–65.8)	1.27 (−2.70 to 5.63)
Türkiye	505,000 (408,000–648,000)	811 (652–1040)	825,000 (656,000–1,060,000)	862 (691–1090)	63.3 (53.7–74.6)	6.34 (1.78–10.6)
United Arab Emirates	32,900 (24,800–44,000)	978 (777–1260)	150,000 (110,000–204,000)	1010 (811–1280)	357 (294–427)	3.70 (−1.15 to 9.15)
Yemen	93,000 (74,400–117,000)	799 (640–1020)	201,000 (160,000–258,000)	790 (637–1010)	116 (105–130)	−1.11 (−5.65 to 3.46)
**South Asia**	**13,000,000 (10,500,000**–**16,000,000)**	**1210 (987**–**1490)**	**22,800,000 (18,600,000**–**28,200,000)**	**1250 (1020**–**1530)**	**75.6 (72.1**–**79.1)**	**3.18 (0.442**–**6.02)**
Bangladesh	1,020,000 (815,000–1,280,000)	1060 (858–1330)	1,780,000 (1,440,000–2,200,000)	1100 (889–1360)	73.8 (63.4–85.5)	3.34 (−0.917 to 7.26)
Bhutan	5340 (4330–6660)	1060 (865–1310)	8500 (6890–10,700)	1120 (916–1400)	59.1 (51.2–67.1)	5.40 (1.45–9.66)
India	10,600,000 (8,540,000–13,000,000)	1220 (1000–1510)	18,500,000 (15,100,000–22,900,000)	1280 (1060–1580)	75.4 (71.5–79.1)	4.88 (1.68–8.31)
Nepal	189,000 (152,000–237,000)	1040 (840–1300)	310,000 (253,000–379,000)	1070 (878–1310)	63.8 (56.1–71.9)	3.16 (−1.34 to 7.24)
Pakistan	1,210,000 (977,000–1,490,000)	1220 (997–1510)	2,190,000 (1,790,000–2,670,000)	1130 (939–1390)	80.7 (75.8–86.0)	−7.39 (−10.3 to 4.32)
**Southeast Asia, East Asia, and Oceania**	**28,000,000 (22,700,000**–**35,000,000)**	**1580 (1280**–**1970)**	**32,500,000 (26,900,000**–**39,600,000)**	**1150 (962**–**1400)**	**16.0 (9.86**–**21.6)**	**−27.2 (−30.6 to 22.9)**
East Asia	20,000,000 (16,200,000–25,300,000)	1510 (1210–1890)	20,100,000 (16,500,000–24,800,000)	978 (813–1190)	0.282 (−5.86 to 5.71)	−35.1 (−38.4 to 30.7)
China	19,500,000 (15,800,000–24,600,000)	1520 (1220–1910)	19,100,000 (15,700,000–23,700,000)	965 (801–1180)	−1.71 (−7.74 to 3.51)	−36.5 (−39.8 to 32.2)
Democratic People's Republic of Korea	290,000 (238,000–359,000)	1190 (970–1480)	426,000 (346,000–521,000)	1240 (1020–1510)	46.6 (38.6–55.9)	4.26 (−0.836 to 8.98)
Taiwan (Province of China)	283,000 (224,000–365,000)	1170 (932–1510)	538,000 (448,000–641,000)	1480 (1250–1740)	89.9 (67.2–116)	27.1 (14.3–41.0)
Oceania	57,300 (46,000–72,700)	971 (778–1230)	106,000 (86,000–134,000)	934 (763–1160)	85.5 (79.5–92.0)	−3.77 (−6.89 to −0.571)
American Samoa	473 (378–591)	1050 (858–1320)	536 (422–679)	1000 (801–1250)	13.2 (4.07–22.0)	−4.76 (−9.48 to −0.587)
Cook Islands	182 (146–232)	996 (795–1270)	215 (168–277)	983 (788–1240)	17.8 (8.50–26.0)	−1.25 (−5.36 to 3.43)
Fiji	6660 (5310–8500)	951 (756–1210)	8770 (7100–11,000)	933 (756–1170)	31.7 (24.8–40.2)	−1.85 (−7.31 to 2.72)
Guam	1540 (1220–1950)	1030 (830–1310)	1820 (1430–2320)	973 (785–1230)	18.2 (8.17–29.6)	−5.57 (−9.58 to −0.713)
Kiribati	642 (516–811)	1020 (822–1260)	1000 (825–1240)	977 (808–1190)	56.4 (49.6–63.9)	−3.81 (−8.23 to 1.00)
Marshall Islands	338 (269–423)	1010 (818–1260)	517 (415–643)	1000 (812–1230)	53.2 (44.4–62.4)	−0.671 (−5.52 to 4.74)
Micronesia (Federated States of)	770 (617–959)	1030 (835–1280)	1010 (821–1240)	1040 (847–1270)	31.2 (22.4–40.2)	0.352 (−4.25 to 4.67)
Nauru	85.4 (68.8–108)	1100 (892–1370)	92.4 (74.6–115)	1080 (888–1340)	8.16 (3.90–12.7)	−1.86 (−5.45 to 2.80)
Niue	20.7 (16.5–26.4)	1020 (825–1310)	20.6 (16.3–26.2)	1030 (819–1300)	−0.249 (−4.75 to 4.25)	0.348 (−3.68 to 4.31)
Northern Mariana Islands	646 (493–857)	1040 (820–1330)	607 (470–789)	1000 (803–1270)	−6.09 (−20.2 to 10.7)	−3.89 (−8.31 to 1.34)
Palau	213 (166–280)	1060 (846–1360)	271 (210–347)	1070 (855–1350)	27.4 (14.1–41.6)	1.03 (−3.30 to 6.28)
Papua New Guinea	36,000 (28,500–45,300)	956 (761–1190)	76,000 (61,300–95,400)	917 (746–1150)	111 (103–121)	−4.06 (−7.93 to 0.202)
Samoa	1320 (1060–1660)	1030 (820–1290)	1750 (1430–2200)	1000 (809–1260)	32.7 (25.8–39.7)	−2.18 (−6.01 to 2.71)
Solomon Islands	3010 (2460–3740)	1060 (871–1330)	5580 (4470–6850)	1060 (861–1290)	85.4 (74.3–97.6)	−0.622 (−5.87 to 4.76)
Tokelau	12.6 (10.1–15.7)	964 (767–1210)	13.7 (10.8–17.5)	981 (774–1260)	8.47 (2.96–14.6)	1.85 (−2.62 to 5.77)
Tonga	737 (596–922)	984 (788–1250)	874 (707–1090)	986 (795–1230)	18.5 (13.1–24.1)	0.245 (−4.52 to 4.75)
Tuvalu	86.0 (69.9–108)	984 (801–1240)	117 (95.4–146)	1010 (813–1250)	36.6 (30.5–44.3)	2.35 (−1.89 to 6.75)
Vanuatu	1260 (1030–1600)	968 (780–1210)	2350 (1900–2960)	941 (765–1170)	86.0 (76.5–95.2)	−2.74 (−7.88 to 2.27)
Southeast Asia	7,920,000 (6,450,000–9,660,000)	1820 (1490–2230)	12,300,000 (10,200,000–14,700,000)	1630 (1360–1940)	55.5 (49.4–62.1)	−10.5 (−13.7 to −6.50)
Cambodia	114,000 (92,600–138,000)	1390 (1150–1680)	225,000 (187,000–271,000)	1450 (1220–1740)	97.6 (87.6–109)	4.23 (−0.872 to 8.81)
Indonesia	3,190,000 (2,590,000–3,900,000)	1890 (1550–2310)	4,160,000 (3,520,000–4,910,000)	1390 (1190–1620)	30.4 (23.6–39.2)	−26.3 (−30.7 to 21.6)
Lao People's Democratic Republic	50,600 (42,000–61,200)	1480 (1240–1770)	84,800 (69,600–103,000)	1340 (1110–1620)	67.4 (58.6–75.1)	−9.70 (−14.6 to −5.48)
Malaysia	261,000 (203,000–329,000)	1340 (1060–1700)	431,000 (341,000–546,000)	1300 (1040–1640)	65.1 (56.0–75.2)	−2.92 (−8.10 to 2.03)
Maldives	2510 (2000–3180)	1400 (1090–1800)	8430 (6590–11,000)	1470 (1170–1890)	235 (208–264)	4.79 (−0.860 to 11.4)
Mauritius	16,700 (12,800–21,400)	1390 (1090–1790)	22,900 (17,900–29,600)	1330 (1050–1700)	37.1 (24.7–51.9)	−4.16 (−8.78 to 1.07)
Myanmar	567,000 (465,000–690,000)	1570 (1290–1880)	808,000 (673,000–968,000)	1430 (1200–1710)	42.6 (34.9–51.2)	−8.70 (−13.1 to 4.35)
Philippines	1,380,000 (1,140,000–1,660,000)	2530 (2110–3040)	2,560,000 (2,170,000–3,000,000)	2500 (2120–2930)	85.1 (78.8–91.5)	−1.32 (−5.72 to 3.42)
Seychelles	1060 (860–1330)	1430 (1160–1790)	1970 (1590–2450)	1540 (1250–1890)	85.4 (72.3–99.7)	7.13 (1.46–13.0)
Sri Lanka	245,000 (189,000–315,000)	1370 (1070–1750)	338,000 (264,000–432,000)	1290 (1020–1650)	38.1 (29.5–48.1)	−5.80 (−10.8 to −0.509)
Thailand	1,160,000 (958,000–1,410,000)	1900 (1580–2290)	1,720,000 (1,430,000–2,080,000)	1720 (1430–2070)	48.2 (36.9–60.1)	−9.36 (−13.6 to −4.87)
Timor-Leste	7890 (6370–9790)	1320 (1070–1630)	13,200 (10,900–16,000)	1310 (1080–1590)	67.6 (58.0–76.9)	−0.507 (−5.33 to 4.87)
Viet Nam	907,000 (707,000–1,160,000)	1420 (1100–1780)	1,920,000 (1,540,000–2,370,000)	1670 (1360–2050)	111 (94.2–132)	18.2 (8.17–27.1)
**Sub-Saharan Africa**	**2,980,000 (2,380,000**–**3,720,000)**	**664 (536**–**829)**	**5,480,000 (4,390,000**–**6,800,000)**	**657 (538**–**815)**	**83.6 (81.3**–**86.6)**	**−0.995 (−2.60 to 0.405)**
Central Sub-Saharan Africa	305,000 (245,000–381,000)	618 (503–772)	646,000 (521,000–801,000)	667 (543–830)	112 (104–119)	7.94 (4.79–11.1)
Angola	62,400 (50,300–77,700)	651 (533–806)	143,000 (115,000–178,000)	662 (539–828)	128 (119–139)	1.81 (−2.13 to 6.41)
Central African Republic	15,900 (12,900–19700)	658 (539–819)	28,000 (23,100–34,100)	710 (584–869)	76.0 (66.2–85.8)	7.85 (1.84–13.5)
Congo	14,100 (11,300–17,800)	631 (511–782)	30,600 (24,600–37,600)	681 (553–843)	116 (102–129)	7.82 (3.41–12.2)
Democratic Republic of the Congo	204,000 (164,000–255,000)	605 (491–758)	427,000 (344,000–530,000)	663 (538–824)	109 (99.5–119)	9.72 (5.05–14.4)
Equatorial Guinea	2500 (2000–3080)	599 (484–740)	7080 (5670–8810)	661 (542–809)	184 (170–196)	10.4 (5.70–15.4)
Gabon	5990 (4850–7450)	659 (532–832)	11,000 (8900–13,400)	729 (596–896)	83.2 (74.6–92.6)	10.7 (6.08–15.4)
Eastern Sub-Saharan Africa	1,190,000 (951,000–1,500,000)	727 (586–929)	2,190,000 (1,760,000–2,720,000)	709 (580–886)	84.7 (81.3–88.4)	−2.49 (−4.86 to −0.214)
Burundi	30,000 (24,300–37,000)	701 (566–893)	63,900 (51,400–80,200)	690 (558–864)	113 (102–123)	−1.61 (−6.85 to 3.61)
Comoros	2830 (2270–3530)	703 (561–894)	4900 (3970–6090)	719 (584–898)	73.2 (63.1–83.1)	2.37 (−2.53 to 7.59)
Djibouti	3310 (2620–4170)	698 (565–879)	8400 (6760–10,600)	726 (589–911)	154 (137–170)	4.00 (−0.872 to 8.58)
Eritrea	19,200 (15,400–24,200)	704 (566–884)	37,800 (30,500–47,400)	720 (583–900)	97.2 (88.2–107)	2.30 (−2.45 to 6.70)
Ethiopia	354,000 (282,000–440,000)	792 (635–1010)	560,000 (450,000–698,000)	696 (568–865)	58.5 (53.4–64.3)	−12.1 (−15.2 to 8.65)
Kenya	153,000 (122,000–193,000)	746 (602–948)	296,000 (237,000–368,000)	740 (602–927)	92.7 (86.6–99.1)	−0.797 (−3.03 to 1.42)
Madagascar	74,500 (59,700–93,600)	695 (558–891)	151,000 (122,000–188,000)	707 (580–889)	103 (92.1–112)	1.73 (−3.67 to 6.60)
Malawi	50,400 (40,200–63,300)	693 (554–891)	95,900 (76,600–119,000)	701 (570–875)	90.0 (82.7–99.1)	1.20 (−2.34 to 5.54)
Mozambique	82,000 (65,400–103,000)	694 (557–893)	149,000 (120,000–184,000)	722 (592–902)	81.4 (72.3–90.4)	4.07 (−1.23 to 9.75)
Rwanda	37,600 (30,000–46,600)	700 (561–896)	73,800 (59,200–91,500)	698 (564–867)	96.4 (88.0–106)	−0.268 (−5.17 to 3.89)
Somalia	48,100 (38,500–60,300)	716 (579–901)	102,000 (83,100–126,000)	739 (608–908)	113 (99.8–127)	3.10 (−2.11 to 9.35)
South Sudan	32,500 (25,900–40,800)	672 (541–844)	47,000 (38,000–58,000)	673 (551–845)	44.5 (34.6–54.3)	0.0577 (−4.56 to 4.74)
Uganda	97,000 (77,800–120,000)	678 (546–861)	195,000 (158,000–244,000)	691 (559–861)	101 (92.5–110)	1.93 (−2.18 to 7.09)
United Republic of Tanzania	156,000 (125,000–197,000)	684 (547–882)	301,000 (243,000–371,000)	700 (573–882)	92.5 (81.4–101)	2.38 (−3.78 to 7.17)
Zambia	44,600 (35,500–56100)	726 (585–933)	103,000 (84,200–129,000)	748 (615–931)	132 (122–146)	2.96 (−2.20 to 9.10)
Southern Sub-Saharan Africa	383,000 (307,000–480,000)	710 (576–882)	534,000 (428,000–669,000)	680 (547–849)	39.3 (35.0–43.8)	−4.20 (−5.86 to −2.56)
Botswana	8850 (7320–10,900)	678 (563–838)	16,000 (12,800–20,500)	686 (551–862)	81.2 (67.4–94.3)	1.20 (−5.62 to 6.81)
Eswatini	4800 (3810–6000)	664 (534–829)	6600 (5330–8280)	676 (548–844)	37.4 (30.5–45.9)	1.79 (−2.19 to 6.82)
Lesotho	8970 (7190–11,100)	637 (513–792)	10,900 (8810–13,600)	667 (547–823)	21.6 (13.9–28.9)	4.83 (0.148–9.97)
Namibia	9170 (7270–11,500)	660 (535–817)	14,300 (11,400–18,100)	663 (532–831)	55.8 (48.6–62.9)	0.455 (−2.98 to 4.14)
South Africa	292,000 (233,000–365,000)	722 (584–897)	401,000 (322,000–504,000)	682 (548–854)	37.6 (32.9–42.6)	−5.42 (−7.27 to −3.74)
Zimbabwe	59,600 (47,000–74,900)	685 (554–850)	84,400 (67,300–104,000)	670 (539–823)	41.8 (34.7–48.3)	−2.16 (−5.70 to 1.43)
Western Sub-Saharan Africa	1,110,000 (884,000–1390,000)	609 (492–765)	2,110,000 (1,680,000–2,630,000)	606 (493–755)	90.1 (87.4–93.0)	−0.632 (−2.18 to 1.02)
Benin	24,800 (19,700–31,400)	565 (456–714)	54,200 (43,200–68,200)	579 (469–731)	119 (111–127)	2.51 (−1.32 to 6.51)
Burkina Faso	46,800 (37,500–58,700)	576 (464–738)	92,700 (73,900–116,000)	591 (479–744)	97.9 (89.7–106)	2.47 (−2.00 to 6.94)
Cabo Verde	1830 (1440–2310)	561 (449–704)	3400 (2680–4330)	593 (474–749)	85.7 (70.1–100)	5.65 (1.30–9.90)
Cameroon	60,000 (47,400–75,900)	578 (468–725)	137,000 (109,000–171,000)	587 (474–727)	128 (119–138)	1.60 (−2.32 to 5.50)
Chad	30,000 (24,000–37,200)	564 (459–709)	62,800 (49,400–78,700)	576 (461–718)	109 (101–118)	2.06 (−1.90 to 6.23)
Côte d'Ivoire	69,200 (55,100–87,100)	578 (463–730)	126,000 (99,600–158,000)	592 (476–743)	81.3 (72.4–90.1)	2.54 (−1.32 to 7.24)
Gambia	5330 (4270–6690)	578 (466–727)	10,200 (8120–12,700)	586 (473–728)	90.7 (82.9–100)	1.42 (−2.50 to 5.86)
Ghana	92,800 (75,100–116,000)	678 (555–834)	210,000 (174,000–255,000)	842 (705–1010)	127 (115–142)	24.3 (16.3–34.5)
Guinea	32,100 (25,800–40,000)	566 (457–713)	53,800 (43,000–67,300)	575 (465–716)	67.6 (62.0–75.6)	1.49 (−2.06 to 6.02)
Guinea-Bissau	4780 (3820–5920)	577 (469–726)	8530 (6750–10,700)	579 (468–720)	78.6 (71.5–86.5)	0.372 (−3.82 to 4.34)
Liberia	11,300 (9060–14,400)	577 (462–731)	25,000 (19,800–31,300)	591 (474–737)	120 (110–131)	2.53 (−1.68 to 6.77)
Mali	43,400 (34,900–54,200)	579 (469–720)	91,500 (73,000–115,000)	585 (470–734)	111 (102–119)	0.908 (−2.80 to 4.69)
Mauritania	10,900 (8720–137,00)	585 (473–733)	19,300 (15,400–24,000)	595 (479–747)	77.1 (69.7–84.4)	1.62 (−2.59 to 5.80)
Niger	41,900 (33,500–52,900)	574 (457–724)	87,200 (69,300–108,000)	573 (461–712)	108 (98.1–119)	−0.0983 (−3.70 to 4.78)
Nigeria	555,000 (441,000–700,000)	630 (506–791)	977,000 (774,000–1,220,000)	583 (471–730)	75.9 (72.9–79.0)	−7.60 (−8.96 to −6.16)
Sao Tome and Principe	562 (450–698)	581 (468–734)	1100 (886–1380)	606 (493–765)	96.3 (84.1–108)	4.32 (0.290–8.44)
Senegal	40,000 (31,700–49,800)	573 (459–723)	70,600 (56,200–88,400)	580 (470–734)	76.8 (69.6–85.1)	1.36 (−2.93 to 5.90)
Sierra Leone	18,100 (14,400–23,100)	566 (455–731)	38,500 (30,600–48,800)	580 (466–729)	113 (105–122)	2.59 (−0.897 to 6.36)
Togo	19,300 (15,200–24,300)	567 (456–719)	38,800 (31,200–49,200)	577 (471–729)	101 (91.8–113)	1.83 (−2.29 to 6.75)

**Table 2:** Global, super-region, and country-level DALYs of acute urolithiasis for both sexes and all locations, and percentage change in 2000 and 2021.

location_name	count.2000	rate.2000	count.2021	rate.2021	Percentage change in counts between 2000 and 2021	Percentage change in age-standardised rates between 2000 and 2021
**Global**	**515,000 (411,000–631,000)**	**9.59 (7.65–11.6)**	**693,000 (568,000–850,000)**	**8.15 (6.68–9.99)**	**34.5 (24.7–47.3)**	**−15.0 (−21.3 to 6.93)**
**Central Europe, Eastern Europe, and Central Asia**	**84,900 (72,100–101,000)**	**17.0 (14.3–20.3)**	**91,000 (77,700–111,000)**	**15.3 (13.0–18.9)**	**7.13 (0.539–19.9)**	**−9.73 (−14.9 to 0.348)**
Central Asia	7230 (5780–8910)	12.3 (9.84–15.3)	11,000 (8930–13,900)	12.9 (10.5–16.1)	52.4 (26.8–84.2)	4.66 (−14.5 to 27.8)
Armenia	614 (485–840)	18.7 (14.9–25.6)	725 (591–894)	17.8 (14.5–22.0)	18.2 (−7.28 to 50.5)	−4.97 (−25.4 to 21.1)
Azerbaijan	462 (334–626)	6.52 (4.83–8.81)	704 (482–1010)	6.49 (4.53–9.28)	52.3 (20.3–101)	−0.561 (−22.8 to 34.2)
Georgia	311 (240–406)	5.62 (4.27–7.41)	277 (224–350)	5.61 (4.50–7.16)	−11.1 (−27.8 to 10.6)	−0.139 (−18.2 to 22.2)
Kazakhstan	3680 (2730–4710)	27.1 (20.2–34.7)	5980 (4740–7690)	33.3 (26.4–43.0)	62.3 (14.9–126)	23.1 (−13.5 to 72.7)
Kyrgyzstan	382 (301–482)	9.90 (7.90–12.6)	502 (391–654)	8.62 (6.82–11.1)	31.2 (12.2–55.2)	−12.9 (−26.4 to 2.89)
Mongolia	141 (101–189)	8.46 (6.07–11.9)	179 (127–252)	6.07 (4.34–8.46)	27.5 (−4.15 to 66.2)	−28.3 (−48.4 to −4.43)
Tajikistan	566 (413–770)	14.4 (10.5–20.5)	862 (610–1220)	12.1 (8.57–17.0)	52.2 (5.29–120)	−16.0 (−43.6 to 21.8)
Turkmenistan	199 (140–266)	6.23 (4.49–8.20)	337 (237–450)	7.19 (5.06–9.56)	69.2 (33.3–113)	15.4 (−11.9 to 48.3)
Uzbekistan	869 (552–1320)	4.30 (2.68–6.46)	1450 (921–2150)	4.26 (2.71–6.29)	67.3 (36.9–104)	−0.929 (−17.2 to 20.1)
Central Europe	10,600 (8690–12,900)	6.89 (5.59–8.58)	7540 (5930–9650)	4.42 (3.40–5.85)	−28.8 (−35.0 to 20.3)	−35.8 (−41.5 to 28.9)
Albania	139 (100–190)	4.66 (3.36–6.34)	138 (95.4–197)	3.95 (2.73–5.60)	−0.317 (−19.6 to 22.2)	−15.3 (−30.1 to 0.118)
Bosnia and Herzegovina	329 (239–450)	6.74 (4.97–9.06)	230 (156–354)	4.69 (3.24–6.75)	−30.2 (−54.8 to −3.37)	−30.3 (−52.7 to 8.09)
Bulgaria	1040 (856–1270)	9.05 (7.48–11.2)	607 (456–813)	5.72 (4.29–7.67)	−41.4 (−54.2 to 25.2)	−36.8 (−49.0 to 20.3)
Croatia	361 (278–467)	5.88 (4.50–7.61)	263 (195–353)	4.19 (3.03–5.74)	−27.1 (−41.4 to 12.4)	−28.8 (−42.0 to 14.5)
Czechia	1110 (912–1360)	8.04 (6.51–9.87)	839 (647–1090)	5.17 (3.90–6.94)	−24.7 (−37.3 to 9.97)	−35.7 (−46.3 to 23.1)
Hungary	2050 (1770–2380)	14.0 (12.1–16.5)	1510 (1230–1910)	9.16 (7.32–11.7)	−26.2 (−37.9 to 10.2)	−34.7 (−44.9 to 20.6)
Montenegro	27.6 (19.5–38.7)	3.84 (2.68–5.43)	27.4 (18.8–38.6)	3.50 (2.43–4.99)	−0.656 (−16.5 to 18.5)	−8.93 (−21.5 to 5.96)
North Macedonia	84.8 (58.5–120)	3.74 (2.59–5.29)	93.3 (60.7–138)	3.32 (2.18–4.95)	10.1 (−7.50 to 32.4)	−11.2 (−23.8 to 4.25)
Poland	3150 (2480–4000)	6.83 (5.35–8.78)	1960 (1550–2560)	3.54 (2.76–4.73)	−37.8 (−45.1 to 26.2)	−48.2 (−53.7 to 39.2)
Romania	899 (597–1280)	3.37 (2.24–4.75)	769 (477–1130)	3.15 (2.01–4.77)	−14.4 (−29.6 to 3.86)	−6.59 (−20.7 to 10.6)
Serbia	589 (450–779)	4.92 (3.74–6.56)	519 (370–716)	4.13 (2.92–5.88)	−11.9 (−26.1 to 5.46)	−16.1 (−29.2 to −1.25)
Slovakia	522 (395–695)	8.18 (6.25–11.0)	364 (253–520)	4.81 (3.32–6.83)	−30.3 (−45.4 to 11.0)	−41.2 (−53.9 to 24.9)
Slovenia	135 (102–174)	5.19 (3.86–6.80)	114 (83.5–161)	3.74 (2.63–5.29)	−15.4 (−31.2 to 7.30)	−27.9 (−40.2 to 12.2)
Eastern Europe	67,100 (57,200–79,300)	23.6 (20.1–28.1)	72,400 (62,100–87,900)	22.8 (19.4–28.0)	7.91 (0.348–21.5)	−3.29 (−9.59 to 8.06)
Belarus	3260 (2660–4020)	24.5 (19.9–30.3)	3760 (3030–4490)	25.4 (20.3–30.7)	15.4 (−8.96 to 42.9)	4.03 (−16.9 to 27.6)
Estonia	360 (299–438)	18.6 (15.3–22.9)	374 (303–469)	17.3 (13.8–22.1)	4.01 (−10.7 to 23.6)	−7.00 (−18.6 to 8.63)
Latvia	888 (754–1040)	26.2 (22.1–31.3)	798 (645–1000)	24.1 (19.4–30.6)	−10.2 (−24.2 to 8.27)	−8.00 (−21.9 to 10.5)
Lithuania	1030 (872–1240)	22.3 (18.7–27.1)	997 (794–1290)	21.3 (16.9–28.0)	−3.53 (−19.7 to 21.7)	−4.71 (−20.0 to 19.7)
Republic of Moldova	838 (686–1030)	17.5 (14.3–21.6)	1000 (813–1240)	18.4 (14.9–23.0)	19.6 (2.77–41.9)	5.42 (−8.43 to 23.7)
Russian Federation	48,800 (42,500–56,900)	25.7 (22.2–30.1)	56,100 (48,700–67,400)	25.6 (22.1–31.1)	15.0 (5.28–32.3)	−0.333 (−7.95 to 13.4)
Ukraine	11,900 (9380–15100)	18.2 (14.4–23.2)	9370 (6950–12,500)	14.6 (10.9–19.6)	−21.5 (−33.5 to 8.24)	−19.8 (−31.9 to −7.65)
**High-income**	**69,500 (54,500**–**88,100)**	**5.53 (4.27**–**7.15)**	**103,000 (85,300**–**124,000)**	**5.89 (4.73**–**7.34)**	**47.8 (37.7**–**60.8)**	**6.52 (1.88**–**12.9)**
Australasia	1630 (1280–2090)	5.84 (4.53–7.57)	2770 (2230–3450)	6.15 (4.82–7.97)	69.9 (52.9–89.1)	5.34 (−5.66 to 16.6)
Australia	1290 (990–1700)	5.56 (4.21–7.35)	2210 (1770–2790)	5.86 (4.52–7.63)	71.0 (51.4–94.0)	5.39 (−7.24 to 19.3)
New Zealand	338 (280–412)	7.25 (5.97–8.95)	560 (465–671)	7.65 (6.28–9.40)	65.7 (48.6–84.5)	5.57 (−4.63 to 16.3)
High-income Asia Pacific	13,100 (9890–17,400)	5.45 (4.06–7.33)	23,600 (19,500–28,500)	6.29 (4.96–7.98)	79.9 (54.9–113)	15.5 (6.26–28.0)
Brunei Darussalam	16.6 (12.1–22.7)	7.80 (5.82–10.5)	32.3 (24.2–43.8)	8.07 (6.02–10.7)	95.0 (54.4–146)	3.41 (−17.8 to 35.4)
Japan	10,500 (7940–13,900)	5.58 (4.18–7.50)	19,400 (16,300–22,800)	6.80 (5.46–8.56)	85.5 (55.6–120)	21.8 (10.3–35.5)
Republic of Korea	2420 (1680–3470)	5.03 (3.59–6.95)	3720 (2610–5150)	4.68 (3.29–6.58)	53.7 (19.3–93.3)	−7.00 (−27.1 to 13.9)
Singapore	233 (166–321)	5.74 (4.25–7.74)	468 (354–633)	5.69 (4.31–7.58)	101 (68.0–143)	−0.938 (−15.4 to 15.1)
High-income North America	19,100 (15,200–24,100)	5.06 (3.97–6.45)	31,600 (27,200–36,900)	5.67 (4.80–6.72)	65.3 (50.4–84.4)	12.1 (3.49–23.6)
Canada	1670 (1310–2130)	4.36 (3.37–5.59)	3320 (2710–4100)	5.48 (4.41–6.91)	98.9 (73.2–132)	25.7 (11.8–44.7)
Greenland	1.81 (1.24–2.62)	3.37 (2.36–4.74)	2.10 (1.42–2.98)	3.18 (2.20–4.39)	15.7 (−6.16 to 42.4)	−5.88 (−20.4 to 13.3)
United States of America	17,400 (13,900–22,000)	5.14 (4.04–6.56)	28,200 (24,400–33,100)	5.69 (4.83–6.75)	62.1 (46.6–81.5)	10.8 (1.80–22.6)
Southern Latin America	3480 (2570–4670)	6.31 (4.66–8.50)	5100 (3670–6970)	6.56 (4.68–9.07)	46.9 (30.6–65.2)	3.99 (−7.59 to 17.5)
Argentina	2090 (1440–2950)	5.77 (3.95–8.22)	2690 (1800–3870)	5.31 (3.55–7.68)	29.2 (10.5–52.6)	−7.93 (−21.4 to 9.45)
Chile	1120 (847–1460)	7.37 (5.64–9.50)	2100 (1550–2760)	9.19 (6.73–12.3)	87.0 (57.3–125)	24.7 (4.26–49.8)
Uruguay	268 (205–352)	7.37 (5.56–9.86)	314 (243–404)	7.32 (5.52–9.61)	17.1 (2.11–35.9)	−0.632 (−14.0 to 15.8)
Western Europe	32,100 (25,700–40,100)	5.77 (4.51–7.43)	39,600 (31,700–49,200)	5.63 (4.37–7.25)	23.4 (16.6 to 32.9)	−2.38 (−6.71 to 3.53)
Andorra	5.19 (3.70–7.24)	6.02 (4.30–8.41)	7.46 (5.19–10.2)	5.45 (3.77–7.46)	43.9 (15.1–83.0)	−9.47 (−27.7 to 13.5)
Austria	1230 (954–1590)	11.1 (8.48–14.6)	1200 (975–1490)	8.18 (6.42–10.6)	−1.85 (−17.8 to 15.6)	−26.3 (−38.0 to 13.3)
Belgium	983 (743–1300)	6.99 (5.17–9.43)	952 (744–1210)	5.37 (4.02–7.12)	−3.15 (−18.0 to 12.6)	−23.2 (−35.1 to 9.76)
Cyprus	69.2 (51.0–95.6)	7.07 (5.21–9.85)	98.0 (71.6–136)	5.46 (3.96–7.68)	41.6 (6.03–88.0)	−22.7 (−42.3 to 3.61)
Denmark	581 (495–686)	7.63 (6.36–9.21)	679 (567–825)	6.78 (5.48–8.47)	16.8 (3.28–32.8)	−11.2 (−22.0 to 1.91)
Finland	256 (209–314)	3.44 (2.77–4.28)	392 (305–506)	4.39 (3.26–5.91)	53.1 (28.0–83.4)	27.8 (5.09–56.8)
France	3920 (2930–5220)	4.94 (3.61–6.76)	4520 (3470–5950)	4.52 (3.34–6.13)	15.4 (−0.523 to 33.2)	−8.52 (−20.9 to 5.70)
Germany	5220 (3960–6840)	4.40 (3.26–5.97)	6560 (5080–8390)	4.59 (3.44–6.08)	25.8 (7.71–47.7)	4.19 (−9.75 to 22.7)
Greece	557 (376–817)	3.88 (2.59–5.69)	650 (434–925)	4.45 (2.94–6.38)	16.7 (−4.50 to 43.5)	14.6 (−6.48 to 41.8)
Iceland	15.7 (12.0–20.3)	4.87 (3.67–6.41)	27.5 (22.0–34.7)	5.46 (4.23–7.13)	74.5 (50.7–103)	12.1 (−1.90 to 30.2)
Ireland	231 (177–302)	5.26 (4.00–6.91)	304 (226–408)	4.58 (3.34–6.25)	31.5 (13.7–52.8)	−13.0 (−25.4 to 0.414)
Israel	480 (402–596)	7.48 (6.20–9.30)	735 (595–922)	6.52 (5.14–8.38)	53.1 (34.8–73.1)	−12.8 (−23.6 to −1.05)
Italy	5940 (4810–7390)	6.98 (5.50–8.94)	5800 (4570–7380)	5.55 (4.23–7.40)	−2.39 (−10.3 to 7.63)	−20.5 (−26.9 to 12.7)
Luxembourg	38.7 (27.8–53.7)	7.01 (5.00–9.71)	42.1 (30.2–58.0)	4.65 (3.26–6.48)	8.73 (−8.92 to 29.3)	−33.7 (−45.0 to 21.6)
Malta	35.4 (27.5–46.1)	7.01 (5.40–9.12)	42.4 (33.9–52.9)	5.81 (4.43–7.53)	19.7 (4.08–40.4)	−17.0 (−27.7 to −3.76)
Monaco	1.89 (1.27–2.72)	3.82 (2.56–5.62)	2.08 (1.40–3.07)	3.61 (2.39–5.36)	10.4 (−8.31 to 33.5)	−5.70 (−21.3 to 13.7)
Netherlands	1680 (1390–2020)	7.75 (6.33–9.42)	2020 (1680–2460)	6.96 (5.60–8.88)	20.2 (7.35–36.9)	−10.2 (−20.4 to 2.42)
Norway	541 (421–690)	8.81 (6.63–11.6)	701 (558–874)	8.53 (6.52–11.1)	29.6 (22.1–39.2)	−3.17 (−8.71 to 3.40)
Portugal	646 (497–851)	4.60 (3.47–6.14)	800 (633–1000)	4.50 (3.44–5.86)	23.9 (4.63–47.9)	−2.12 (−17.5 to 16.0)
San Marino	2.31 (1.72–3.16)	5.59 (4.08–7.70)	2.69 (1.91–3.72)	4.85 (3.53–6.81)	16.2 (−7.22 to 44.8)	−13.3 (−29.0 to 7.11)
Spain	2600 (1990–3390)	4.63 (3.46–6.21)	3640 (2790–4730)	5.06 (3.69–6.82)	40.2 (18.3–64.8)	9.29 (−9.81 to 28.9)
Sweden	618 (485–789)	4.64 (3.52–6.12)	958 (767–1210)	5.47 (4.17–7.21)	55.0 (37.0–77.6)	18.0 (4.31–33.8)
Switzerland	673 (507–873)	6.83 (5.03–8.95)	617 (455–842)	4.56 (3.22–6.36)	−8.25 (−26.1 to 13.5)	−33.2 (−46.7 to 16.4)
United Kingdom	5790 (4870–6950)	7.06 (5.81–8.68)	8840 (7390–10,700)	8.42 (6.89–10.5)	52.6 (46.1–61.4)	19.3 (14.6–25.4)
**Latin America and Caribbean**	**27,900 (24,300**–**33,000)**	**7.53 (6.64**–**8.76)**	**59,300 (52,300**–**69,800)**	**9.31 (8.23**–**10.9)**	**113 (103**–**124)**	**23.7 (18.6**–**29.7)**
Andean Latin America	2290 (1710–3100)	6.22 (4.72–8.42)	4280 (3170–5850)	6.57 (4.91–8.96)	86.9 (62.3–117)	5.61 (−8.04 to 22.5)
Bolivia (Plurinational State of)	450 (309–624)	7.55 (5.17–10.3)	783 (534–1110)	7.33 (4.93–10.3)	74.0 (36.6–120)	−2.89 (−23.5 to 24.2)
Ecuador	679 (512–892)	6.88 (5.22–8.99)	1440 (1060–2050)	8.25 (6.10–11.7)	113 (71.0–182)	20.0 (−3.10 to 59.2)
Peru	1160 (837–1680)	5.53 (3.95–7.90)	2060 (1420–2890)	5.57 (3.88–7.80)	76.9 (44.6–114)	0.692 (−17.3 to 22.0)
Caribbean	2680 (2280–3240)	7.52 (6.42–9.03)	4720 (3940–5740)	8.92 (7.40–10.8)	76.0 (58.0–96.2)	18.5 (6.36–32.0)
Antigua and Barbuda	3.51 (2.77–4.52)	5.09 (4.03–6.48)	6.18 (4.90–7.86)	5.60 (4.44–7.08)	76.2 (52.6–101)	9.91 (−3.93 to 23.7)
Bahamas	17.6 (14.3–21.8)	6.62 (5.47–8.19)	32.6 (26.0–41.4)	7.36 (5.90–9.26)	85.8 (55.7–120)	11.2 (−6.83 to 31.9)
Barbados	26.2 (22.5–31.0)	8.83 (7.52–10.4)	46.2 (36.7–58.0)	10.0 (7.97–12.6)	76.1 (40.8–114)	13.4 (−8.36 to 37.8)
Belize	12.6 (10.7–15.0)	8.49 (7.30–10.0)	32.6 (27.1–39.5)	9.05 (7.60–10.8)	158 (125–194)	6.63 (−7.48 to 22.5)
Bermuda	4.01 (3.19–5.15)	5.09 (4.09–6.54)	5.36 (4.23–6.95)	5.13 (3.90–6.74)	33.7 (14.4–58.4)	0.748 (−13.2 to 16.8)
Cuba	1030 (882–1240)	8.05 (6.87–9.69)	1780 (1470–2160)	10.1 (8.33–12.3)	72.7 (49.8–98.0)	25.2 (8.47–43.1)
Dominica	2.23 (1.54–3.12)	3.53 (2.42–4.88)	3.22 (2.29–4.46)	3.98 (2.84–5.47)	44.6 (18.5–76.0)	12.5 (−7.09 to 36.5)
Dominican Republic	266 (187–368)	3.98 (2.82–5.43)	439 (323–611)	4.06 (3.02–5.58)	65.2 (39.6–98.5)	2.06 (−13.7 to 23.8)
Grenada	7.35 (6.04–9.12)	8.88 (7.30–10.9)	15.0 (12.3–18.5)	12.5 (10.3–15.4)	104 (75.9–138)	41.1 (22.5–63.3)
Guyana	66.9 (57.6–77.7)	12.5 (10.9–14.5)	104 (81.3–134)	14.4 (11.3–18.5)	55.1 (20.7–98.1)	15.0 (−11.1 to 46.4)
Haiti	407 (256–575)	7.59 (4.79–10.8)	769 (477–1130)	8.01 (4.82–11.8)	88.7 (40.2–149)	5.45 (−21.7 to 44.4)
Jamaica	186 (160–220)	8.54 (7.36–10.1)	326 (256–416)	10.5 (8.26–13.5)	74.7 (37.6–119)	23.4 (−3.47 to 53.2)
Puerto Rico	215 (171–278)	4.94 (3.90–6.41)	316 (255–398)	6.21 (4.99–7.97)	46.5 (22.5–73.8)	25.7 (6.29–46.3)
Saint Kitts and Nevis	1.67 (1.22–2.25)	4.12 (3.02–5.56)	3.61 (2.67–4.88)	4.72 (3.50–6.28)	117 (81.4–159)	14.6 (−1.40 to 36.7)
Saint Lucia	9.13 (7.69–11.3)	7.31 (6.18–8.90)	19.6 (15.9–24.3)	8.43 (6.81–10.4)	114 (78.4–155)	15.3 (−2.80 to 36.1)
Saint Vincent and the Grenadines	5.74 (4.69–7.17)	6.55 (5.36–8.13)	11.5 (9.32–14.3)	8.19 (6.67–10.2)	99.7 (71.3–129)	25.0 (7.80–42.7)
Suriname	34.5 (23.7–46.1)	9.40 (6.32–12.6)	70.2 (46.0–96.0)	10.7 (6.91–14.7)	104 (53.6–173)	13.3 (−15.1 to 50.5)
Trinidad and Tobago	277 (250–306)	23.8 (21.5–26.3)	567 (448–723)	30.1 (23.9–38.4)	105 (59.1–162)	26.4 (−1.34 to 61.8)
United States Virgin Islands	16.1 (12.6–20.9)	12.9 (10.1–16.7)	16.6 (12.2–23.0)	11.4 (8.42–15.4)	3.46 (−21.8 to 32.8)	−11.9 (−32.9 to 11.5)
Central Latin America	12,500 (11,200–14,400)	8.71 (7.87–9.89)	23,500 (20,200–28,300)	8.99 (7.76–10.8)	87.6 (72.0–106)	3.19 (−5.34 to 12.8)
Colombia	1460 (1150–1880)	4.46 (3.54–5.69)	2290 (1800–2960)	4.21 (3.31–5.43)	56.6 (36.1–83.9)	−5.65 (−18.6 to 10.7)
Costa Rica	103 (73.6–140)	3.15 (2.24–4.37)	160 (118–219)	2.97 (2.21–4.05)	55.5 (33.2–84.4)	−5.54 (−19.0 to 11.4)
El Salvador	169 (126–225)	3.88 (2.87–5.11)	232 (173–317)	3.75 (2.80–5.13)	37.2 (16.0–65.7)	−3.25 (−18.3 to 17.2)
Guatemala	490 (424–572)	7.59 (6.57–8.79)	830 (683–1010)	6.50 (5.38–7.85)	69.5 (46.3–95.6)	−14.3 (−27.0 to −0.532)
Honduras	545 (299–797)	14.9 (7.85–22.2)	1060 (578–1580)	14.6 (7.78–21.9)	93.9 (35.3–177)	−1.51 (−32.7 to 40.7)
Mexico	7850 (7150–8790)	11.0 (10.1–12.2)	15,600 (13,200–19,100)	11.6 (9.87–14.1)	99.0 (78.2–129)	5.43 (−5.56 to 21.1)
Nicaragua	136 (104–178)	4.30 (3.23–5.62)	246 (183–327)	4.22 (3.15–5.59)	81.3 (52.4–127)	−1.74 (−18.0 to 24.4)
Panama	94.1 (70.9–124)	3.81 (2.89–4.95)	163 (124–213)	3.69 (2.81–4.84)	72.8 (47.0–104)	−3.00 (−17.4 to 14.3)
Venezuela (Bolivarian Republic of)	1690 (1480–1950)	9.79 (8.67–11.1)	2940 (2290–3670)	9.78 (7.67–12.2)	73.4 (38.6–117)	−0.108 (−19.9 to 24.7)
Tropical Latin America	10,400 (9010–12,200)	6.72 (5.90–7.83)	26,800 (24,100–30,200)	10.4 (9.35–11.7)	159 (142–176)	54.5 (45.6–64.1)
Brazil	10,200 (8890–12,000)	6.79 (5.97–7.90)	26,400 (23,800–29,700)	10.5 (9.47–11.8)	159 (142–176)	54.6 (45.5–64.0)
Paraguay	153 (114–208)	4.04 (3.02–5.42)	399 (231–560)	6.18 (3.52–8.72)	161 (62.4–271)	53.1 (−7.22 to 115)
**North Africa and Middle East**	**13,800 (10,200**–**18,000)**	**4.38 (3.19**–**5.67)**	**25,200 (17,900**–**33,800)**	**4.55 (3.18**–**5.98)**	**83.2 (59.8**–**107)**	**3.73 (−11.2 to 18.4)**
North Africa and Middle East	13,800 (10,200–18,000)	4.38 (3.19–5.67)	25,200 (17,900–33,800)	4.55 (3.18–5.98)	83.2 (59.8–107)	3.73 (−11.2 to 18.4)
Afghanistan	423 (252–602)	4.18 (2.70–5.98)	1180 (579–1920)	6.51 (2.88–11.1)	178 (56.1–304)	56.0 (−17.4 to 131)
Algeria	731 (514–1060)	3.03 (2.13–4.34)	1610 (967–2340)	3.90 (2.25–5.65)	120 (53.1–199)	28.8 (−15.0 to 74.7)
Bahrain	19.1 (12.3–28.8)	3.72 (2.53–5.65)	68.7 (43.8–102)	5.25 (3.03–8.16)	260 (140–417)	40.9 (−24.4 to 148)
Egypt	1830 (1290–2520)	3.68 (2.62–5.03)	4080 (2920–5400)	5.38 (3.78–7.07)	123 (58.7–200)	46.2 (−4.48 to 107)
Iran (Islamic Republic of)	4190 (2410–5400)	8.64 (4.89–11.2)	5200 (3810–6570)	6.03 (4.26–7.52)	24.1 (−0.941 to 74.7)	−30.2 (−43.8 to −0.975)
Iraq	728 (510–975)	4.35 (3.02–5.83)	1340 (962–1870)	4.12 (2.90–5.59)	84.2 (46.6–129)	−5.22 (−26.1 to 23.2)
Jordan	87.3 (56.7–132)	2.53 (1.63–3.87)	375 (241–556)	3.28 (2.16–4.89)	329 (237–443)	29.9 (0.549–63.2)
Kuwait	49.3 (30.0–76.9)	2.62 (1.62–3.91)	167 (112–234)	3.15 (2.22–4.34)	238 (161–347)	20.0 (−2.88 to 60.1)
Lebanon	136 (97.5–182)	4.28 (3.07–5.70)	240 (178–329)	3.96 (2.93–5.43)	76.4 (37.3–127)	−7.57 (−28.5 to 19.4)
Libya	124 (89.0–170)	3.32 (2.42–4.55)	369 (198–530)	5.43 (2.74–7.85)	197 (79.9–336)	63.2 (−7.66 to 146)
Morocco	747 (529–1040)	3.05 (2.15–4.25)	1660 (938–2380)	4.45 (2.42–6.39)	122 (39.8–208)	45.9 (−10.2 to 104)
Oman	51.8 (33.3–76.7)	2.99 (1.98–4.22)	154 (101–240)	3.41 (2.32–5.01)	197 (129–282)	14.2 (−12.6 to 48.9)
Palestine	56.7 (39.4–80.0)	3.13 (2.18–4.44)	114 (77.6–165)	2.95 (2.08–4.25)	102 (63.5–143)	−6.00 (−26.5 to 16.8)
Qatar	24.3 (14.8–34.7)	5.57 (3.11–8.00)	118 (71.1–187)	3.92 (2.57–5.92)	385 (239–632)	−29.7 (−52.6 to 32.1)
Saudi Arabia	436 (273–653)	2.54 (1.61–3.75)	1180 (735–1820)	2.73 (1.79–4.08)	170 (106–255)	7.43 (−16.9 to 36.9)
Sudan	672 (452–956)	3.46 (2.46–4.74)	1390 (779–2000)	4.58 (2.38–6.65)	107 (35.2–198)	32.4 (−19.0 to 88.5)
Syrian Arab Republic	483 (347–651)	4.65 (3.34–6.12)	573 (408–796)	4.02 (2.86–5.62)	18.6 (−12.3 to 59.9)	−13.4 (−35.9 to 15.4)
Tunisia	248 (171–347)	2.87 (1.99–4.02)	469 (282–678)	3.50 (2.08–5.04)	89.1 (32.0–156)	22.2 (−15.1 to 66.5)
Türkiye	2260 (1650–3100)	3.87 (2.81–5.33)	3550 (2540–4940)	3.79 (2.72–5.27)	57.1 (27.0–99.5)	−2.19 (−21.4 to 23.8)
United Arab Emirates	105 (67.3–157)	4.44 (2.95–6.59)	454 (269–733)	4.60 (3.16–6.78)	332 (188–520)	3.61 (−34.3 to 53.4)
Yemen	353 (248–495)	3.08 (2.17–4.32)	905 (517–1380)	4.09 (2.17–6.67)	156 (74.8–246)	32.8 (−15.4 to 90.6)
**South Asia**	**100,000 (65,700**–**134,000)**	**10.8 (7.07**–**14.6)**	**166,000 (114,000**–**231,000)**	**10.1 (6.83**–**14.1)**	**65.8 (41.0**–**97.3)**	**−6.56 (−21.3 to 11.6)**
South Asia	100,000 (65,700–134,000)	10.8 (7.07–14.6)	166,000 (114,000–231,000)	10.1 (6.83–14.1)	65.8 (41.0–97.3)	−6.56 (−21.3 to 11.6)
Bangladesh	6430 (4360–9250)	8.45 (5.40–12.8)	10,200 (6750–15,500)	6.79 (4.43–10.5)	58.5 (23.8–105)	−19.6 (−40.3 to 5.84)
Bhutan	40.2 (25.5–68.7)	9.65 (5.99–16.7)	59.4 (35.8–93.4)	8.82 (5.13–14.3)	47.7 (10.2–103)	−8.61 (−33.1 to 24.3)
India	81,200 (52,700–108,000)	10.8 (6.98–14.5)	135,000 (90,200–190,000)	10.3 (6.78–14.7)	66.3 (39.3–101)	−4.45 (−20.9 to 15.2)
Nepal	1100 (749–1530)	7.04 (4.68–9.92)	2060 (1360–3080)	7.97 (5.19–12.0)	87.7 (47.3–138)	13.2 (−13.9 to 46.5)
Pakistan	11,500 (7180–16,600)	13.7 (8.50–20.4)	19,000 (12,100–28,000)	12.1 (7.65–17.7)	64.5 (23.3–114)	−12.0 (−33.7 to 14.8)
**Southeast Asia, East Asia, and Oceania**	**191,000 (126,000**–**237,000)**	**11.9 (7.67**–**14.6)**	**206,000 (148,000**–**255,000)**	**7.47 (5.33**–**9.22)**	**7.87 (−3.63 to 26.8)**	**−37.0 (−44.2 to 25.2)**
East Asia	133,000 (89,800–166,000)	11.2 (7.41–13.7)	117,000 (90,200–149,000)	5.72 (4.42–7.31)	−12.1 (−26.3 to 13.9)	−48.9 (−57.7 to 32.3)
China	130,000 (87,300–161,000)	11.3 (7.48–14.0)	111,000 (85,200–143,000)	5.63 (4.31–7.27)	−14.1 (−28.7 to 12.1)	−50.3 (−59.1 to 33.6)
Democratic People's Republic of Korea	2460 (1430–3460)	10.9 (6.27–15.3)	3130 (1990–4280)	9.33 (5.87–12.7)	26.9 (−2.92 to 67.2)	−14.7 (−34.1 to 12.7)
Taiwan (Province of China)	1140 (876–1560)	4.85 (3.76–6.61)	2720 (2170–3420)	7.13 (5.65–9.08)	138 (97.9–182)	47.0 (25.4–74.8)
Oceania	173 (115–248)	2.98 (2.01–4.25)	315 (209–461)	2.81 (1.91–4.04)	81.8 (56.8–106)	−5.71 (−18.6 to 7.10)
American Samoa	1.51 (1.03–2.18)	3.45 (2.36–5.00)	1.72 (1.16–2.45)	3.26 (2.23–4.62)	14.0 (−7.62 to 40.9)	−5.40 (−21.2 to 15.1)
Cook Islands	0.551 (0.368–0.803)	3.03 (2.03–4.40)	0.626 (0.410–0.917)	2.86 (1.87–4.16)	13.7 (−7.68 to 41.9)	−5.58 (−21.9 to 14.0)
Fiji	19.0 (12.4–28.2)	2.73 (1.75–4.07)	26.6 (17.4–38.7)	2.87 (1.89–4.12)	39.8 (13.1–75.0)	4.83 (−14.5 to 32.3)
Guam	4.66 (3.15–6.74)	3.14 (2.15–4.51)	5.18 (3.24–7.73)	2.77 (1.77–4.18)	11.3 (−11.5 to 41.6)	−11.8 (−29.2 to 9.96)
Kiribati	2.34 (1.52–3.30)	3.86 (2.46–5.38)	3.39 (2.34–4.73)	3.42 (2.38–4.74)	44.8 (15.7–80.0)	−11.5 (−30.1 to 10.6)
Marshall Islands	1.14 (0.753–1.64)	3.51 (2.33–5.00)	1.65 (1.15–2.33)	3.27 (2.30–4.52)	44.9 (18.5–81.7)	−6.97 (−24.5 to 15.4)
Micronesia (Federated States of)	2.70 (1.87–3.81)	3.72 (2.55–5.14)	3.24 (2.22–4.61)	3.41 (2.35–4.82)	20.1 (−5.02 to 48.5)	−8.41 (−26.5 to 12.3)
Nauru	0.327 (0.208–0.465)	4.25 (2.73–5.99)	0.314 (0.215–0.439)	3.74 (2.60–5.19)	−3.96 (−26.3 to 24.6)	−11.9 (−30.7 to 13.5)
Niue	0.0667 (0.0447–0.0971)	3.29 (2.19–4.82)	0.0638 (0.0428–0.0920)	3.18 (2.14–4.53)	−4.29 (−23.0 to 17.1)	−3.32 (−21.3 to 17.2)
Northern Mariana Islands	1.91 (1.21–2.91)	3.14 (2.07–4.56)	1.70 (1.04–2.60)	2.82 (1.77–4.21)	−10.9 (−34.0 to 18.8)	−10.1 (−29.8 to 13.6)
Palau	0.695 (0.454–1.02)	3.56 (2.36–5.09)	0.842 (0.547–1.22)	3.37 (2.25–4.96)	21.1 (−6.23 to 60.5)	−5.28 (−23.4 to 18.5)
Papua New Guinea	108 (70.1–156)	2.91 (1.90–4.23)	222 (146–332)	2.73 (1.79–3.97)	107 (68.1–146)	−6.15 (−24.0 to 12.0)
Samoa	4.20 (2.80–6.08)	3.32 (2.21–4.83)	5.42 (3.62–7.87)	3.14 (2.13–4.56)	28.9 (5.40–54.5)	−5.30 (−23.7 to 14.8)
Solomon Islands	9.59 (6.41–14.1)	3.48 (2.36–5.10)	17.3 (11.5–24.8)	3.33 (2.25–4.78)	80.2 (47.9–123)	−4.36 (−22.2 to 19.8)
Tokelau	0.0410 (0.0280–0.0588)	3.13 (2.12–4.46)	0.0431 (0.0286–0.0603)	3.08 (2.05–4.34)	5.18 (−14.9 to 28.1)	−1.46 (−19.3 to 21.4)
Tonga	2.38 (1.61–3.40)	3.23 (2.16–4.62)	2.71 (1.83–3.89)	3.09 (2.09–4.38)	14.2 (−5.35 to 37.2)	−4.36 (−21.2 to 15.5)
Tuvalu	0.289 (0.191–0.421)	3.33 (2.20–4.80)	0.367 (0.252–0.532)	3.17 (2.19–4.59)	26.7 (1.91–55.6)	−4.84 (−23.4 to 17.4)
Vanuatu	3.95 (2.65–5.67)	3.09 (2.11–4.41)	7.14 (4.82–10.5)	2.91 (1.95–4.19)	80.9 (48.9–116)	−5.94 (−22.6 to 13.5)
Southeast Asia	57,700 (35,800–74200)	14.2 (8.63–18.1)	88,600 (54,200–112,000)	12.7 (7.66–15.8)	53.7 (36.3–77.3)	−10.8 (−21.4 to 2.08)
Cambodia	915 (367–1380)	12.5 (4.72–19.1)	1640 (706–2730)	11.8 (4.87–19.9)	79.7 (33.4–139)	−5.33 (−29.4 to 25.5)
Indonesia	19,500 (8790–28,200)	12.4 (5.39–18.1)	30,400 (12,400–43,300)	11.2 (4.38–16.0)	55.8 (28.8–92.2)	−9.90 (−26.3 to 11.0)
Lao People's Democratic Republic	399 (159–645)	12.6 (4.90–20.4)	535 (243–836)	9.56 (4.05–15.3)	34.0 (−0.777 to 82.3)	−24.1 (−44.5 to 2.26)
Malaysia	970 (691–1360)	5.48 (3.97–7.50)	1810 (1300–2630)	5.75 (4.15–8.43)	86.1 (52.9–163)	4.93 (−14.7 to 52.9)
Maldives	7.43 (4.86–11.0)	4.23 (2.77–6.20)	24.1 (15.7–36.1)	4.28 (2.81–6.37)	225 (159–305)	1.17 (−17.2 to 21.7)
Mauritius	49.3 (33.0–72.2)	4.16 (2.77–6.12)	73.5 (48.7–107)	4.26 (2.88–6.14)	49.0 (23.2–85.0)	2.42 (−13.4 to 21.3)
Myanmar	4470 (1830–6860)	13.1 (5.17–20.2)	5340 (2420–8120)	10.1 (4.45–15.5)	19.6 (−11.1 to 63.3)	−22.5 (−42.5 to 5.55)
Philippines	11,200 (8270–14,100)	22.0 (16.2–27.4)	22,600 (16,000–28,200)	24.1 (17.4–29.9)	101 (73.3–134)	9.61 (−6.15 to 28.5)
Seychelles	5.64 (2.90–8.05)	7.83 (3.98–11.2)	9.99 (5.29–14.2)	8.12 (4.19–11.6)	77.0 (45.2–120)	3.74 (−15.0 to 27.2)
Sri Lanka	806 (563–1130)	4.62 (3.27–6.40)	1200 (830–1700)	4.57 (3.16–6.48)	48.3 (19.6–82.2)	−1.12 (−20.2 to 21.1)
Thailand	15,500 (11,200–21,100)	27.0 (19.4–36.3)	17,300 (12,800–23,500)	17.0 (12.8–23.1)	11.3 (−17.6 to 54.1)	−37.0 (−52.6 to 13.5)
Timor-Leste	42.7 (21.6–68.2)	8.14 (3.80–13.8)	78.8 (38.0–129)	8.36 (3.82–14.0)	84.5 (46.0–134)	2.76 (−19.8 to 30.1)
Viet Nam	3650 (2090–5380)	6.05 (3.39–8.86)	7530 (4400–10,900)	6.87 (3.89–9.90)	107 (69.7–156)	13.5 (−7.24 to 40.9)
**Sub-Saharan Africa**	**28,000 (17,400**–**51,300)**	**7.43 (4.33**–**14.5)**	**42,800 (27,000**–**77,100)**	**6.33 (3.73**–**12.1)**	**52.6 (22.0**–**88.4)**	**−14.9 (−29.0 to 3.99)**
Central Sub-Saharan Africa	1690 (1110–2700)	4.07 (2.47–7.10)	3250 (2130–5050)	4.04 (2.49–6.81)	92.2 (56.5–143)	−0.863 (−20.7 to 27.1)
Angola	354 (235–577)	4.35 (2.61–7.44)	707 (473–1090)	3.97 (2.53–6.49)	99.6 (49.3–164)	−8.71 (−29.7 to 23.4)
Central African Republic	103 (66.5–186)	5.19 (3.15–10.0)	162 (107–268)	4.93 (3.01–8.80)	57.3 (19.3–111)	−5.15 (−29.8 to 29.9)
Congo	104 (68.9–189)	5.68 (3.58–11.0)	184 (121–325)	4.95 (3.13–9.24)	78.1 (37.3–143)	−12.9 (−34.9 to 21.4)
Democratic Republic of the Congo	1070 (692–1730)	3.78 (2.25–6.77)	2090 (1340–3300)	3.90 (2.31–6.71)	94.7 (55.4–149)	3.27 (−20.7 to 36.7)
Equatorial Guinea	13.5 (8.93–21.7)	3.84 (2.32–6.60)	36.5 (23.5–60.3)	4.42 (2.67–7.96)	170 (110–256)	15.3 (−13.1 to 59.0)
Gabon	40.7 (24.8–74.0)	5.18 (3.07–10.1)	65.6 (41.9–114)	5.05 (3.14–9.18)	60.9 (21.9–111)	−2.40 (−27.9 to 32.5)
Eastern Sub-Saharan Africa	12,400 (6690–26,500)	10.2 (4.85–23.5)	19,000 (10,100–36,500)	8.20 (4.01–17.0)	52.6 (24.5–97.3)	−19.2 (−35.1 to 3.72)
Burundi	270 (140–575)	7.98 (3.71–18.5)	464 (247–906)	6.65 (3.14–14.1)	72.0 (23.3–140)	−16.7 (−41.3 to 22.1)
Comoros	25.0 (12.9–50.4)	7.60 (3.58–16.7)	41.8 (21.8–86.6)	7.19 (3.61–15.2)	67.1 (17.7–144)	−5.40 (−34.4 to 40.7)
Djibouti	35.3 (17.3–73.2)	10.7 (4.66–23.4)	86.3 (42.0–183)	10.4 (4.55–23.2)	145 (74.9–249)	−3.30 (−34.2 to 40.9)
Eritrea	198 (108–417)	9.52 (5.00–21.3)	343 (192–679)	8.42 (4.40–18.0)	73.0 (29.2–152)	−11.6 (−35.6 to 31.2)
Ethiopia	4790 (2100–10,500)	14.9 (5.78–35.5)	5250 (2390–11,300)	9.14 (3.61–20.8)	9.68 (−20.6 to 57.9)	−38.8 (−57.3 to 11.8)
Kenya	1100 (688–2110)	7.05 (4.07–14.9)	2430 (1450–4730)	7.95 (4.52–16.4)	121 (72.5–184)	12.8 (−16.2 to 48.9)
Madagascar	543 (318–1070)	6.48 (3.43–14.0)	1020 (602–1960)	6.20 (3.37–12.7)	88.7 (43.2–145)	−4.36 (−29.8 to 30.1)
Malawi	500 (286–1030)	8.70 (4.62–19.0)	781 (469–1490)	7.42 (4.18–15.1)	56.2 (16.0–110)	−14.7 (−37.0 to 18.6)
Mozambique	830 (429–1810)	9.10 (4.23–21.4)	1620 (875–3210)	10.5 (5.29–22.5)	95.1 (42.5–179)	15.9 (−17.7 to 67.5)
Rwanda	348 (172–735)	8.39 (3.82–19.2)	486 (263–922)	5.65 (2.83–11.7)	39.7 (−3.52 to 121)	−32.7 (−54.3 to 10.0)
Somalia	602 (289–1440)	12.6 (5.34–31.3)	1090 (486–2490)	11.7 (4.38–28.3)	81.7 (27.4–174)	−7.63 (−37.3 to 36.0)
South Sudan	356 (169–821)	10.2 (4.11–25.8)	548 (260–1300)	10.6 (4.51–27.5)	54.0 (13.7–115)	4.12 (−27.3 to 55.6)
Uganda	804 (439–1680)	7.29 (3.60–16.6)	1370 (799–2460)	6.24 (3.36–12.1)	70.9 (25.7–142)	−14.4 (−38.7 to 27.1)
United Republic of Tanzania	1450 (781–2960)	7.96 (3.92–17.6)	2370 (1290–4410)	6.91 (3.46–13.9)	63.6 (21.0–129)	−13.2 (−39.7 to 27.5)
Zambia	558 (310–1190)	12.6 (6.26–28.2)	1020 (475–2140)	10.3 (4.40–23.7)	82.4 (2.45–302)	−18.4 (−54.9 to 85.9)
Southern Sub-Saharan Africa	1910 (1460–2490)	3.87 (2.94–5.00)	2600 (1980–3490)	3.54 (2.71–4.73)	36.1 (22.9–51.2)	−8.48 (−17.6 to 4.18)
Botswana	49.3 (30.5–74.5)	4.46 (2.61–7.00)	75.2 (49.9–116)	3.62 (2.40–5.52)	52.5 (8.41–115)	−18.8 (−44.2 to 17.6)
Eswatini	37.0 (23.0–53.1)	6.39 (3.75–9.58)	45.8 (29.4–64.9)	5.56 (3.47–7.91)	23.8 (−9.14 to 63.8)	−13.0 (−36.9 to 18.0)
Lesotho	52.5 (35.0–71.4)	4.12 (2.67–5.60)	79.4 (52.2–114)	5.60 (3.57–8.14)	51.4 (13.3–101)	36.1 (−0.692 to 86.0)
Namibia	51.3 (33.6–70.5)	4.36 (2.69–6.00)	70.4 (48.3–99.5)	3.71 (2.48–5.23)	37.1 (6.55–68.7)	−14.9 (−35.1 to 8.42)
South Africa	1440 (1100–1890)	3.81 (2.96–4.94)	1890 (1410–2550)	3.37 (2.52–4.53)	31.5 (18.9–49.8)	−11.5 (−20.5 to 3.35)
Zimbabwe	285 (204–385)	3.83 (2.74–5.11)	441 (320–593)	4.03 (2.86–5.48)	54.8 (24.9–89.8)	5.20 (−18.5 to 30.8)
Western Sub-Saharan Africa	12,000 (7330–23,300)	7.22 (4.24–13.0)	18,000 (11,200–33,000)	6.14 (3.61–11.5)	49.6 (6.79–89.6)	−14.9 (−33.2 to 6.57)
Benin	308 (167–681)	7.48 (3.90–14.8)	485 (281–914)	6.11 (3.26–12.1)	57.3 (3.53–135)	−18.4 (−41.4 to 14.6)
Burkina Faso	586 (321–1280)	7.44 (3.79–15.1)	865 (483–1710)	6.45 (3.23–13.4)	47.5 (−22.3 to 110)	−13.3 (−43.6 to 20.7)
Cabo Verde	10.3 (6.05–18.2)	3.37 (1.89–6.14)	20.8 (12.0–40.8)	4.06 (2.29–8.21)	102 (49.2–177)	20.6 (−15.3 to 71.5)
Cameroon	822 (434–1720)	9.20 (4.86–18.9)	1290 (708–2600)	7.02 (3.64–15.0)	57.4 (6.04–124)	−23.7 (−46.6 to 7.05)
Chad	370 (196–831)	7.20 (3.59–14.7)	630 (350–1340)	6.55 (3.32–13.0)	70.4 (17.2–138)	−8.92 (−36.2 to 23.7)
Côte d'Ivoire	702 (421–1300)	7.82 (4.46–15.1)	1020 (607–1830)	6.21 (3.33–12.3)	44.7 (−3.73 to 105)	−20.6 (−46.7 to 16.2)
Gambia	61.4 (34.4–126)	7.79 (4.03–15.4)	97.2 (53.3–189)	7.39 (3.67–15.7)	58.4 (−9.87 to 127)	−5.09 (−38.8 to 31.4)
Ghana	1080 (615–1970)	9.21 (5.27–16.3)	2010 (1180–3530)	9.49 (5.40–16.8)	86.4 (18.0–189)	3.04 (−33.5 to 63.3)
Guinea	367 (192–865)	6.21 (3.35–12.6)	461 (267–907)	5.69 (3.07–11.6)	25.6 (−18.9 to 78.9)	−8.31 (−34.7 to 27.1)
Guinea-Bissau	82.0 (45.7–184)	11.4 (6.55–23.8)	93.3 (54.1–191)	8.73 (4.68–18.4)	13.8 (−44.6 to 75.6)	−23.2 (−52.7 to 7.87)
Liberia	105 (61.1–199)	6.11 (3.33–11.4)	193 (109–371)	6.07 (3.03–12.8)	83.1 (10.9–154)	−0.607 (−32.1 to 37.7)
Mali	557 (298–1360)	7.43 (3.68–14.8)	829 (481–1590)	6.24 (3.16–12.7)	48.9 (−23.1 to 124)	−16.0 (−46.9 to 15.9)
Mauritania	117 (68.2–233)	7.24 (3.91–15.0)	166 (89.9–341)	6.22 (3.09–13.3)	41.7 (−9.57 to 96.3)	−14.1 (−39.5 to 15.3)
Niger	517 (265–1440)	6.82 (3.32–14.2)	687 (403–1320)	5.11 (2.52–10.2)	33.1 (−40.4 to 121)	−25.1 (−56.1 to 6.37)
Nigeria	5500 (3200–10,900)	6.74 (3.81–11.8)	7840 (4900–14,600)	5.41 (3.10–9.90)	42.6 (8.52–91.6)	−19.7 (−40.8 to 8.98)
Sao Tome and Principe	5.82 (3.37–10.9)	6.68 (3.79–13.0)	8.79 (5.12–16.6)	6.05 (3.32–12.1)	51.1 (7.59–104)	−9.41 (−33.2 to 19.4)
Senegal	422 (257–836)	6.66 (3.75–12.3)	619 (328–1300)	6.29 (3.05–13.5)	46.6 (−36.4 to 120)	−5.64 (−44.7 to 32.3)
Sierra Leone	195 (112–485)	6.22 (3.45–11.7)	300 (175–530)	5.54 (2.99–10.2)	54.0 (−20.9 to 129)	−10.9 (−43.8 to 25.1)
Togo	208 (129–399)	7.57 (4.29–15.1)	353 (209–651)	6.76 (3.66–13.2)	70.0 (1.22 to 154)	−10.7 (−38.6 to 25.9)

### Regions

The age-standardised incidence rate in 2021 was highest in eastern Europe at 3560 per 100,000 (95% UI 3000–4240), followed by central Asia at 1880 per 100,000 (1600–2230) and Andean Latin America at 1730 per 100,000 (1440–2100). By contrast, the age-standardised incidence was lowest in western sub-Saharan Africa at 606 per 100,000 (492–756). The percentage change in incidence rate between 2000 and 2021 differed between the GBD regions. Twelve GBD regions, central Europe, eastern Europe, Australasia, high-income North America, western Europe, high-income Asia Pacific, east Asia, southeast Asia, Oceania, eastern sub-Saharan Africa, southern sub-Saharan Africa, and central sub-Saharan Africa, showed decreased age-standardised incidence rates of urolithiasis, whereas the remaining GBD regions had increased age-standardised incidence rates between 2000 and 2021. Central Latin America and tropical Latin America saw the greatest increases in the age-standardised incidence rate during the study period at 32.1% (28.1–35.6) and 16.9% (13.4–20.5), respectively. Central Europe and east Asia, on the other hand, had the biggest decreases in the age-standardised incidence rate at 26.8% (24.7–28.8) and 35.1% (30.7–38.4), respectively.

In 2021, the age-standardised death rate of urolithiasis was below 0.5 per 100,000 in all GBD regions. The highest age-standardised death rates of urolithiasis were observed in eastern Europe, central Asia, and southeast Asia, while the lowest age-standardised death rates were observed in southern sub-Saharan Africa, southern Latin America, and north Africa and the Middle East. Twenty GBD regions saw an increase in death counts due to urolithiasis from 2000 to 2021, ranging from 53.6% (eastern Europe) to 371% (high-income Asia Pacific). Nonetheless, there was a 25.5% decrease in death counts in the central Europe region between 2000 and 2021.

Variation in the age-standardised death rate of urolithiasis trend was seen in GBD regions. Between 2000 and 2021, the largest increases in the age-standardised death rate of urolithiasis were observed in high-income Asia Pacific (92.1% [95% UI 70.1–111]), high-income North America (76.2% [65.1–88.1]), and Topical Latin America (75.5% [63.9–88.2]). However, decreased age-standardised death rates of urolithiasis were observed in south Asia, east Asia, Oceania, central sub-Saharan Africa, eastern sub-Saharan Africa, southern sub-Saharan Africa, western sub-Saharan Africa, central Europe, southern Latin America, western Europe, and central Latin America.

In 2021, the highest DALYs counts were observed in south Asia (166,000 [95% UI 114,000–231,000]), east Asia (117,000 [90,200–149,000]), eastern Europe (72,400 [62,100–87,900]), and southeast Asia (88,600 [54,200–112,000]), while the lowest were in Australasia (2770 [2230–3450]) and Oceania (315 [209–461]). The regional change in DALY counts from 2000 to 2021 ranged from −28.8% to 159%.

From 2000 to 2021, the percentage change in DALY count was highest in Tropical Latin America (159% [142–178]), central Latin America (87.6% [72.0–106]), and north Africa and the Middle East (83.2% [61.1–108]), while two regions showed decreases: east Asia (12.1% decline [14–27]) and central Europe (28.8% decline [20.1–34.9]). In 2021, the highest age-standardised DALY rates were in eastern Europe (22.8 per 100,000 [19.4–28.1]), southeast Asia (10.1 per 100,000 [6.8–14.1]), central Asia (12.9 per 100,000 [10.6–16.1]) and Tropical Latin America (10.4 per 100,000 [9.34–11.7]), while the lowest were in north Africa and the Middle East (4.55 per 100,000 [3.2–5.9]), southern sub-Saharan Africa (3.5 per 100,000 [2.7–4.7]) and central sub-Saharan Africa (4.0 per 100,000 [2.5–6.7]).

The regional change in the age-standardised rate of DALYs from 2000 to 2021 ranged from −55% to 33%. From 2000 to 2021, the percentage change in age-standardised rates of DALYs was highest in Tropical Latin America with 54.5% (95% UI 45–64.5), the Caribbean (18.4% [6.5–31.4]), and high-income North America (12.1% [3.3–23.1]). The largest decreases were noted in southeast Asia (10.8% [1.9–21.1]), central Europe (35.7% [29.0–41.1]), and east Asia (48.9% [32.5–58.1]).

**Fig. 3:**
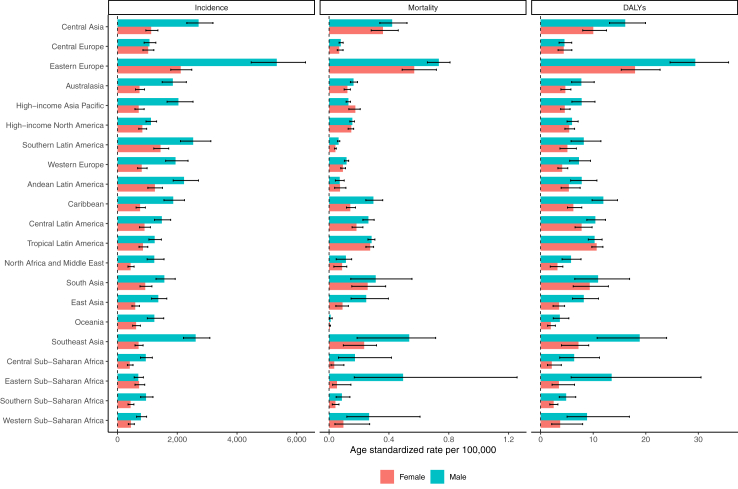
Incidence, deaths and DALYs rates of Urolithiasis in 21 GBD regions, 2021 Bars represent mean incidence, death and DALYs rate, and error bars represent 95% uncertainty intervals.

**Fig. 4:**
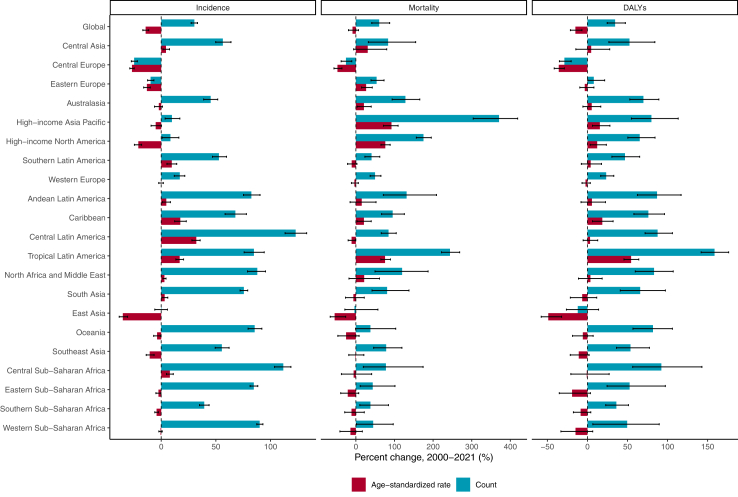
Percentage change in incidence, deaths and DALYs counts and rates of Urolithiasis in 21 GBD regions, 2000–2021 Bars represent mean incidence, death and DALYs rate and counts, and error bars represent 95% uncertainty intervals.

In 2021, the lowest female to male ratio of incidence rates was observed in southeast Asia at 1:3.5, north Africa and the Middle East at 1:3.1, and high-income Asia Pacific at 1:2.6. Conversely, nearly equivalent female to male ratios were observed in Tropical Latin America at 1:1.4, central Europe at 1:1.06, and eastern sub-Saharan Africa at 1:1.01. Slight increases in the incidence of urolithiasis cases in male were noted in the Caribbean, north Africa and the Middle East, southern sub-Saharan Africa, Tropical Latin America, and central sub-Saharan Africa, whereas the incidence of female urolithiasis has been rising in other GBD regions (Table 1, Table 2, and Supplementary File S6, Figs. 3 and 4).

### Burden by socio-demographic index

Middle-income countries made up the majority of urolithiasis incident cases, deaths, and DALYs in 2021. The age-standardised incidence rate was 837 per 100,000 (95% UI 688–1034) in low SDI, 1132 (940–1386) in low-middle SDI, 1281 (1070–1552) in middle SDI, 1170 (976–1416) in high SDI, and 1443 (1210–1734) in high-middle SDI. Age-standardised death rates in 2021 were less than 1 per 100,000 in all SDI quintiles.

In 2021, the highest numbers of deaths were recorded in the mid-SDI quintiles: middle SDI (5400 [3600–6600]), high-middle SDI (4000 [3400–4600]), low-middle SDI (3900 [2500–5400])) high SDI (3400 [2800–3800]), and low SDI (1000 [600–2000]). In 2021, the age-standardised mortality rate from urolithiasis was 0.14 per 100,000 (0.12–0.16) in high SDI, 0.2 per 100,000 (0.18–0.23) in high-middle SDI, 0.21 per 100,000 (0.12–0.42) in low SDI, and 0.29 per 100,000 (0.18–0.40) in low-middle SDI regions.

In 2021, most of the global DALYs were recorded in the mid-SDI quintiles: middle SDI (230,000 [95% UI 183,000–283,000]), high-middle SDI (150,000 [126,000–183,000]), low-middle SDI (161,000 [118,000–214,000]), high SDI (100,000 [84,000–121,000]), and low SDI (51,000 [36,000–79,000]). The age-standardised DALYs rate of urolithiasis across SDI quintiles was 5.8 per 100,000 (4.7–7.1) in high SDI, 8.1 per 100,000 (6.8–10.1) in high-middle SDI, 7.5 per 100,000 (5–12.3) in low SDI, 8.4 per 100,000 (6.7–10.4) in middle SDI, and 10 per 100,000 (7.2–13.3) in low-middle SDI in 2021.

Death counts across SDI quintiles increased between 2000 and 2021; the largest increase was in the high SDI quintile (134.5% [95% UI 114.2–151.0]), and the smallest was in the high-middle SDI quintile (27.6% [13.7–50.1]). Variations were observed in the age-standardised death rate of urolithiasis: high SDI, 34.0% increase (24.3–43%); low-middle SDI, 20% increase^17^, ^18^, ^19^, ^20^, ^21^, ^22^, ^23^, ^24^, ^25^, ^26^; low SDI 12.4% decrease (−30 to 12.4); high-middle SDI, 24.3% decrease (10.4–32.8), and middle SDI, 26.2% decrease (−5.0 to 37.6).

**Fig. 5:**
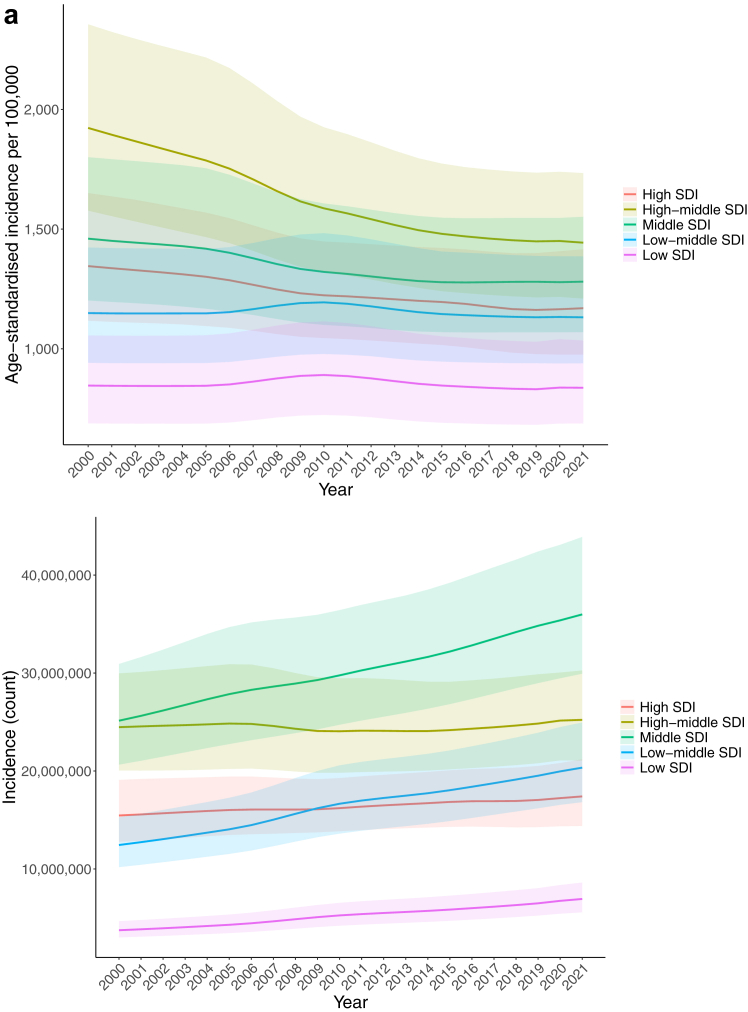

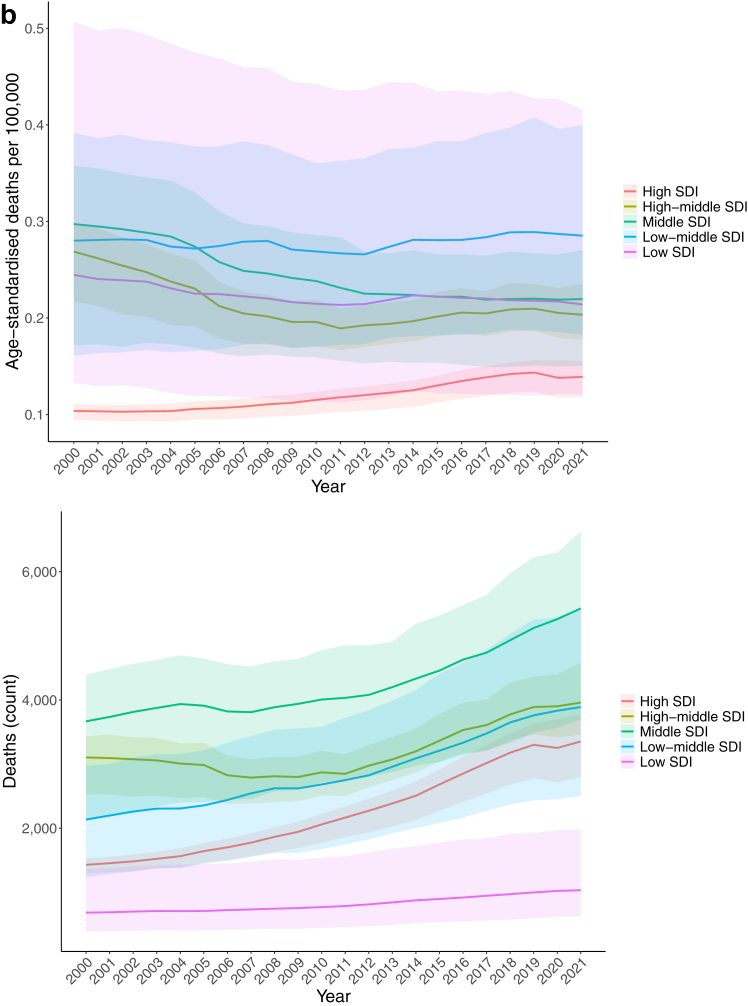

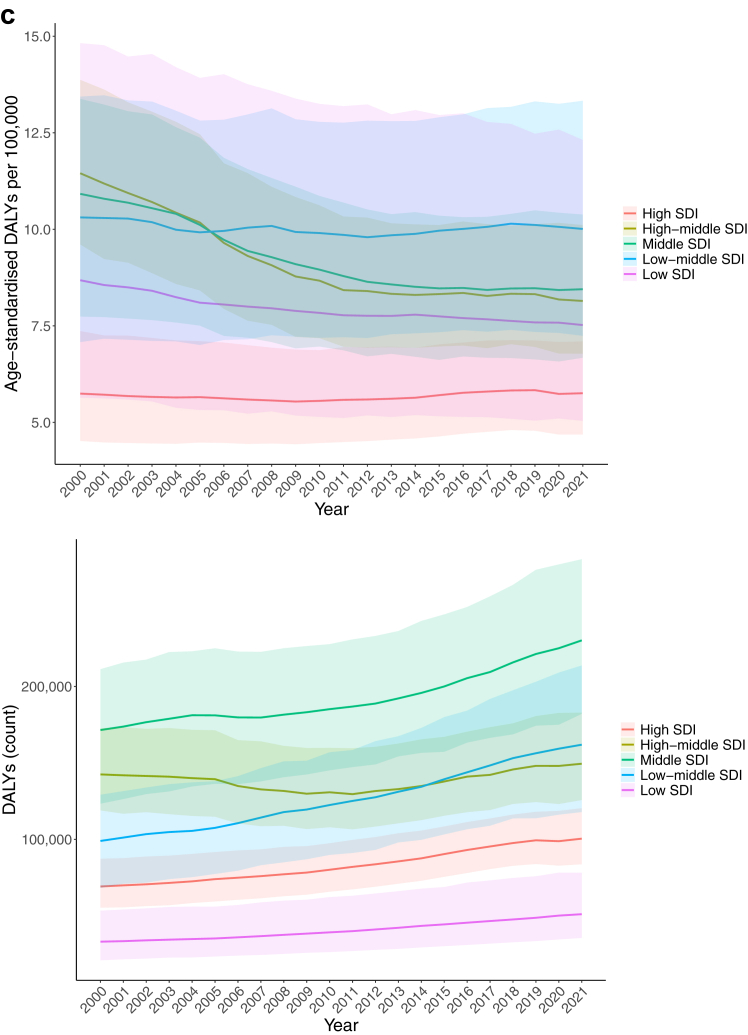
a: Temporal trend of age-standardised rate and counts of incidence by SDI quintile, 2000–2021. The top panel illustrates changes in incidence rates, and the bottom panel illustrates changes in incidence counts. Countries were assigned to SDI quintiles on the basis of their SDI in the year 2021. The shaded areas represent 95% uncertainty intervals. b: Temporal trend of age-standardised rate and counts of deaths by SDI quintile, 2000–2021. The top panel illustrates changes in age-standardised rates, and the bottom panel illustrates changes in absolute death counts. Countries were assigned to SDI quintiles on the basis of their SDI in the year 2021. The shaded areas represent 95% uncertainty intervals. c: Temporal trend of age-standardised rate and counts of DALYs by SDI quintile, 2000–2021. The top panel illustrates changes in DALYs rates, and the bottom panel illustrates changes in absolute counts of DALYs. Countries were assigned to SDI quintiles on the basis of their SDI in the year 2021. The shaded areas represent 95% uncertainty intervals. DALY, disability-adjusted life-year; SDI, Socio-demographic Index.

From 2000 to 2021, the percentage change in DALYs across SDI quintiles was as follows: low-middle SDI 63.7% (95% UI 42.6–90.9), low SDI 54.6% (29.7–86.8), high SDI 45.2% (35.4–57.4), middle SDI 34.1% (21.5–57.4), and high-middle SDI 4.9% (−2.8 to 14.9). From 2000 to 2021, the age-standardised DALY rate decreased in all SDI quintiles: high-middle SDI (28.9% decline [23.0–34.4]), middle SDI (22.6% [10.9–30.5]), low SDI (13.3% [1.8–26.1]), low-middle SDI (2.9% [–12.9 to 15.8]), and high SDI (0.2% [–6.0 to 4.2]) (Fig. 5).

### National level

Between 2000 and 2021, there was a notable increase in urolithiasis incidence cases reported across 179 countries, with 112 of them observing a rise in age-standardised incidence rates. Globally, incident cases of urolithiasis in 2021 were highest in China (19.1 million [95% UI 15.7–23.7]), India (18.6 million [15.1–22.9], Russia (7.0 million [5.94–8.38]), and the USA (4.23 million [3.59–5]). In 2021, the age-standardised rate of incident cases of urolithiasis ranged from 573 to 3770 per 100,000 population worldwide; the highest rates were observed in Russia (3530 [2980–4190]), Ukraine (3770 [3160–4490]), and Belarus (3520 [2950–4240]) per 100,000, whereas the lowest rates were seen in Niger (573 [463–717]) and Togo (577 [469–728]) per 100,000.

Globally, the percentage change of the incidence cases of urolithiasis from 2000 to 2021 was highest in Qatar (508% [95% UI 467–549]), Jordan (332% [290–372]), and United Arab Emirates (357% [294–427]), whereas the greatest percentage decreases were noted in Georgia (28.2% decline [15.1–38.6]), Bulgaria (31.1% [26.4–49.1]), and Poland (42.1% [33.5–49.2]).

Urolithiasis-related death counts increased in 179 countries, with 89 of them witnessing a rise in age-standardised death rates between 2000 and 2021. In 2021, the highest numbers of deaths due to urolithiasis were observed in China, India, and Russia, which reflected the population size of these countries. However, the highest age-standardised death rates were observed in Armenia (1.8 per 100,000 [95% UI 0.9–4]), Kazakhstan (1.3 [0.9–1.7]), Trinidad and Tobago (0.9 [0.7–1.2]), Russia (0.8 [0.7–0.9]), the Philippines (0.7 [0.4–0.9]), Belarus (0.7 [0.6–0.9]), and Latvia (0.7 [0.5–0.9]) per 100,000 in 2021. From 2000 to 2021, the temporal trend of death counts ranged from −66% to 956%. The highest percentage change of death counts was noted in Saudi Arabia (956% [105–2960]), Taiwan (province of China) (353% [265–455]), Kuwait (23,300 [16,400–32,000]), and Libya (524% [95.4–1044]).

Increased urolithiasis burden contributed to DALY counts observed in 181 countries, with 74 of them having an increase in age-standardised DALYs rates between 2000 and 2021. In 2021, DALY counts were highest in India, China, Russia, Indonesia, Brazil, and the USA. Age-standardised DALY rates per 100,000 were highest in Kazakhstan (33.3 [95% UI 26.1–43.1]), Russia (25.7 [22.1–31.2]), the Philippines (24.2 [17.3–29.7]), Belarus (25.4 [20.3–30.9]), Trinidad and Tobago (30.1 [23.7–38.1]), and Latvia (24.1 [19.5–30.7]), while the lowest were in Guam (2.8 [1.8–4.2]), Papua New Guinea (2.7 [1.8–4.0]), Saudi Arabia (2.7 [1.8–4.1]).

From 2000 to 2021 the percentage change in DALY counts was highest in Qatar (385% [95% UI 239–632]) and United Arab Emirates (332% [188–520]), and the largest decreases were observed in Slovakia (33.3% decline [33–45.411.0]), Bulgaria (41.4% [−54.2 to 25.2]), and Poland (37.8% [49–45.1-26.1]).

The highest female to male ratios were observed in Mozambique at 1.1:1, Poland at 1.06:1, Comoros at 1.06:1, and Somalia at 1.04:1. Conversely, the highest male to female incidence ratios of urolithiasis were observed in Bahrain at 5.7:1, Qatar at 8.6:1, and the United Arab Emirates at 10:1 in 2021. Furthermore, we observed an overall increase in the incidence of urolithiasis across all countries, accompanied by a narrowing of the gap in the female to male ratio between 2000 and 2021 (Tables 1 and 2, and Supplementary File Table S6, Fig. 1).

## Discussion

We report comprehensive, standardised, and updated evidence on the global burden and trends of urolithiasis from 2000 to 2021. Between 2000 and 2021, global age-standardised incidence and DALY rates decreased significantly, and the age-standardised death rate was fairly stable, with a point-estimate suggesting a small decrease but an uncertainty interval that does not exclude the possibility of no change; however, these observations contrast with the worldwide increase seen in incident cases, deaths, and DALY counts related to urolithiasis over the past two decades.

The findings from this study, showing a notable increase in urolithiasis incident cases alongside a decreased global age-standardised rate, contrast with certain population-based studies indicating a stable incidence post-2000.^43^ This discrepancy underscores the importance of data standardisation to a reference standard and the consideration of demographic changes in interpreting and comparing study results. Finding from this study noted that there was simultaneous increase in incident cases and decrease in age-standardised incidence rates of urolithiasis globally, alongside varying trends at regional and national levels. These findings are consistent with multiple epidemiological studies.^4^^,^^8^^,^^25^^,^^27^^,^^44^, ^45^, ^46^ The rise in incident cases of urolithiasis despite a decrease in age-standardised rates suggests that population growth and demographic shifts have occurred alongside advances in preventive measures or reduction in risk factor exposure. Over the past two decades, there has been a decreasing trend in global age-standardised incidence rates with diverse regional and national variations. We have noted that there was no a new innovative preventive intervention for urolithiasis worldwide for the last two decades; however, numerous urological associations have been providing evidence-based recommendations on preventive strategies for urolithiasis. There is no universally accepted preventive method, necessitating a range of interventions to address the diverse causes and complications associated with urolithiasis.^47^, ^48^, ^49^ These interventions include metabolic evaluation and recurrence prevention, infection control and management, genetic counselling and screening, education and awareness initiatives, as well as consideration of dietary, lifestyle, and environmental factors.^47^^,^^50^, ^51^, ^52^, ^53^ For instance, metabolic evaluation and recurrence prevention involve identifying metabolic imbalances and risk factors predisposing individuals to stone formation. Strategies such as increasing water intake, reducing salt consumption, adjusting urine acidity, or prescribing specific medications are recommended to prevent common types of stones like calcium oxalate or uric acid stones.^52^^,^^53^ Furthermore, the recommendations and recent evidence emphasise the importance of detecting and treating urinary tract infections, particularly in cases of struvite or infection stones, to mitigate the risk of urolithiasis worsening. Although there is no universally accepted screening modality for urolithiasis, genetic counseling and screening using molecular techniques, biochemical assays, or family history analysis can aid in identifying and testing for genetic disorders associated with urolithiasis, particularly in cases of cystine or rare stones. Education and awareness initiatives to disseminating information and guidance to both patients and the general public through various channels, such as leaflets, posters, websites, social media, or mass media campaigns, can contribute to improving the prevention of urolithiasis.^50^ These modifications in social conditions, disease management protocols, access to advanced interventions, and education and awareness campaigns may contribute to the decline in the global age-standardised incidence rate of urolithiasis.

Over the past two decades, we also demonstrate a declining global age-standardised DALY rate driven largely by declining age-standardised incidence rates. The reduction in global age-standardised DALY rates suggests a decrease in the overall burden of disability attributed to urolithiasis worldwide for the past two decades. The decline in age-standardised DALY rates may be linked to successful preventive measures, lifestyle modifications, and public health initiatives.

These improvements in age-standardised incidence and age-standardised DALY rates at the global level were not seen consistently across regions. Twelve GBD regions showed declining trends in the age-standardised incidence rate of urolithiasis between 2000 and 2021, and the remaining nine GBD regions had an increasing trend of age-standardised rates of urolithiasis. A significant increase in the age-standardised incidence rate of urolithiasis was observed in Central America, Tropical Latin America, and the Caribbean regions, whereas notable declines were observed in east Asia, eastern Europe, high-income North America, and central Europe.

We could not find newly emerged risk factors that could account for the rise of urolithiasis in some Latin American or specific preventive measures implemented specifically in eastern Europe, central Europe, and east Asia. The former could be true changes related to changes in ambient temperature, diet or other risk factors, or could be increased detection not fully corrected for by our analysis.

The latter would be consistent with changes in eating habits, physical activity, community health-seeking behaviours, accessibility and capacity of health care systems, changes in metabolic syndrome, and advancements in the diagnosis and treatment of urinary stones. The general recommendation by EAU, AUA, and CUA to prevent urolithiasis is to drink more water to stay hydrated and lower urine concentration while aiming for a daily urine output of more than 2.5 L with a fluid intake above 3 L per day.^47^, ^48^, ^49^ Urolithiasis management has historically depended on factors such as stone location, size, burden, anatomy, comorbidity, access to specialists, and development of endourological interventions.^47^, ^48^, ^49^

We also found heterogeneous trends in incidence and DALYs due to urolithiasis at the national level from 2000 to 2021. In 2021, more than half of the incident cases of urolithiasis were recorded in India, China, Russia, and the USA. Between 2000 and 2021, there was a notable increase in urolithiasis incident cases reported across 179 countries, with 112 of them observing a rise in age-standardised incidence rates. Urolithiasis DALY counts increased in 181 countries, with 74 of them having an increase in age-standardised DALY rates between 2000 and 2021. From 2000 to 2021, incidence and DALYs due to urolithiasis increased in most nations, and especially in Qatar, Jordan, United Arab Emirates, and Bahrain. Geographical and temporal variations of the incidence rates of urolithiasis come from diagnostic capability, health care systems, economic change, urbanisation and industrialisation, and changes in distribution of risk factors.^4^^,^^8^^,^^9^^,^^13^^,^^17^^,^^20^^,^^54^ Lifestyle change, diet, and change in the environment highly influenced the geographical variation in incidence of urolithiasis.^14^ Changes of lifestyle have mainly contributed to change of the burden of urolithiasis in developed countries, while diet has contributed in developing countries. Diets such as those high in animal protein; low in alkali, magnesium, and citrate; and high in oxalate- and calcium-containing foods cause negative calcium balance, low urine pH, and low urinary excretion of citrate, potassium, and magnesium,^1^^,^^14^^,^^55^ which all favour stone formation.

Even though environmental changes impact both developed and developing nations almost equally,^14^^,^^56^ global warming can lead to dehydration, causing high urine concentration and low urine volume, which in turn increase the likelihood of stone formation.^57^ This trend could potentially elevate the global age-standardised incidence rate of urolithiasis. Meteorological Organization (WMO) reported Central America, Tropical Latin America, and the Caribbean regions as experiencing the highest temperatures globally, our findings provide compelling evidence that these areas have indeed witnessed a significant increase in the age-standardised incidence rate of urolithiasis over the past two decades.^58^

However, contrary to this expectation, we have observed a decrease in the age-standardised incidence rate, indicating that the change in the incidence rate of urolithiasis is influenced by a complex interaction of multiple factors. Prior authors have noted high incidence in oil-rich Gulf states and suggested this is due to the combination of the region's environment and climate, socioeconomic status of the population, and lifestyle and dietary habits of the community—which includes a high level of affluent population—sedentary lifestyle and high consumption of animal products, and the hot and dry climate of the region.^56^^,^^59^ These observations highlight the inconsistent effectiveness of preventive interventions for urolithiasis across nations. There is a clear need for collaborative efforts at the national, regional, and global levels to address this disparity in burden and ensure equitable access to urolithiasis management worldwide. Such efforts could involve sharing best practices, coordinating research initiatives, improving health care infrastructure, and promoting education and awareness campaigns to enhance urolithiasis prevention on a global scale. In contrast to the significant decreases observed in the global age-standardised incidence and DALY rates for urolithiasis, the age-standardised mortality rate globally was relatively stable. This disparity could reflect lesser progress made towards reducing mortality compared to the advancements in curbing incidence. This interpretation would underscore the necessity to reevaluate current strategies focusing on preventive and therapeutic measures, which seem to be more effective at addressing morbidity than mortality. These findings emphasise the requirement for innovative interventions that target reducing urolithiasis-related mortality. As with temporal trends in age-standardised incidence rates, age-standardised mortality rate trends displayed some heterogeneity across regions, with two regions, central Europe and east Asia, experiencing significant improvements in age-standardised mortality between 2000 and 2021. The analysis of age-standardised mortality rates from urolithiasis reveals a striking paradox: while the global trend remains relatively stable, certain high-income regions—specifically high-income Asia–Pacific and high-income North America—have witnessed significant increases in mortality associated with kidney stones over the past two decades. This trend suggests that, despite advancements in healthcare and improved living standards in these affluent areas, individuals are facing increasingly severe outcomes related to kidney stones. One potential explanation for this troubling increase is the rising prevalence of co-morbidities among patients who develop kidney stones in these regions. Many individuals suffering from urolithiasis also contends with pre-existing health issues, such as obesity, diabetes, hypertension, or metabolic disorders.^60^^,^^61^ These co-morbid conditions can heighten the risk of complications when kidney stones occur, leading to more severe cases and complicating treatment options. As a result, patients with these underlying health problems may experience worse outcomes, contributing to the rising mortality rates associated with urolithiasis. While advancements in medical technology and healthcare access should ideally reduce mortality rates, the increasing burden of chronic diseases may counteract these benefits, highlighting the need for a more integrated approach to managing health that addresses both urolithiasis and its related co-morbidities Similarly, urolithiasis-related death counts increased in 179 countries, with 89 of them witnessing a rise in age-standardised death rates during the same period. This suggests differential trends in access to effective diagnosis and treatment to avert fatal outcomes. This may depend on health care provider awareness, diagnostic technology, efforts in early detection, and innovations in treatments targeting severe urolithiasis cases and their related complications. These include advanced endourology, shockwave lithotripsy (SWL), and robotic surgery. The ureteroscopy and SWL procedures account for over 90% of urolithiasis management.^54^ Recent high-level meta-analyses of randomised controlled trials have revealed that the innovative mini percutaneous nephrolithotomy (PCNL) and ultra-mini/super-mini PCNL techniques have superseded the traditional PCNL method.^55^, ^56^, ^57^, ^58^ These new approaches have achieved a stone-free rate of over 95% with minimal morbidity.^55^^,^^56^ These advancements in effective intervention within the health care system facilitate the management of urolithiasis,^47^^,^^49^^,^^59^ but are not universally available.

Urolithiasis incidence, deaths, and DALYs increased across all SDI quintiles from 2000 to 2021, with a notable concentration of global cases, deaths, and DALYs in the mid-SDI quintiles. The observed rise in urolithiasis cases in mid-SDI quintiles may be attributed to factors like population growth, demographic shifts, advances in diagnostic technologies, increased health care provider awareness, and better health care access. An analysis of the age-standardised incidence rate (ASIR) concerning Socio-demographic Index (SDI) quintiles revealed that high-middle SDI countries exhibited the highest ASIR, whereas low SDI countries displayed the lowest ASIR. These observations are in line with the epidemiological transition model, emphasising the relationship between socioeconomic development and disease incidence patterns.^62^ Trends in age-standardised urolithiasis incidence rates varied over the past two decades, with decreases in incidence rates seen in middle, high-middle, and high SDI countries, while increases were noted in low and low-middle SDI countries. These divergent trends could reflect improving detection in less developed countries concurrent with successful preventive efforts targeting urolithiasis risk factors and interventions in more developed countries highlighting potential benefits of public health education, lifestyle modifications, and advanced intervention strategies, particularly in mid-SDI quintiles compared to low SDI regions. In 2021, the age-standardised death rates were below 1 per 100,000 in all Socio-demographic Index (SDI) quintiles. A statistically significant decrease in the age-standardised DALYs rate of urolithiasis was observed in the mid-SDI quintile. However, while low SDI showed a decrease in age-standardised DALYs in point estimation without statistical significance, high SDI remained stable without statistical significance from 2000 to 2021. These observations may be a result of the active efforts in newly emerged mid SDI countries to enhance medical and surgical care, as well as the implementation of effective preventive measures. This is contrasted with high-income countries that have maintained optimal standards without substantial introduction of innovative treatments over the past two decades. The positive impact of interventions, preventive measures, and health care strategies on reducing disability related to urolithiasis is evident in these findings. Successful public health initiatives, advancements in treatment, and early interventions have the potential to improve outcomes. The enhancements in health care delivery, increased access to treatment, and enhanced disease management likely contribute to the decline in age-standardised DALY rates. Continuous monitoring of DALY counts and age-standardised rates allows for a comprehensive assessment of intervention effectiveness, shifts in disease burden, and advancements in addressing disability and mortality associated with urolithiasis.^44^

Our investigation unveiled that urolithiasis predominantly affected adult males, showcasing significant variability across regions and nations, with a female to male distribution ranging from 1:10 in the United Arab Emirates to 1.1 to 1 in Mozambique. These gender-specific variations resonate with findings from diverse epidemiological studies conducted worldwide, shedding light on the intricate interplay between gender, regional factors, and disease prevalence within the realm of urolithiasis.^4^^,^^46^^,^^63^^,^^64^ According to data from the National Health and Nutrition Examination Survey (NHANES), the period 2015–2016 recorded a 13.0% prevalence of urolithiasis in men and 9.8% prevalence in women.^46^ Intriguingly, specific countries like Mozambique, Poland, Comoros, Somalia, Zambia, Rwanda, Tanzania, Serbia, Kenya, Eritrea, and Madagascar exhibited a slight female predominance in incident cases in 2021.

Interestingly, we found that the incidence of urolithiasis in females increased worldwide from 2000 to 2021. The male and female ratios have narrowed, which is consistent with previous research investigations.^65^ Previous epidemiological studies showed that males were two to three times more affected by urolithiasis than females, as measured by hospital admissions, emergency visits, and outpatient visits.^12^ Our findings in this study reinforced previous population-based findings which reported that the overall incidence rate of urolithiasis increased by 1.9% per year for females and declined by 1.7% for males.^43^ Overall utilisation of urolithiasis-related procedures increased by 52% for women and 22% for men between 1997 and 2002.^11^ The rational evidence remains to be investigated; however, increased obesity,^4^^,^^11^ change of diet and lifestyle,^4^^,^^43^ and utilisation of imaging technology could be reasons for this observation.^43^

The occurrence of urolithiasis was not uniformly distributed across age groups. We found that urolithiasis was uncommon below 20 years and above 70 years. The incidence rate of urolithiasis peaked at 55–59 years in both males and females in our study these findings were consistent with previous epidemiological evidence.^43^^,^^66^

There are several limitations in the study, primarily related to the quality and quantity of available data. The main sources of data used in the non-fatal estimation were administrative records from medical facilities and insurance claims. These data sources have inherent limitations pertaining to bias and representativeness. Health care-seeking behaviours and access to quality health care, for instance, vary widely depending on numerous factors like trust in the health care system, education, household income, health insurance status, and location-specific characteristics (eg, urban/rural). Therefore, each facility has varying levels of care access, which makes it difficult to assess how well each data source represents and covers population for each data source for every demographic group we estimate in GBD. In addition, when we converted hospital discharge data to population-level estimates of urolithiasis, accounting for inpatient and outpatient encounters as well as primary and secondary diagnoses, we used the adjustment factors derived from insurance claims data from the USA. Although the adjustment factors were modelled as a function of the HAQ Index, we acknowledge that the adjustment factors may underestimate or overestimate the total cases of urolithiasis in different locations, particularly with differences in diagnostic approaches and preferred imaging modalities in various regions.

Furthermore, the level of healthcare access in a particular location may improve over time, creating apparent increases in incidence; the use of HAQ Index to model utilisation and inpatient correction factors may only partially overcome this trend toward improved detection. Another limitation in our estimation process was data scarcity issues. Despite the international administrative data we used in our analyses, we lacked data in many regions, including sub-Saharan Africa, Australasia, south Asia, Andean Latin America, and eastern Europe. We used multivariate geospatial analyses to estimate the non-fatal and fatal estimates of urolithiasis in these regions by borrowing information from the nearby data-rich locations and years. The estimates in locations with limited or no data had wider confidence intervals to reflect the greater uncertainty. However, additional data on the epidemiology of urolithiasis would be of much use in future iterations.

Our study suggests that globally, after accounting for population growth and ageing, incidence is decreasing overall, but there are heterogeneous trends that policy makers should take into account. First, as more of the population survives into high-burden age groups, developing countries will have to contend with increased total burden of urolithiasis. Second, global age-standardised incidence is decreasing faster and more definitively than age-standardised death rates, which suggests improvement in risk factors but lagging urological health care access and quality. Third, these global trends are not uniform; some regions and SDI quintiles—especially in central Latin America and Tropical Latin America as well as low and low-middle SDI countries—are demonstrating rising age-standardised incidence rates. Lifestyle changes, dietary habits, genetic predispositions, environmental factors, and improved diagnostic technologies may influence the rise in urolithiasis cases and will necessitate improved access to urological care to prevent morbidity and premature mortality. Likewise, trends differ between males and females, suggesting that risk factor mitigation that has been effective in males should be reexamined and adapted for application to female populations.

## Contributors

GBD 2021 Urolithiasis Collaborators please see Supplementary Appendix 2 for detailed list of affiliations and contributions.

## Data sharing statement

GBD study 2021 data sources were available online from Global Health Data exchange tool (https://collab2021.healthdata.org/gbd-results/).

## Editor note

The Lancet Group takes a neutral position with respect to territorial claims in published maps and institutional affiliations.

## Declaration of interests

S Bhaskar reports grants or contracts from the Japan Society for the Promotion of Science (JSPS), Japanese Ministry of Education, Culture, Sports, Science, and Technology (MEXT) and JSPS and the Australian Academy of Science; leadership or fiduciary roles in board, society, committee or advocacy groups, paid or unpaid, with Rotary District 9675, Sydney, Australia; Global Health & Migration Hub Community, Global Health Hub Germany, Berlin, Germany; PLOS One, BMC Neurology, Frontiers in Neurology, Frontiers in Stroke, Frontiers in Public Health, Journal of Aging Research, and BMC Medical Research Methodology; College of Reviewers, Canadian Institutes of Health Research, Government of Canada; World Headache Society, Bengaluru India; Cariplo Foundation, Milan, Italy; National Cerebral and Cardiovascular Center, Department of Neurology, Suita, Osaka, Japan; Cardiff University Biobank, Cardiff, UK, all outside the submitted work. B Bikbov reports grants or contracts from the European Commission, University of Rome, and Politecnico di Milano; supporting for attending meetings and/or travel from the European Renal Association; leadership or fiduciary roles in board, society, committee or advocacy groups, unpaid, with the International Society of Nephrology and the Western Europe Regional Board, International Society of Nephrology; and other support from Scientific-Tools.org, all outside the submitted work. I Ilic report support for the present manuscript from the Ministry of Education, Science, and Technological Development, Republic of Serbia (Project No. 175042, 2011–2023). M Ilic reports support for the present manuscript from the Ministry of Science, Technological Development, and Innovation, Republic of Serbia (Project No. 451-03-47/2023-01/200111). A-F A Mentis reports grants or contracts from ‘MilkSafe: A novel pipeline to enrich formula milk using omics technologies', a research co financed by the European Regional Development Fund of the European Union and Greek national funds through the Operational Program Competitiveness, Entrepreneurship and Innovation, under the call RESEARCH–CREATE–INNOVATE (project code: T2EDK-02222), as well as from ELIDEK (Hellenic Foundation for Research and Innovation, MIMS-860) (both outside of the present manuscript); payment or expert testimony as a peer-reviewer for FONDAZIONE CARIPLO, ITALY; Participation on a Data Safety Monitoring Board or Advisory Board as Editorial Board Member for “Systematic Reviews” journal, for “Annals of Epidemiology” journal, and as Associate Editor for “Translational Psychiatry”; stock or stock options on a family winery; other financial interests as a scientific officer as part of the BGI Group; outside the submitted work. A Ortiz reports grants to their institute from Sanofi and Catedra Mundipharma-UAM of diabetic kidney disease and the Catedra Astrazeneca-UAM of chronic kidney disease and electrolytes; consultancy or speaker fees Advicciene, Astellas, Astrazeneca, Amicus, Amgen, Fresenius Medical Care, GSK, Bayer, Sanofi-Genzyme, Menarini, Kyowa Kirin, Alexion, Idorsia, Chiesi, Otsuka, Novo-Nordisk and Vifor Fresenius Medical Care Renal Pharma; travel support from Advicciene, Astellas, Astrazeneca, Fresenius Medical Care, Boehringer-Ingelheim Bayer, Sanofi-Genzyme, Menarini, Chiesi, Otsuka, Sysmex; leadership or fiduciary role, unpaid, with Council ERA. SOMANE; all outside the submitted work. J A Singh reports consulting fees from ROMTech, Atheneum, Clearview healthcare partners, American College of Rheumatology, Yale, Hulio, Horizon Pharmaceuticals, DINORA, Frictionless Solutions, Schipher, Crealta/Horizon, Medisys, Fidia, PK Med, Two labs Inc., Adept Field Solutions, Clinical Care options, Putnam associates, Focus forward, Navigant consulting, Spherix, MedIQ, Jupiter Life Science, UBM LLC, Trio Health, Medscape, WebMD, and Practice Point communications; and the National Institutes of Health; Payment or honoraria for lectures, presentations, speakers bureaus, manuscript writing or educational events on the speakers bureau of Simply Speaking; Support for attending meetings and/or travel from OMERACT as a steering committee member; Participation on a Data Safety Monitoring Board or Advisory Board with the FDA Arthritis Advisory Committee; Leadership or fiduciary role in other board, society, committee or advocacy group, paid as a past steering committee member of the OMERACT, an international organization that develops measures for clinical trials and receives arm's length funding from 12 pharmaceutical companies, unpaid as Chair of the Veterans Affairs Rheumatology Field Advisory Committee, and unpaid as the Editor and Director of the UAB Cochrane Musculoskeletal Group Satellite Center on Network Meta-analysis; Stock or stock options in Atai life sciences, Kintara therapeutics, Intelligent Biosolutions, Acumen pharmaceutical, TPT Global Tech, Vaxart pharmaceuticals, Atyu biopharma, Adaptimmune Therapeutics, GeoVax Labs, Pieris Pharmaceuticals, Enzolytics Inc., Seres Therapeutics, Tonix Pharmaceuticals Holding Corp., Aebona Pharmaceuticals, and Charlotte's Web Holdings, Inc. and previously owned stock options in Amarin, Viking, and Moderna Pharmaceuticals; all outside the submitted work. A Zumla reports support for the present manuscript the Pan-African Network for Rapid Research, Response, Relief and Preparedness for Infectious Disease Epidemics (PANDORA-ID-NET) funded by the EDCTP–the EU Horizon 2020 Framework Programme, and is a recipient of the UK NIHR Senior Investigator Award, Mahathir Science Award, and EU-EDCTP Pascoal Mocumbi Prize Laureate.

## Appendix A Supplementary data

Appendix

GBD 2021 Supplementary Methods

Urolithiasis PubMed Table
