# Supplementary material for: The global, regional, and national burden of urolithiasis in 204 countries and territories, 2000–2021: a systematic analysis for the Global Burden of Disease Study 2021
Source: eClinicalMedicine. 2024 Nov 21;78:102924. doi: 10.1016/j.eclinm.2024.102924 (PMC11618031; doi:10.1016/j.eclinm.2024.102924)
Supplement: Appendix [file mmc1.pdf]

Supplementary Appendix 2: Authorship appendix to "The global, regional, and national burden of urolithiasis in 204 countries and territories, 2000–2021: a systematic analysis for the Global Burden of Disease Study 2021"

This appendix provides further authorship detail for "The global, regional, and national burden of urolithiasis in 204 countries and territories, 2000–2021: a systematic analysis for the Global Burden of Disease Study 2021"

Contents

Appendix 2: Authorship appendix to "The global, regional, and national burden of urolithiasis in 204 countries and territories, 2000–2021: a systematic analysis for the Global Burden of Disease Study 2021" 1

GBD 2021 Urolithiasis Collaborators 3

Affiliations 3

Authors' Contributions 5

Developing methods or computational machinery 5

Providing critical feedback on methods or results 5

Drafting the work or revising it critically for important intellectual content 6

Direct access and verified data 6

GBD 2021 Urolithiasis Collaborators

Atalel Fentahun Awedew, Hannah Han, Bétyna N Berice, Maxwell Dodge, Rachel D Schneider, Mohsen Abbasi-Kangevari, Ziyad Al-Aly, Omar Almidani, Saba Alvand, Jalal Arabloo, Aleksandr Y Aravkin, Tegegn Mulatu Ayana, Nikha Bhardwaj, Pankaj Bhardwaj, Sonu Bhaskar, Boris Bikbov, Florentino Luciano Caetano dos Santos, Jaykaran Charan, Natalia Cruz-Martins, Omid Dadras, Xiaochen Dai, Lankamo Ena Digesa, Muhammed Elhadi, Mohamed A Elmonem, Christopher Imokhuede Esezobor, Ali Fatehizadeh, Teferi Gebru Gebremeskel, Motuma Erena Getachew, Seyyed-Hadi Ghamari, Simon I Hay, Irena M Ilic, Milena D Ilic, Umesh Jayarajah, Seyed Behzad Jazayeri, Min Seo Kim, Sang-woong Lee, Shaun Wen Huey Lee, Stephen S Lim, Mansour Adam Mahmoud, Ahmad Azam Malik, Alexios-Fotios A Mentis, Tomislav Mestrovic, Irmina Maria Michalek, Gedefaye Nibret Mihrtie, Erkin M Mirrakhimov, Ali H Mokdad, Mohammad Ali Moni, Maryam Moradi, Christopher J L Murray, Alberto Ortiz, Shrikant Pawar, Norberto Perico, Mohammad-Mahdi Rashidi, Reza Rawassizadeh, Giuseppe Remuzzi, Austin E Schumacher, Jasvinder A Singh, Valentin Yurievich Skryabin, Anna Aleksandrovna Skryabina, Ker-Kan Tan, Musliu Adetola Tolani, Sahel Valadan Tahbaz, Rohollah Valizadeh, Bay Vo, Asrat Arja Wolde, Seyed Hossein Yahyazadeh Jabbari, Fereshteh Yazdanpanah, Arzu Yiğit, Vahit Yiğit, Mazyar Zahir, Michael Zastrozhin, Zhi-Jiang Zhang, Alimuddin Zumla, Awoke Misganaw, and M Ashworth Dirac.

Affiliations

Department of Surgery (A F F Awedew MD), Department of Midwifery (G N Mihrtie MSc), Debre Tabor University, Debre Tabor, Ethiopia; Institute for Health Metrics and Evaluation (H Han MSc, B N Berice MPH, M Dodge BA, R D Schneider MPPM, A Y Aravkin PhD, X Dai PhD, Prof S I Hay FMedSci, Prof S S Lim PhD, T Mestrovic PhD, Prof A H Mokdad PhD, Prof C J L Murray DPhil, A E Schumacher PhD, A A Wolde MPH, M A Dirac MD), Department of Applied Mathematics (A Y Aravkin PhD), Department of Health Metrics Sciences, School of Medicine (A Y Aravkin PhD, X Dai PhD, Prof S I Hay FMedSci, Prof S S Lim PhD, Prof A H Mokdad PhD, Prof C J L Murray DPhil, A Misganaw PhD, M A Dirac MD), Department of Family Medicine (M A Dirac MD), University of Washington, Seattle, WA, USA; Non-communicable Diseases Research Center (M Abbasi-Kangevari MD), Social Determinants of Health Research Center (S Ghamari MD, M Rashidi MD), Shahid Beheshti University of Medical Sciences, Tehran, Iran; Department of Research and Development (Z Al-Aly MD), Washington University in St. Louis, St. Louis, MO, USA; Clinical Epidemiology Center (Z Al-Aly MD), US Department of Veterans Affairs (VA), St. Louis, MO, USA; Department of Urology (O Almidani MSc), Cleveland Clinic Abu Dhabi, Abu Dhabi, United Arab Emirates; Nuffield Department of Surgical Sciences (O Almidani MSc), University of Oxford, Oxford, UK; Liver and Pancreatobiliary Diseases Research Center (S Alvand MD), Iranian Research Center for HIV/AIDS (IRCHA) (O Dadras PhD), Non-communicable Diseases Research Center (S Ghamari MD, M Rashidi MD), Department of Pediatric Allergy and Immunology (F Yazdanpanah MD), Tehran University of Medical Sciences, Tehran, Iran; Health Management and Economics Research Center (J Arabloo PhD), Iran University of Medical Sciences, Tehran, Iran (M Moradi MD); School of Nursing (T M Ayana MSc), Department of Comprehensive Nursing (L E Digesa MSc), Arba Minch University, Arba Minch, Ethiopia; Department of Anatomy (N Bhardwaj MD), Department of Community Medicine and Family Medicine (Prof P Bhardwaj MD), School of Public Health (Prof P Bhardwaj MD), Department of Pharmacology (J Charan MD), All India Institute of Medical Sciences, Jodhpur, India; Global Health Neurology Lab (S Bhaskar MD), NSW Brain Clot Bank, Sydney, NSW, Australia; Division of Cerebrovascular Medicine and Neurology (S Bhaskar MD), National Cerebral and Cardiovascular Center, Suita, Japan; Scientific-Tools.Org,

Bergamo, Italy (B Bikbov MD); Harvard Business School (F Caetano dos Santos PhD), Harvard University, Boston, MA, USA; Department of Diagnostic and Therapeutic Technologies (Prof N Cruz-Martins PhD), Cooperativa de Ensino Superior Politécnico e Universitário (Polytechnic and University Higher Education Cooperative), Vila Nova de Famalicão, Portugal; Institute for Research and Innovation in Health (i3S) (Prof N Cruz-Martins PhD), University of Porto, Porto, Portugal; Department of Global Public Health and Primary Care (O Dadras PhD), University of Bergen, Bergen, Norway; Faculty of Medicine (M Elhadi MD), University of Tripoli, Tripoli, Libya; Houston Methodist Hospital, Houston, TX, USA (M Elhadi MD); Egypt Center for Research and Regenerative Medicine (ECRRM), Cairo, Egypt (M A Elmonem PhD); Department of Paediatrics (C I Esezobor MB), University of Lagos, Lagos, Nigeria; Department of Paediatrics (C I Esezobor MB), Lagos University Teaching Hospital, Lagos, Nigeria; School of Engineering (A Fatehizadeh PhD), Edith Cowan University, Joondalup, WA, Australia; Department of Reproductive and Family Health (T G Gebremeskel PhD), Axum College of Health Science, Axum, Ethiopia; College of Medicine and Public Health (T G Gebremeskel PhD), Flinders University, Adelaide, SA, Australia; Department of Public Health (M E Getachew MPH), Wollega University, Nekemte, Ethiopia; Department of Public Health (M E Getachew MPH), Jimma University, Jimma, Ethiopia; Faculty of Medicine (I M Ilic PhD), University of Belgrade, Belgrade, Serbia; Faculty of Medical Sciences (Prof M D Ilic PhD), University of Kragujevac, Kragujevac, Serbia; Postgraduate Institute of Medicine (U Jayarajah MD), University of Colombo, Colombo, Sri Lanka; Department of Surgery (U Jayarajah MD), National Hospital, Colombo, Sri Lanka; Urology Department (S Jazayeri MD), University of Florida, Jacksonville, FL, USA; Broad Institute of MIT and Harvard, Cambridge, MA, USA (M Kim MD); Massachusetts General Hospital, Boston, MA, USA (M Kim MD); Pattern Recognition and Machine Learning Lab (Prof S Lee PhD), Gachon University, Seongnam, South Korea; School of Pharmacy (S W H Lee PhD), Monash University, Subang Jaya, Malaysia; School of Pharmacy (S W H Lee PhD), Taylor's University Lakeside Campus, Subang Jaya, Malaysia; Department of Clinical and Hospital Pharmacy (M A Mahmoud PhD), Ashok and Rita Patel Institute of Physiotherapy, Al-Madinah Al-Munawarrah, Saudi Arabia; Rabigh Faculty of Medicine (Prof A Malik PhD), King Abdulaziz University, Jeddah, Saudi Arabia; International Dx Department (A A Mentis MD), BGI Genomics, Copenhagen, Denmark; University Centre Varazdin (T Mestrovic PhD), University North, Varazdin, Croatia; National Cancer Registry (I Michalek PhD), Department of Pathology (I Michalek PhD), Maria Sklodowska-Curie National Research Institute of Oncology, Warsaw, Poland; Internal Medicine Programme (Prof E M Mirrakhimov PhD), Kyrgyz State Medical Academy, Bishkek, Kyrgyzstan; Department of Atherosclerosis and Coronary Heart Disease (Prof E M Mirrakhimov PhD), National Center of Cardiology and Internal Disease, Bishkek, Kyrgyzstan; AI & Cyber Futures Institute (M Moni PhD), Charles Sturt University, Bathurst, NSW, Australia; The University of Queensland, Brisbane, QLD, Australia (M Moni PhD); Department of Medicine (Prof A Ortiz MD), Universidad Autónoma de Madrid (Autonomous University of Madrid), Madrid, Spain; Department of Nephrology and Hypertension (Prof A Ortiz MD), The Institute for Health Research Foundation Jiménez Díaz University Hospital, Madrid, Spain; Department of Genetics (S Pawar PhD), Yale University, New Haven, CT, USA; Mario Negri Institute for Pharmacological Research, Bergamo, Italy (N Perico MD, Prof G Remuzzi MD); Department of Computer Science (R Rawassizadeh PhD), Boston University, Boston, MA, USA; School of Medicine (Prof J A Singh MD), Baylor College of Medicine, Houston, TX, USA; Department of Medicine Service (Prof J A Singh MD), US Department of Veterans Affairs (VA), Houston, TX, USA; Clinical Branch (V Y Skryabin MD), Moscow Research and Practical Centre on Addictions, Moscow, Russia; Addiction Psychiatry Department (V Y Skryabin MD), Russian Medical Academy of Continuous Professional Education, Moscow, Russia; Department of Infectious Diseases and Epidemiology (A A Skryabina MD), Pirogov Russian National Research Medical

University, Moscow, Russia; Department of Surgery (K Tan PhD), National University of Singapore, Singapore, Singapore; Department of Surgery (M A Tolani FWACS), Ahmadu Bello University, Zaria, Nigeria; Clinical Cancer Research Center (S Valadan Tahbaz PhD, S Yahyazadeh Jabbari MD), Milad General Hospital, Tehran, Iran; Department of Microbiology (S Valadan Tahbaz PhD), Islamic Azad University, Tehran, Iran; Urmia University of Medical Sciences, Urmia, Iran (R Valizadeh PhD); Faculty of Information Technology (B Vo PhD), HUTECH University, Ho Chi Minh City, Viet Nam; National Data Management Center for Health (NDMC) (A A Wolde MPH), National Data Management Center for Health (A Misganaw PhD), Ethiopian Public Health Institute, Addis Ababa, Ethiopia; Department of Radiology (F Yazdanpanah MD), University of Pennsylvania, Philadelphia, PA, USA; Department of Health Management (A Yiğit PhD, V Yiğit PhD), Süleyman Demirel Üniversitesi (Süleyman Demirel University), Isparta, Türkiye; Norris Comprehensive Cancer Center, Keck School of Medicine (M Zahir MD), Shahid Beheshti University of Medical Sciences, Los Angeles, CA, USA; Department of Bioengineering and Therapeutical Sciences (Prof M Zastrozhin PhD), University of California San Francisco, San Francisco, CA, USA; Department of Administration (Prof M Zastrozhin PhD), PGxAI, San Francisco, CA, USA; School of Public Health (Prof Z Zhang PhD), Wuhan University, Wuhan, China; Center for Clinical Microbiology (Prof A Zumla PhD), University College London, London, UK; NIHR-Biomedical Research Centre (NIHR-BRC) (Prof A Zumla PhD), University College London Hospitals, London, UK.

Authors' Contributions

Providing data or critical feedback on data sources

Omar Almidani, Jalal Arabloo, Atalel Fentahun Awedew, Sonu Bhaskar, Boris Bikbov, Natalia CruzMartins, Xiaochen Dai, M Ashworth Dirac, Ali Fatehizadeh, Teferi Gebru Gebremeskel, Hannah Han, Simon I Hay, Sang-woong Lee, Shaun Wen Huey Lee, Stephen S Lim, Erkin M Mirrakhimov, Awoke Misganaw, Ali H Mokdad, Mohammad Ali Moni, Maryam Moradi, Christopher J L Murray, Alberto Ortiz, Shrikant Pawar, Reza Rawassizadeh, Jasvinder A Singh, Valentin Yurievich Skryabin, Anna Aleksandrovna Skryabina, Ker-Kan Tan, Musliu Adetola Tolani, Sahel Valadan Tahbaz, Bay Vo, Seyed Hossein Yahyazadeh Jabbari, Vahit Yiğit, Michael Zastrozhin, and Alimuddin Zumla.

Developing methods or computational machinery

Aleksandr Y Aravkin, Xiaochen Dai, M Ashworth Dirac, Hannah Han, Simon I Hay, Awoke Misganaw, Ali H Mokdad, Christopher J L Murray, and Austin E Schumacher.

Providing critical feedback on methods or results

Ziyad Al-Aly, Omar Almidani, Saba Alvand, Jalal Arabloo, Atalel Fentahun Awedew, Tegegn Mulatu Ayana, Nikha Bhardwaj, Pankaj Bhardwaj, Sonu Bhaskar, Boris Bikbov, Florentino Luciano Caetano dos Santos, Jaykaran Charan, Natalia Cruz-Martins, Omid Dadras, Xiaochen Dai, Lankamo Ena Digesa, M Ashworth Dirac, Muhammed Elhadi, Mohamed A Elmonem, Ali Fatehizadeh, Teferi Gebru Gebremeskel, Motuma Erena Getachew, Hannah Han, Simon I Hay, Irena M Ilic, Milena D Ilic, Umesh Jayarajah, Seyed Behzad Jazayeri, Min Seo Kim, Shaun Wen Huey Lee, Stephen S Lim, Mansour Adam Mahmoud, Ahmad Azam Malik, Alexios-Fotios A Mentis, Tomislav Mestrovic, Irmina Maria Michalek, Gedefaye Nibret

Mihrtie, Erkin M Mirrakhimov, Awoke Misganaw, Ali H Mokdad, Mohammad Ali Moni, Maryam Moradi, Christopher J L Murray, Shrikant Pawar, Mohammad-Mahdi Rashidi, Reza Rawassizadeh, Jasvinder A Singh, Valentin Yurievich Skryabin, Anna Aleksandrovna Skryabina, Ker-Kan Tan, Sahel Valadan Tahbaz, Rohollah Valizadeh, Bay Vo, Asrat Arja Wolde, Seyed Hossein Yahyazadeh Jabbari, Fereshteh Yazdanpanah, Arzu Yiğit, Mazyar Zahir, and Michael Zastrozhin.

Drafting the work or revising it critically for important intellectual content

Mohsen Abbasi-Kangevari, Omar Almidani, Saba Alvand, Jalal Arabloo, Atalel Fentahun Awedew, Tegegn Mulatu Ayana, Sonu Bhaskar, Boris Bikbov, Florentino Luciano Caetano dos Santos, Natalia Cruz-Martins, M Ashworth Dirac, Muhammed Elhadi, Mohamed A Elmonem, Christopher Imokhuede Esezobor, Ali Fatehizadeh, Seyyed-Hadi Ghamari, Hannah Han, Simon I Hay, Irena M Ilic, Milena D Ilic, Min Seo Kim, Ahmad Azam Malik, Alexios-Fotios A Mentis, Tomislav Mestrovic, Irmina Maria Michalek, Ali H Mokdad, Mohammad Ali Moni, Maryam Moradi, Alberto Ortiz, Shrikant Pawar, Norberto Perico, Giuseppe Remuzzi, Jasvinder A Singh, Valentin Yurievich Skryabin, Anna Aleksandrovna Skryabina, Ker-Kan Tan, Sahel Valadan Tahbaz, Seyed Hossein Yahyazadeh Jabbari, Arzu Yiğit, Vahit Yiğit, Mazyar Zahir, Michae Zastrozhin, Zhi-Jiang Zhang, and Alimuddin Zumla.

Managing the estimation or publications process

Atalel Fentahun Awedew, M Ashworth Dirac, Simon I Hay, Awoke Misganaw, Christopher J L Murray, and Rachel D Schneider

Direct access and verified data

Atalel Fentahun Awedew, M Ashworth Dirac, Simon I Hay, Christopher J L Murray, Hannah Han, Maxwell Dodge, Bétyna N Berice
