# Supplementary material for: The global, regional, and national burden of urolithiasis in 204 countries and territories, 2000–2021: a systematic analysis for the Global Burden of Disease Study 2021
Source: eClinicalMedicine. 2024 Nov 21;78:102924. doi: 10.1016/j.eclinm.2024.102924 (PMC11618031; doi:10.1016/j.eclinm.2024.102924)
Supplement: GBD 2021 Supplementary Methods [file mmc2.docx]

**Supplementary Appendix 1:** Supplementary Methods to: “The global, regional, and national burden of urolithiasis in 204 countries and territories, 2000–2021: a systematic analysis for the Global Burden of Disease Study 2021”. GBD 2021 Urolithiasis Collaborators.

# Section 1. Statement of GATHER compliance

This study complies with the Guideline for Accurate and Transparent Health Estimates Reporting (GATHER) recommendations. See table S1 below for the GATHER checklist. The GATHER recommendations can be found on the GATHER website (<https://www.who.int/data/gather>).

### Table S1. Guidelines for Accurate and Transparent Health Estimates Reporting (GATHER) checklist

| Item # | Checklist item | Reporting location |
| --- | --- | --- |
| Objectives and funding | | |
| 1 | Define the indicator(s), populations (including age, sex, and geographic entities), and time period(s) for which estimates were made. | Supplementary Methods Section 2 |
| 2 | List the funding sources for the work. | Page 3, line 65 |
| Data Inputs | | |
| For all data inputs from multiple sources that are synthesized as part of the study: | | |
| 3 | Describe how the data were identified and how the data were accessed. | Page 6, line 142-155  Pages 7-8 lines 164-211 |
| 4 | Specify the inclusion and exclusion criteria. Identify all ad-hoc exclusions. | Page 8, 199-202 |
| 5 | Provide information on all included data sources and their main characteristics. For each data source used, report reference information or contact name/institution, population represented, data collection method, year(s) of data collection, sex and age range, diagnostic criteria or measurement method, and sample size, as relevant. | https://ghdx.healthdata.org/ |
| 6 | Identify and describe any categories of input data that have potentially important biases (e.g., based on characteristics listed in item 5). | Page 7-8, lines 180-197  Page 23, lines 623-637 |
| For data inputs that contribute to the analysis but were not synthesized as part of the study: | | |
| 7 | Describe and give sources for any other data inputs. | <https://ghdx.healthdata.org/>; Acute urolithiasis nonfatal model can be viewed, and when set to “Unadjusted” with Outliers “On” will show all data considered for inclusion in the model, as extracted and prior to adjustment for biased population sample due to commercial health insurance states. “Adjusted” with Outliers “Off” will show only data used in the model, and will show those from commercial insurance databases as adjusted.  <http://ihmeuw.org/6j6x>: Urolithiasis model will show data considered for inclusion but excluded marked in red as “Outliers”. |
| For all data inputs: | | |
| 8 | Provide all data inputs in a file format from which data can be efficiently extracted (e.g., a spreadsheet rather than a PDF), including all relevant meta-data listed in item 5. For any data inputs that cannot be shared because of ethical or legal reasons, such as third-party ownership, provide a contact name or the name of the institution that retains the right to the data. | https://ghdx.healthdata.org/ |
| Data analysis | | |
| 9 | Provide a conceptual overview of the data analysis method. A diagram may be helpful. | Page 6-8, lines 133-238 |
| 10 | Provide a detailed description of all steps of the analysis, including mathematical formulae. This description should cover, as relevant, data cleaning, data pre-processing, data adjustments and weighting of data sources, and mathematical or statistical model(s). | Page 6-8, lines 133-238  Supplementary Methods Appendix Section 4 |
| 11 | Describe how candidate models were evaluated and how the final model(s) were selected. | Page 6-8, lines 142-211  Supplementary Methods Appendix Section 4 |
| 12 | Provide the results of an evaluation of model performance, if done, as well as the results of any relevant sensitivity analysis. | Supplementary Methods Appendix Section 5 |
| 13 | Describe methods for calculating uncertainty of the estimates. State which sources of uncertainty were, and were not, accounted for in the uncertainty analysis. | Page 9, lines 220-238 |
| 14 | State how analytic or statistical source code used to generate estimates can be accessed. | <https://ghdx.healthdata.org/gbd-2021/code> |
| Results and Discussion | | |
| 15 | Provide published estimates in a file format from which data can be efficiently extracted. | https://ghdx.healthdata.org/ |
| 16 | Report a quantitative measure of the uncertainty of the estimates (e.g. uncertainty intervals). | All quantitative findings are reported with point estimates and uncertainty intervals throughout Results, Tables and Figures. |
| 17 | Interpret results in light of existing evidence. If updating a previous set of estimates, describe the reasons for changes in estimates. | Page 5, lines 115-122 |
| 18 | Discuss limitations of the estimates. Include a discussion of any modelling assumptions or data limitations that affect interpretation of the estimates. | Pages 23-23, lines 623-647 |

Section 2. Demographics estimated in the Global Burden of Disease Study

In GBD, we estimated the burden of urolithiasis for age groups between 1 and 99+ years, for males and females, between 1990 and 2021, for 204 countries and territories, and for subnational locations within a subset of countries. Country-level results are reported in this manuscript. A list of countries and how they fit into the GBD geographical hierarchy is shown in below. Countries marked with * are estimated at a subnational level; subnational estimates can be accessed via GBD Compare (<http://ihmeuw.org/61lw>), GBD Results Tool (<https://vizhub.healthdata.org/gbd-results/>), and model-specific visualisations (Epi Visualization <http://ihmeuw.org/6j6x> and CoD Visualization <http://ihmeuw.org/6j6x> ).

### Table S2. Geographical hierarchy

| **Super-region** | **Region** | **Country or territory** |
| --- | --- | --- |
| Central Europe, eastern Europe, and central Asia | Central Asia | Armenia, Azerbaijan, Georgia, Kazakhstan, Kyrgyzstan, Mongolia, Tajikistan, Turkmenistan, Uzbekistan |
|  | Central Europe | Albania, Bosnia and Herzegovina, Bulgaria, Croatia, Czech Republic, Hungary, Montenegro, North Macedonia, Poland*, Romania, Serbia, Slovakia, Slovenia |
|  | Eastern Europe | Belarus, Estonia, Latvia, Lithuania, Moldova, Russia*, Ukraine |
| High-income | Australasia | Australia, New Zealand* (subnational Māori + non-Māori) |
|  | High-income Asia Pacific | Brunei, Japan*, Singapore, South Korea |
|  | High-income North America | Canada, Greenland, United States* |
|  | Southern Latin America | Argentina, Chile, Uruguay |
|  | Western Europe | Andorra, Austria, Belgium, Cyprus, Denmark, Finland, France, Germany, Greece, Iceland, Ireland, Israel, Italy*, Luxembourg, Malta, Monaco, Netherlands, Norway*, Portugal, San Marino, Spain, Switzerland, Sweden*, United Kingdom* |
| Latin America and Caribbean | Andean Latin America | Bolivia, Ecuador, Peru |
|  | Caribbean | Antigua and Barbuda, Bahamas, Barbados, Belize, Bermuda, Cuba, Dominica, Dominican Republic, Grenada, Guyana, Haiti, Jamaica, Puerto Rico, Saint Kitts and Nevis, Saint Lucia, Saint Vincent and the Grenadines, Suriname, Trinidad and Tobago, Virgin Islands |
|  | Central Latin America | Colombia, Costa Rica, El Salvador, Guatemala, Honduras, Mexico*, Nicaragua, Panama, Venezuela |
|  | Tropical Latin America | Brazil*, Paraguay |
| North Africa and Middle East | North Africa and Middle East | Afghanistan, Algeria, Bahrain, Egypt, Iran*, Iraq, Jordan, Kuwait, Lebanon, Libya, Morocco, Oman, Palestine, Qatar, Saudi Arabia, Sudan, Syria, Tunisia, Türkiye, United Arab Emirates, Yemen |
| South Asia | South Asia | Bangladesh, Bhutan, India*, Nepal, Pakistan* |
| Southeast Asia, east Asia, Oceania | East Asia | China, North Korea, Taiwan (province of China) |
|  | Oceania | American Samoa, Cook Islands, Federated States of Micronesia, Fiji, Guam, Kiribati, Marshall Islands, Nauru, Niue, Northern Mariana Islands, Palau, Papua New Guinea, Samoa, Solomon Islands, Tokelau, Tonga, Tuvalu, Vanuatu |
|  | Southeast Asia | Cambodia, Indonesia*, Laos, Malaysia, Maldives, Mauritius, Myanmar, Philippines*, Seychelles, Sri Lanka, Thailand, Timor-Leste, Viet Nam |
| Sub-Saharan Africa | Central sub-Saharan Africa | Angola, Central African Republic, Congo (Brazzaville), Democratic Republic of the Congo, Equatorial Guinea, Gabon |
|  | Eastern sub-Saharan Africa | Burundi, Comoros, Djibouti, Eritrea, Ethiopia*, Kenya*, Madagascar, Malawi, Mozambique, Rwanda, Somalia, South Sudan, Tanzania, Uganda, Zambia |
|  | Southern sub-Saharan Africa | Botswana, Eswatini, Lesotho, Namibia, South Africa*, Zimbabwe |
|  | Western sub-Saharan Africa | Benin, Burkina Faso, Cape Verde, Cameroon, Chad, Côte d’Ivoire, Gambia, Ghana, Guinea, Guinea-Bissau, Liberia, Mali, Mauritania, Niger, Nigeria*, São Tomé and Príncipe, Senegal, Sierra Leone, Togo |

# Section 3. Standard population in the Global Burden of Disease Study

Per GBD standards, age-standardisation was performed using the direct method, as described by Ahmad and colleagues for WHO in 2001.^1^ That is to say, we aggregated year-age-sex-location-specific estimates to the age-sex distribution of a reference population, specifically, the GBD world population age standard. This world population age standard uses the non-weighted mean of GBD 2021’s age-specific population proportional distributions for all national locations with a population greater than 5 million people in 2019 (non-pandemic year). (1) The population data sources, methods, and estimates have been previously described.(1)

Section 4. Causes of Death Ensemble Model (CODEm) for Urolithiasis
To estimate urolithiasis mortality, the GBD causes of death (CoD) database and a standard causes of death ensemble model (CODEm) with location-level covariates were used. The CoD database comprises vital registration, verbal autopsy, and other sources of data on the causes of deaths in populations, and is described in detail in the supplementary methods appendix to “Global burden of 288 causes of death and life expectancy decomposition in 204 countries and territories and 811 subnational locations, 1990–2021: a systematic analysis for the Global Burden of Disease Study 2021”.(2) CODEm, which uses an ensemble modelling method that involves generation and validation of submodels using the train-test 1-test 2 approach, weighting and testing of the model performance to select the best ensemble model with the highest out-of-sample predictive validity, has originally been described in a peer-reviewed publication by Foreman et al. The updates to the method have been described in a series of publications reporting GBD mortality estimates, most recently the GBD 2021 Causes of Death capstone cited above. (3)

As described in greater detail in Foreman et al, CODEm modelling starts with generation of a diverse family of potential submodels (i.e., component models) in four families: linear mixed effects regression (LMER) models of the natural log of the cause-specific death rate, LMER models of the logit of the cause fraction, spatiotemporal Gaussian process regression (ST-GPR) models of the natural logarithm of the cause-specific death rate, and ST-GPR models of the logit of the cause fraction. In any given application, the analyst specifies all plausible covariates associated with a particular cause of death. For each covariate identified, the analyst specifies the expected direction (positive or negative) of its relationship to cause of death based on scientific evidence and classifies it into one of the three levels (Levels 1, 2, and 3) based on the plausibility and strength of evidence for causal association. The level of each indicator represents the level of association between the covariate and urolithiasis mortality. It ranges from 1, indicating a strong biological link to outcome, to 3, indicating weak or unknown relationship to outcome. The direction of each indicator represents the expected direction of relationship to cause of death.

For urolithiasis, we included the predictive covariates shown in Table SX, which were modelled and estimated as part of GBD.

# Table S4. Covariates used in urolithiasis mortality modelling

| **Level** | **Covariate** | **Direction** |
| --- | --- | --- |
| 1 | Temperature (90^th^ percentile) | + |
|  | Red meat consumption (unadjusted, kcal per capita) | + |
| 2 | Fruit consumption (unadjusted, kcal per capita) | - |
|  | Vegetable consumption (unadjusted, kcal per capita) | - |
|  | Healthcare Access and Quality Index | - |
| 3 | Socio-demographic Index | - |
|  | Education (years per capita) | - |
|  | Log LDI ($I per capita) | - |

Combinations of these covariates are tested for performance in submodels. The linear mixed effects submodels include fixed effects on covariates and age group and nested random effects on super-region, region, country, and age, and can be represented by:

$$ln\left( {rate}_{s,r,c,y,a} \right)=\sum_{i} \beta_{i}X_{i_{s,r,c,y,a}}+\beta_{a}+\pi_{s}+\pi_{s,r}+\pi_{s,r,a}+\pi_{s,r,a,c}+\varepsilon_{s,r,c,y,a}$$

$$logit\left( {cause fraction}_{s,r,c,y,a} \right)=\sum_{i} \beta_{i}X_{i_{s,r,c,y,a}}+\beta_{a}+\pi_{s}+\pi_{s,r}+\pi_{s,r,a}+\pi_{s,r,a,c}+\varepsilon_{s,r,c,y,a}$$

Where:

$s$ = super-region index; $r$= region index; $c$= country index; $y$= year index; $a$= age index; [countries are nested within regions, which are nested within super-regions

$\beta_{i}$ = coefficient on covariate i

$X_{i_{s,r,c,y,a}}$ = covariate i for observation s, r, c, y, a

$\beta_{a}$ = age-specific intercept

$\pi_{s}$ = random intercept on super-region

$\pi_{s,r}$ = random intercept on region (nested within super-region)

$\pi_{s,r,a}$ = random intercept on age (nested within region)

$\pi_{s,r,a,c}$ = random intercept on country (nested within region-age)

$$\varepsilon_{s,r,c,y,a} \sim N(0,\sigma_{\varepsilon}\cdot I)$$

The ST-GPR models begin with a linear prediction, per above, and then smooth over space, time, and age based on weighted residuals of adjacent data and then apply Gaussian process regression.

The process of covariate selection proceeds by level. We test submodels corresponding to all 2*n* - 1 combinations of level 1 covariates, where *n* is the number of covariates in level 1. All submodels where the coefficients for all covariates have the expected sign and are significant at the p <0.05 level are retained. For each level 1 model that was retained, we create a list of 2*m* possible level 2 models (where *m* is the number of level 2 covariates). The first model, which has no level 2 covariates included, has already been tested and is retained. Next, each of the *m* possible models in which one covariate is added to the level 1 model is tested. If adding the level 2 covariate does not affect either the significance or the sign on any level 1 coefficients, and the level 2 covariate itself meets the criteria of direction and significance, then it is retained as another possible submodel. If the level 2 covariate does not fulfill the criteria or forces any of the level 1 covariates to violate their criteria, then the submodel is dropped; all other possible level 2 submodels that contain that covariate are also dropped. Next, we take each of the models resulting from the level 2 process and use the same process as described for level 2 on the level 3 covariates. Ultimately, we obtain a set of all possible covariate combinations that fulfill our expectations for covariate direction. We run the covariate selection for submodels using both logit cause fraction and natural log of cause-specific death rate and then create both LMER-only and ST-GPR models for each set of chosen covariates.

Submodels are fit on 70% of CoD data, with 30% holdouts of the data selected to mimic observed patterns of missingness for age groups, years, and locations. Out-of-sample predictive validity of each submodel is then tested using half of the excluded data (15% of the total). Performance tests include the root-mean-squared-error (RMSE) for the log of the cause-specific death rate, the direction of the predicted versus actual trend in the data, and the coverage of the predicted 95% uncertainty interval. The process of fitting submodels and calculating out-of-sample performance is repeated 20 times. Submodels are ranked according to their performance on these out-of-sample performance tests across the 20 repetitions. Submodels are then weighted to determine their contribution to the ensemble estimate. The relative weights are determined both by the submodel performance ranks and by a parameter ψ, whose value determines how quickly the weights taper off as rank decreases. Specifically, the relationship between weight and parameter ψ is specified using a monotonically decreasing function, given by,


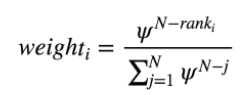


The analyst may specify the range of ψ values to test in order to identify the best ψ value based in the cross-validation.

A set of ensemble models is then created by using the weights constructed from the combinations of ranks and ψ values. These ensembles are tested by using the predictive validity metrics described above on the remaining 15% of the data, and the ensemble with the best performance in out-of-sample trend and RMSE is chosen as the final model.

There are several model parameters that the analyst can set in CODEm, including the size of the data hold-outs, the maximum and minimum values for ψ, the linear floor rate (the lowest death rate per 100,000 in the linear predictor), as well as lambda, zeta, omega and GPR parameters.

The entire ensemble modelling approach is conducted twice for GBD: once to fit a global CODEm model that uses data from, and makes predictions for, all locations in the GBD estimation framework; and once to fit a data-rich CODEm model, which uses data from, and makes predictions for, only the locations considered to be data-rich. The designation of data-rich locations is based on a “star” rating system (0­–5 stars) to rate the quality of data for any given location and year, with 5 being the best and 0 being the worst. The inputs that determine this star rating are the percentage of total deaths determined to be garbage coded (such as “All, Ill-defined”), the percentage of deaths determined to be an aggregated cause, and the level of completeness in the dataset. More detailed information about the causes of death data star rating calculation can be found on pages 45-48 of the Supplementary document of the GBD 2019 publication.^4^

We assumed that children under 1 year of age do not die from urolithiasis; we kept the age restrictions for mortality estimation of urolithiasis of 12 months for lower bound and 95+ years for upper bound. Separate models were conducted for male and female mortality in addition to the global and data-rich locations as mentioned above. We hybridised separate sex-specific global and data-rich models to acquire unadjusted results, which we adjusted using the cause of death correction (CoDCorrect) procedure and compared to the reference life table to calculate final YLLs due to urolithiasis. (2)

### Table S5. Summary statistical metrics on ensemble model performance of urolithiasis

|  | Out-of-sample RMSE | Coverage | Test of trends | Mean error |
| --- | --- | --- | --- | --- |
| Male, global | 0.6559 | 0.9919 | 0.2155 | 6.2217 |
| Male, data-rich | 0.3738 | 0.9994 | 0.1648 | 0.6854 |
| Female, global | 0.6429 | 0.9907 | 0.2188 | 5.5882 |
| Female, data-rich | 0.3406 | 0.9994 | 0.1572 | 0.7061 |

Table S6: Global, super-region, and country-level deaths of acute urolithiasis for both sexes and all locations, and percentage change in 2000 and 2021

| **location_name** | **count.2000** | **rate.2000** | **count.2021** | **rate.2021** | **Percentage change in counts between 2000 and 2021** | **Percentage change in age-standardized rates between 2000 and 2021** |
| --- | --- | --- | --- | --- | --- | --- |
| **Global** | **11000 (7950–12800)** | **0·232 (0·167–0·269)** | **17700 (13900–21200)** | **0·212 (0·168–0·254)** | **60·3 (41·1–87·5)** | **-8·55 (-19·1–6·75)** |
| **Central Europe, Eastern Europe, and Central Asia** | **1800 (1720–1930)** | **0·355 (0·339–0·381)** | **2640 (2400–2980)** | **0·400 (0·363–0·453)** | **46·4 (34·2–63·4)** | **12·5 (3·52–25·9)** |
| Central Asia | 147 (115–188) | 0·296 (0·233–0·382) | 269 (218–339) | 0·386 (0·313–0·483) | 83·4 (32·3–155) | 30·6 (-5·66–79·9) |
| Armenia | 16·5 (12·5–24·5) | 0·587 (0·450–0·860) | 21·9 (17·5–28·0) | 0·505 (0·401–0·647) | 32·5 (-7·61–88·0) | -14·0 (-39·3–21·4) |
| Azerbaijan | 5·67 (3·35–8·96) | 0·106 (0·0596–0·169) | 10·1 (4·92–19·4) | 0·134 (0·0630–0·249) | 77·6 (-5·88–278) | 27·3 (-32·5–180) |
| Georgia | 4·58 (3·79–5·40) | 0·0736 (0·0612–0·0863) | 6·74 (5·37–8·46) | 0·110 (0·0883–0·139) | 47·3 (17·8–86·6) | 49·7 (20·4–90·9) |
| Kazakhstan | 97·7 (65·8–136) | 0·811 (0·551–1·13) | 199 (150–265) | 1·29 (0·954–1·70) | 104 (27·9–221) | 58·6 (-1·29–149) |
| Kyrgyzstan | 5·90 (4·80–7·23) | 0·186 (0·152–0·228) | 7·75 (6·10–9·85) | 0·182 (0·142–0·231) | 31·5 (-4·19–74·1) | -1·97 (-29·7–29·7) |
| Mongolia | 1·92 (1·10–3·16) | 0·178 (0·101–0·296) | 1·82 (1·13–3·09) | 0·0978 (0·0575–0·169) | -4·93 (-47·7–82·0) | -45·2 (-70·3–10·1) |
| Tajikistan | 12·6 (7·76–19·1) | 0·447 (0·265–0·724) | 17·0 (9·86–27·8) | 0·378 (0·216–0·618) | 34·5 (-28·2–136) | -15·5 (-55·6–55·6) |
| Turkmenistan | 1·89 (1·35–2·56) | 0·0879 (0·0614–0·121) | 4·74 (2·32–7·23) | 0·136 (0·0685–0·208) | 150 (12·0–306) | 54·8 (-31·0–155) |
| Uzbekistan | 0·0466 (0·0314–0·0937) | 0·000346 (0·000231–0·000704) | 0·0869 (0·0652–0·115) | 0·000394 (0·000289–0·000536) | 86·3 (-8·55–198) | 14·0 (-43·4–84·9) |
| Central Europe | 219 (199–237) | 0·136 (0·124–0·147) | 163 (138–202) | 0·0712 (0·0600–0·0894) | -25·5 (-38·1–-10·5) | -47·6 (-56·6–-36·1) |
| Albania | 1·57 (0·852–2·64) | 0·0648 (0·0356–0·107) | 2·02 (1·05–3·86) | 0·0474 (0·0248–0·0878) | 28·6 (-31·7–128) | -26·8 (-61·3–28·7) |
| Bosnia and Herzegovina | 7·69 (4·55–12·5) | 0·156 (0·0932–0·253) | 5·27 (2·72–10·7) | 0·0833 (0·0434–0·167) | -31·5 (-69·4–23·9) | -46·8 (-75·6–-5·44) |
| Bulgaria | 29·9 (25·2–35·8) | 0·247 (0·209–0·292) | 17·3 (12·3–23·9) | 0·124 (0·0883–0·171) | -42·3 (-60·0–-17·4) | -49·9 (-65·5–-27·9) |
| Croatia | 4·37 (3·49–5·37) | 0·0647 (0·0525–0·0785) | 3·82 (2·86–5·25) | 0·0391 (0·0289–0·0546) | -12·6 (-37·4–21·6) | -39·6 (-56·8–-15·4) |
| Czechia | 29·7 (25·1–34·8) | 0·197 (0·167–0·231) | 21·5 (17·2–27·5) | 0·0953 (0·0757–0·126) | -27·6 (-43·7–-6·84) | -51·7 (-63·0–-36·3) |
| Hungary | 70·2 (60·2–81·6) | 0·452 (0·390–0·526) | 52·8 (42·2–67·3) | 0·258 (0·206–0·331) | -24·9 (-41·8–-2·38) | -43·1 (-55·7–-26·4) |
| Montenegro | 0·170 (0·0919–0·268) | 0·0230 (0·0124–0·0365) | 0·184 (0·0906–0·302) | 0·0208 (0·0102–0·0349) | 8·47 (-45·0–74·0) | -9·76 (-55·1–45·5) |
| North Macedonia | 0·407 (0·242–0·538) | 0·0236 (0·0142–0·0319) | 0·308 (0·190–0·524) | 0·0115 (0·00711–0·0184) | -24·2 (-56·8–24·5) | -51·3 (-72·0–-21·8) |
| Poland | 51·3 (47·9–54·7) | 0·105 (0·0983–0·112) | 39·0 (32·6–50·9) | 0·0537 (0·0445–0·0714) | -23·9 (-36·7–-3·43) | -49·0 (-57·9–-33·7) |
| Romania | 0·471 (0·384–0·558) | 0·00163 (0·00134–0·00193) | 0·369 (0·269–0·509) | 0·000947 (0·000692–0·00131) | -21·6 (-44·2–10·4) | -41·9 (-59·1–-16·7) |
| Serbia | 9·87 (6·05–13·1) | 0·0828 (0·0510–0·110) | 9·48 (6·07–14·6) | 0·0554 (0·0361–0·0853) | -3·96 (-36·9–46·5) | -33·2 (-55·8–1·68) |
| Slovakia | 7·87 (4·89–10·6) | 0·118 (0·0730–0·160) | 6·59 (4·09–11·5) | 0·0691 (0·0427–0·119) | -16·3 (-50·4–38·5) | -41·4 (-65·0–-3·02) |
| Slovenia | 2·07 (1·79–2·39) | 0·0710 (0·0614–0·0826) | 2·02 (1·43–3·21) | 0·0409 (0·0288–0·0664) | -2·40 (-30·0–57·6) | -42·4 (-58·9–-4·52) |
| Eastern Europe | 1440 (1370–1530) | 0·491 (0·468–0·522) | 2210 (1980–2550) | 0·621 (0·556–0·718) | 53·6 (39·3–73·0) | 26·4 (14·9–42·4) |
| Belarus | 88·2 (69·7–111) | 0·636 (0·502–0·801) | 115 (89·5–143) | 0·711 (0·551–0·883) | 30·6 (-6·36–78·5) | 11·8 (-20·7–52·5) |
| Estonia | 8·76 (7·61–10·1) | 0·415 (0·362–0·474) | 12·7 (10·1–15·8) | 0·428 (0·340–0·535) | 44·7 (13·2–84·9) | 3·16 (-19·6–33·1) |
| Latvia | 25·3 (22·2–28·4) | 0·692 (0·609–0·779) | 28·6 (22·9–36·8) | 0·678 (0·536–0·881) | 13·3 (-11·3–42·4) | -2·02 (-23·5–23·9) |
| Lithuania | 25·9 (23·1–29·0) | 0·525 (0·469–0·586) | 35·5 (28·1–46·2) | 0·580 (0·453–0·777) | 37·3 (8·91–75·6) | 10·5 (-13·3–45·3) |
| Republic of Moldova | 17·6 (15·5–19·8) | 0·379 (0·335–0·425) | 26·3 (21·9–32·0) | 0·439 (0·364–0·534) | 49·5 (19·2–86·9) | 15·9 (-7·29–45·7) |
| Russian Federation | 1110 (1070–1160) | 0·576 (0·552–0·599) | 1860 (1670–2160) | 0·776 (0·696–0·902) | 67·4 (50·9–89·7) | 34·7 (21·7–52·9) |
| Ukraine | 158 (133–199) | 0·228 (0·192–0·286) | 126 (83·9–192) | 0·166 (0·110–0·257) | -20·4 (-43·7–13·8) | -27·1 (-48·7–4·89) |
| **High-income** | **1410 (1270–1500)** | **0·0933 (0·0841–0·0987)** | **3340 (2790–3770)** | **0·130 (0·111–0·145)** | **136 (116–154)** | **38·9 (30·0–49·1)** |
| Australasia | 35·4 (31·1–39·4) | 0·114 (0·100–0·127) | 80·7 (69·2–92·0) | 0·138 (0·119–0·156) | 128 (94·0–165) | 20·8 (3·41–39·8) |
| Australia | 26·3 (23·0–29·6) | 0·101 (0·0888–0·114) | 64·2 (54·6–74·0) | 0·128 (0·110–0·146) | 144 (105–186) | 26·2 (7·35–47·8) |
| New Zealand | 9·00 (7·97–10·1) | 0·178 (0·158–0·200) | 16·5 (14·2–18·6) | 0·189 (0·165–0·211) | 83·0 (58·1–115) | 6·07 (-7·93–24·0) |
| High-income Asia Pacific | 228 (194–250) | 0·0833 (0·0710–0·0914) | 1070 (812–1260) | 0·160 (0·127–0·185) | 371 (304–419) | 92·1 (71·7–109) |
| Brunei Darussalam | 0·229 (0·157–0·342) | 0·200 (0·134–0·302) | 0·536 (0·315–0·761) | 0·237 (0·139–0·337) | 134 (59·1–270) | 18·8 (-20·2–88·5) |
| Japan | 199 (170–217) | 0·0837 (0·0715–0·0910) | 999 (747–1170) | 0·180 (0·144–0·205) | 401 (333–446) | 115 (97·2–131) |
| Republic of Korea | 25·1 (17·8–37·3) | 0·0850 (0·0586–0·128) | 61·9 (24·4–106) | 0·0688 (0·0272–0·118) | 147 (5·96–318) | -19·0 (-64·2–39·7) |
| Singapore | 2·86 (2·54–3·16) | 0·103 (0·0913–0·115) | 9·95 (8·01–12·0) | 0·122 (0·0984–0·147) | 248 (183–321) | 18·5 (-2·53–43·3) |
| High-income North America | 376 (334–401) | 0·0852 (0·0764–0·0907) | 1030 (894–1140) | 0·150 (0·132–0·164) | 175 (157–195) | 76·2 (65·1–88·8) |
| Canada | 34·9 (30·5–38·8) | 0·0821 (0·0719–0·0912) | 112 (92·1–131) | 0·140 (0·117–0·164) | 219 (166–278) | 70·9 (43·8–102) |
| Greenland | 0·00988 (0·00368–0·0145) | 0·0370 (0·00931–0·0583) | 0·0176 (0·00390–0·0304) | 0·0373 (0·00638–0·0640) | 78·6 (-7·75–194) | 0·707 (-42·6–64·2) |
| United States of America | 341 (302–364) | 0·0857 (0·0765–0·0913) | 921 (800–1020) | 0·151 (0·133–0·167) | 170 (153–191) | 76·6 (65·8–89·9) |
| Southern Latin America | 31·2 (28·4–34·1) | 0·0559 (0·0508–0·0609) | 43·9 (38·9–49·2) | 0·0499 (0·0444–0·0559) | 40·5 (23·4–61·9) | -10·8 (-21·5–2·77) |
| Argentina | 14·2 (12·6–16·0) | 0·0371 (0·0331–0·0415) | 17·7 (15·2–20·3) | 0·0312 (0·0271–0·0360) | 24·6 (3·31–52·9) | -15·8 (-30·1–2·62) |
| Chile | 12·5 (11·3–13·7) | 0·0949 (0·0854–0·104) | 19·9 (17·1–23·0) | 0·0781 (0·0672–0·0902) | 59·0 (35·8–87·6) | -17·7 (-29·6–-2·80) |
| Uruguay | 4·52 (4·04–4·99) | 0·103 (0·0924–0·114) | 6·30 (5·41–7·30) | 0·110 (0·0947–0·127) | 39·4 (16·8–67·2) | 6·21 (-10·6–26·9) |
| Western Europe | 743 (675–792) | 0·105 (0·0956–0·111) | 1110 (948–1270) | 0·101 (0·0878–0·115) | 49·6 (37·1–64·9) | -3·81 (-11·5–6·48) |
| Andorra | 0·101 (0·0491–0·172) | 0·112 (0·0547–0·192) | 0·164 (0·0707–0·272) | 0·0970 (0·0418–0·165) | 62·5 (-1·40–171) | -13·5 (-48·5–44·0) |
| Austria | 21·1 (18·5–23·7) | 0·152 (0·133–0·169) | 39·3 (32·8–46·1) | 0·184 (0·154–0·214) | 86·0 (56·4–121) | 21·2 (2·89–43·4) |
| Belgium | 19·2 (16·6–21·8) | 0·104 (0·0903–0·117) | 24·9 (20·0–29·2) | 0·0903 (0·0749–0·106) | 29·7 (7·70–55·0) | -12·9 (-26·5–3·32) |
| Cyprus | 2·35 (1·53–3·75) | 0·281 (0·179–0·443) | 2·80 (1·74–4·46) | 0·174 (0·104–0·306) | 19·2 (-25·8–103) | -38·1 (-62·8–8·24) |
| Denmark | 18·8 (16·7–21·0) | 0·204 (0·182–0·227) | 25·5 (21·8–29·8) | 0·192 (0·166–0·224) | 35·5 (16·7–57·7) | -5·75 (-18·6–9·34) |
| Finland | 6·40 (5·46–7·24) | 0·0736 (0·0632–0·0828) | 10·8 (8·86–13·0) | 0·0737 (0·0616–0·0874) | 69·0 (43·2–95·7) | 0·0677 (-14·7–15·7) |
| France | 81·6 (71·7–90·8) | 0·0791 (0·0700–0·0879) | 114 (92·3–135) | 0·0648 (0·0537–0·0768) | 39·7 (18·7–63·5) | -18·1 (-29·8–-5·26) |
| Germany | 96·5 (84·7–109) | 0·0635 (0·0563–0·0715) | 187 (149–226) | 0·0818 (0·0661–0·0975) | 94·1 (58·2–132) | 28·7 (5·98–52·8) |
| Greece | 1·56 (1·36–1·78) | 0·00881 (0·00769–0·0100) | 2·98 (2·45–3·49) | 0·00998 (0·00847–0·0116) | 90·9 (54·7–133) | 13·2 (-7·95–36·1) |
| Iceland | 0·341 (0·291–0·385) | 0·0926 (0·0793–0·105) | 0·852 (0·701–1·00) | 0·129 (0·108–0·152) | 150 (109–198) | 39·4 (17·3–66·9) |
| Ireland | 4·34 (3·89–4·83) | 0·0925 (0·0831–0·103) | 5·62 (4·62–6·67) | 0·0672 (0·0556–0·0794) | 29·5 (6·46–54·2) | -27·3 (-39·8–-14·1) |
| Israel | 14·5 (12·6–16·2) | 0·216 (0·188–0·242) | 24·1 (19·3–28·7) | 0·177 (0·143–0·211) | 66·6 (38·5–98·2) | -18·2 (-31·8–-3·21) |
| Italy | 153 (134–165) | 0·135 (0·119–0·146) | 147 (120–178) | 0·0807 (0·0665–0·101) | -3·73 (-14·5–12·8) | -40·2 (-47·3–-28·0) |
| Luxembourg | 0·368 (0·325–0·411) | 0·0574 (0·0506–0·0641) | 0·611 (0·519–0·713) | 0·0520 (0·0443–0·0613) | 66·0 (40·8–94·2) | -9·42 (-23·3–7·13) |
| Malta | 0·661 (0·585–0·743) | 0·122 (0·108–0·137) | 1·17 (0·976–1·36) | 0·109 (0·0922–0·126) | 76·3 (48·6–107) | -11·1 (-25·1–4·49) |
| Monaco | 0·0148 (0·00839–0·0220) | 0·0164 (0·00937–0·0241) | 0·0192 (0·00972–0·0320) | 0·0172 (0·00885–0·0275) | 29·4 (-14·0–115) | 4·88 (-29·6–69·3) |
| Netherlands | 51·5 (45·0–57·3) | 0·212 (0·186–0·235) | 74·2 (63·2–86·4) | 0·192 (0·165–0·224) | 44·1 (24·0–67·7) | -9·39 (-21·5–5·41) |
| Norway | 12·6 (11·1–13·7) | 0·145 (0·128–0·157) | 19·8 (17·2–22·1) | 0·168 (0·148–0·188) | 57·0 (41·2–73·9) | 16·2 (4·40–28·6) |
| Portugal | 13·4 (11·9–14·8) | 0·0828 (0·0736–0·0914) | 20·9 (17·0–25·0) | 0·0715 (0·0595–0·0852) | 56·3 (29·5–85·1) | -13·5 (-27·1–2·03) |
| San Marino | 0·0761 (0·0453–0·114) | 0·125 (0·0779–0·190) | 0·0743 (0·0405–0·121) | 0·0753 (0·0416–0·122) | -2·35 (-41·2–55·7) | -39·8 (-62·8–-1·57) |
| Spain | 57·0 (49·7–64·1) | 0·0791 (0·0695–0·0886) | 84·0 (68·1–100) | 0·0695 (0·0576–0·0828) | 47·3 (25·6–72·6) | -12·1 (-24·9–4·14) |
| Sweden | 16·1 (14·0–18·0) | 0·0832 (0·0730–0·0924) | 34·2 (28·0–40·3) | 0·129 (0·105–0·151) | 112 (75·9–152) | 54·5 (28·5–82·4) |
| Switzerland | 9·22 (7·98–10·3) | 0·0685 (0·0602–0·0756) | 12·1 (9·72–14·7) | 0·0549 (0·0443–0·0680) | 31·1 (12·3–59·1) | -19·8 (-32·1–-1·62) |
| United Kingdom | 162 (149–170) | 0·157 (0·146–0·163) | 279 (245–310) | 0·194 (0·173–0·216) | 71·7 (61·3–85·0) | 23·5 (16·1–33·9) |
| **Latin America and Caribbean** | **573 (543–599)** | **0·184 (0·174–0·193)** | **1390 (1290–1520)** | **0·226 (0·209–0·246)** | **143 (129–160)** | **23·2 (16·2–31·0)** |
| Andean Latin America | 18·3 (12·4–22·5) | 0·0627 (0·0422–0·0767) | 42·5 (24·9–59·1) | 0·0721 (0·0425–0·0999) | 131 (71·1–209) | 15·1 (-14·9–52·3) |
| Bolivia (Plurinational State of) | 5·51 (2·58–8·30) | 0·120 (0·0556–0·178) | 10·9 (4·26–17·5) | 0·126 (0·0488–0·200) | 98·7 (26·7–222) | 4·74 (-31·6–67·4) |
| Ecuador | 6·26 (5·56–7·03) | 0·0809 (0·0711–0·0909) | 15·5 (10·3–26·8) | 0·0986 (0·0674–0·165) | 147 (63·2–323) | 21·9 (-17·3–103) |
| Peru | 6·57 (3·83–8·83) | 0·0390 (0·0226–0·0527) | 16·0 (6·93–24·1) | 0·0469 (0·0204–0·0705) | 144 (45·2–263) | 20·3 (-28·3–80·6) |
| Caribbean | 58·6 (52·8–64·9) | 0·175 (0·158–0·194) | 114 (95·8–136) | 0·212 (0·177–0·252) | 95·3 (66·6–126) | 20·8 (3·26–39·8) |
| Antigua and Barbuda | 0·0539 (0·0475–0·0609) | 0·0872 (0·0772–0·0990) | 0·107 (0·0921–0·125) | 0·103 (0·0889–0·120) | 98·3 (64·1–141) | 18·0 (-2·69–43·4) |
| Bahamas | 0·298 (0·266–0·332) | 0·134 (0·119–0·149) | 0·643 (0·500–0·818) | 0·159 (0·124–0·201) | 115 (63·7–181) | 18·4 (-8·78–53·4) |
| Barbados | 0·749 (0·662–0·845) | 0·230 (0·206–0·257) | 1·43 (1·05–1·82) | 0·280 (0·205–0·355) | 90·5 (36·6–154) | 21·7 (-12·2–60·6) |
| Belize | 0·264 (0·240–0·294) | 0·209 (0·189–0·235) | 0·663 (0·552–0·782) | 0·216 (0·180–0·256) | 151 (104–201) | 3·31 (-15·4–24·1) |
| Bermuda | 0·0696 (0·0617–0·0777) | 0·0921 (0·0819–0·103) | 0·121 (0·0959–0·148) | 0·0878 (0·0701–0·107) | 73·7 (36·7–120) | -4·69 (-24·8–19·5) |
| Cuba | 25·1 (22·9–27·5) | 0·197 (0·179–0·215) | 47·9 (39·3–56·6) | 0·243 (0·200–0·286) | 90·6 (54·2–130) | 23·6 (-0·0912–48·8) |
| Dominica | 0·0199 (0·0102–0·0275) | 0·0322 (0·0165–0·0442) | 0·0376 (0·0156–0·0574) | 0·0461 (0·0192–0·0699) | 88·9 (28·9–189) | 43·0 (-2·72–116) |
| Dominican Republic | 2·56 (1·59–3·59) | 0·0461 (0·0281–0·0660) | 5·15 (3·26–8·00) | 0·0514 (0·0325–0·0803) | 101 (28·4–211) | 11·4 (-29·4–74·3) |
| Grenada | 0·171 (0·144–0·214) | 0·225 (0·187–0·283) | 0·342 (0·277–0·431) | 0·301 (0·245–0·378) | 100 (64·2–142) | 34·1 (8·75–62·1) |
| Guyana | 1·49 (1·28–1·71) | 0·339 (0·291–0·392) | 2·48 (1·86–3·29) | 0·384 (0·289–0·503) | 66·6 (21·2–128) | 13·1 (-18·6–53·9) |
| Haiti | 7·13 (3·31–11·7) | 0·168 (0·0790–0·284) | 13·5 (5·37–23·5) | 0·183 (0·0700–0·327) | 89·7 (17·6–193) | 9·23 (-29·9–71·9) |
| Jamaica | 4·92 (4·35–5·51) | 0·223 (0·198–0·250) | 9·75 (7·25–12·7) | 0·309 (0·229–0·404) | 98·2 (43·9–161) | 38·3 (0·479–81·7) |
| Puerto Rico | 3·74 (3·37–4·13) | 0·0804 (0·0727–0·0885) | 8·60 (6·98–10·5) | 0·125 (0·101–0·152) | 130 (83·3–186) | 55·3 (22·9–92·9) |
| Saint Kitts and Nevis | 0·0193 (0·0169–0·0218) | 0·0500 (0·0437–0·0561) | 0·0464 (0·0343–0·0641) | 0·0691 (0·0528–0·0920) | 140 (73·8–241) | 38·4 (2·38–89·7) |
| Saint Lucia | 0·181 (0·162–0·202) | 0·170 (0·154–0·188) | 0·460 (0·365–0·567) | 0·197 (0·157–0·243) | 154 (98·9–218) | 15·9 (-9·10–44·2) |
| Saint Vincent and the Grenadines | 0·113 (0·100–0·127) | 0·139 (0·123–0·157) | 0·259 (0·213–0·311) | 0·187 (0·156–0·224) | 129 (83·2–182) | 34·8 (7·83–65·3) |
| Suriname | 0·740 (0·380–1·08) | 0·229 (0·116–0·334) | 1·47 (0·616–2·34) | 0·233 (0·0974–0·367) | 98·9 (26·4–202) | 1·46 (-35·4–54·3) |
| Trinidad and Tobago | 8·54 (7·76–9·35) | 0·796 (0·724–0·875) | 17·2 (12·8–22·8) | 0·908 (0·684–1·20) | 101 (46·5–170) | 14·1 (-16·4–53·1) |
| United States Virgin Islands | 0·385 (0·273–0·543) | 0·347 (0·245–0·486) | 0·344 (0·200–0·576) | 0·204 (0·124–0·331) | -10·8 (-44·4–43·5) | -41·1 (-63·1–-6·93) |
| Central Latin America | 293 (277–309) | 0·245 (0·230–0·258) | 541 (479–614) | 0·218 (0·193–0·245) | 84·5 (65·8–105) | -11·1 (-20·0–-1·72) |
| Colombia | 21·4 (19·6–24·0) | 0·0790 (0·0720–0·0880) | 39·7 (32·7–48·6) | 0·0717 (0·0590–0·0874) | 85·4 (52·0–125) | -9·27 (-25·5–10·3) |
| Costa Rica | 0·838 (0·737–0·952) | 0·0317 (0·0277–0·0363) | 1·54 (1·28–1·82) | 0·0280 (0·0231–0·0331) | 84·2 (50·9–123) | -11·7 (-27·9–7·62) |
| El Salvador | 2·32 (1·34–3·11) | 0·0583 (0·0337–0·0786) | 3·59 (2·00–5·37) | 0·0566 (0·0317–0·0853) | 54·4 (12·5–115) | -2·93 (-30·8–36·6) |
| Guatemala | 9·67 (8·75–10·7) | 0·206 (0·185–0·228) | 17·9 (14·6–21·6) | 0·163 (0·132–0·196) | 85·3 (47·8–126) | -20·8 (-36·1–-3·71) |
| Honduras | 13·6 (5·80–21·7) | 0·458 (0·189–0·746) | 30·2 (13·6–47·3) | 0·497 (0·221–0·784) | 122 (36·3–248) | 8·54 (-34·7–72·4) |
| Mexico | 202 (191–214) | 0·347 (0·325–0·369) | 367 (314–435) | 0·295 (0·254–0·346) | 81·2 (57·7–111) | -15·0 (-25·7–-2·56) |
| Nicaragua | 1·84 (1·11–2·37) | 0·0745 (0·0440–0·0971) | 3·72 (2·12–5·68) | 0·0759 (0·0431–0·115) | 102 (45·8–210) | 1·88 (-26·2–55·8) |
| Panama | 1·23 (1·10–1·35) | 0·0572 (0·0513–0·0631) | 2·48 (1·87–3·10) | 0·0554 (0·0417–0·0693) | 102 (55·6–155) | -3·18 (-25·4–23·0) |
| Venezuela (Bolivarian Republic of) | 40·1 (36·2–43·2) | 0·276 (0·248–0·299) | 75·4 (55·7–99·2) | 0·260 (0·194–0·341) | 88·3 (37·7–150) | -5·87 (-31·3–23·9) |
| Tropical Latin America | 203 (189–215) | 0·157 (0·144–0·167) | 697 (638–743) | 0·275 (0·251–0·294) | 244 (222–269) | 75·5 (64·7–89·7) |
| Brazil | 201 (187–213) | 0·159 (0·146–0·170) | 687 (630–733) | 0·278 (0·254–0·296) | 242 (222–268) | 74·6 (63·9–88·6) |
| Paraguay | 2·12 (1·51–3·38) | 0·0683 (0·0491–0·109) | 9·60 (3·56–15·0) | 0·170 (0·0633–0·271) | 352 (80·7–626) | 148 (0·288–299) |
| **North Africa and Middle East** | **179 (97·4–235)** | **0·0820 (0·0441–0·108)** | **394 (182–510)** | **0·0995 (0·0455–0·128)** | **120 (49·7–187)** | **21·4 (-17·8–61·4)** |
| North Africa and Middle East | 179 (97·4–235) | 0·0820 (0·0441–0·108) | 394 (182–510) | 0·0995 (0·0455–0·128) | 120 (49·7–187) | 21·4 (-17·8–61·4) |
| Afghanistan | 5·63 (2·31–9·39) | 0·0719 (0·0312–0·121) | 18·9 (4·07–37·5) | 0·161 (0·0318–0·334) | 237 (10·7–525) | 124 (-30·1–311) |
| Algeria | 4·76 (2·41–7·76) | 0·0317 (0·0165–0·0528) | 22·0 (4·37–39·7) | 0·0784 (0·0158–0·140) | 364 (46·5–713) | 148 (-22·4–366) |
| Bahrain | 0·111 (0·0671–0·254) | 0·0643 (0·0387–0·153) | 0·735 (0·205–1·47) | 0·158 (0·0414–0·387) | 559 (46·4–1760) | 145 (-47·9–662) |
| Egypt | 17·7 (11·3–32·6) | 0·0633 (0·0400–0·127) | 71·3 (37·9–98·3) | 0·154 (0·0832–0·217) | 303 (72·1–626) | 144 (2·08–343) |
| Iran (Islamic Republic of) | 88·0 (34·6–123) | 0·252 (0·0937–0·355) | 115 (59·3–145) | 0·163 (0·0794–0·206) | 30·4 (-1·54–120) | -35·2 (-50·8–6·51) |
| Iraq | 8·50 (4·18–13·1) | 0·0754 (0·0349–0·118) | 16·9 (7·56–24·4) | 0·0830 (0·0360–0·123) | 98·9 (20·0–238) | 10·1 (-35·7–91·7) |
| Jordan | 0·155 (0·0846–0·364) | 0·00845 (0·00445–0·0204) | 0·962 (0·336–1·51) | 0·0161 (0·00542–0·0255) | 521 (53·5–1390) | 91·1 (-51·2–344) |
| Kuwait | 0·00376 (0·00335–0·00416) | 0·000453 (0·000396–0·000513) | 0·878 (0·638–1·20) | 0·0342 (0·0246–0·0478) | 23300 (16400–31800) | 7440 (4970–10400) |
| Lebanon | 2·53 (1·38–4·01) | 0·0943 (0·0505–0·152) | 5·17 (2·85–7·62) | 0·0799 (0·0455–0·118) | 105 (19·7–241) | -15·2 (-49·1–39·3) |
| Libya | 0·967 (0·553–1·39) | 0·0364 (0·0200–0·0546) | 6·07 (1·31–10·1) | 0·121 (0·0260–0·202) | 528 (103–1050) | 231 (3·73–526) |
| Morocco | 5·66 (3·06–9·15) | 0·0304 (0·0160–0·0514) | 29·6 (5·76–52·0) | 0·0930 (0·0181–0·161) | 424 (55·5–802) | 206 (-8·54–440) |
| Oman | 0·150 (0·0907–0·233) | 0·0180 (0·0105–0·0276) | 0·593 (0·172–0·975) | 0·0373 (0·0109–0·0642) | 294 (25·9–733) | 107 (-29·5–330) |
| Palestine | 0·433 (0·244–0·655) | 0·0443 (0·0246–0·0659) | 0·649 (0·366–1·22) | 0·0318 (0·0176–0·0584) | 49·8 (-12·1–175) | -28·1 (-59·2–30·5) |
| Qatar | 0·193 (0·0519–0·317) | 0·146 (0·0454–0·244) | 0·325 (0·105–0·738) | 0·0639 (0·0220–0·149) | 68·1 (-24·0–812) | -56·3 (-81·1–108) |
| Saudi Arabia | 0·149 (0·0675–0·435) | 0·00172 (0·000799–0·00471) | 1·57 (0·500–2·63) | 0·00805 (0·00222–0·0139) | 953 (121–2990) | 368 (-8·32–1250) |
| Sudan | 5·65 (2·74–9·29) | 0·0378 (0·0201–0·0623) | 18·3 (3·93–33·1) | 0·0921 (0·0195–0·170) | 223 (-1·24–514) | 144 (-29·0–332) |
| Syrian Arab Republic | 6·08 (3·66–9·65) | 0·0946 (0·0546–0·146) | 8·77 (4·79–14·4) | 0·0832 (0·0466–0·135) | 44·2 (-21·0–140) | -12·0 (-50·1–49·3) |
| Tunisia | 1·65 (0·860–2·50) | 0·0253 (0·0132–0·0388) | 6·65 (1·23–12·1) | 0·0543 (0·0100–0·0997) | 304 (13·7–678) | 115 (-38·9–303) |
| Türkiye | 28·3 (17·4–44·9) | 0·0616 (0·0373–0·101) | 57·7 (35·3–86·9) | 0·0678 (0·0412–0·102) | 104 (28·1–245) | 9·94 (-32·8–87·4) |
| United Arab Emirates | 0·420 (0·185–0·791) | 0·0869 (0·0370–0·185) | 1·35 (0·760–2·31) | 0·101 (0·0520–0·181) | 222 (34·6–561) | 16·5 (-61·6–168) |
| Yemen | 2·23 (1·04–3·85) | 0·0295 (0·0141–0·0537) | 10·5 (1·62–23·8) | 0·0775 (0·0116–0·181) | 371 (24·1–717) | 163 (-31·1–371) |
| **South Asia** | **2160 (1210–3170)** | **0·300 (0·175–0·447)** | **3910 (2180–5950)** | **0·281 (0·159–0·428)** | **80·9 (41·9–138)** | **-6·29 (-26·8–21·9)** |
| South Asia | 2160 (1210–3170) | 0·300 (0·175–0·447) | 3910 (2180–5950) | 0·281 (0·159–0·428) | 80·9 (41·9–138) | -6·29 (-26·8–21·9) |
| Bangladesh | 129 (67·5–234) | 0·252 (0·129–0·461) | 218 (111–403) | 0·172 (0·0866–0·314) | 68·6 (4·15–161) | -31·5 (-58·1–6·11) |
| Bhutan | 0·865 (0·391–1·74) | 0·276 (0·122–0·557) | 1·53 (0·681–3·03) | 0·265 (0·118–0·518) | 76·9 (14·8–172) | -4·12 (-37·1–45·2) |
| India | 1740 (950–2540) | 0·292 (0·166–0·437) | 3220 (1750–4930) | 0·286 (0·156–0·438) | 85·5 (42·0–147) | -2·17 (-25·3–29·1) |
| Nepal | 19·7 (10·8–33·1) | 0·176 (0·0932–0·299) | 47·6 (24·6–82·1) | 0·224 (0·115–0·388) | 141 (52·2–266) | 27·0 (-20·1–95·8) |
| Pakistan | 276 (147–452) | 0·415 (0·228–0·694) | 425 (231–693) | 0·368 (0·203–0·589) | 53·8 (4·57–120) | -11·5 (-39·3–25·5) |
| **Southeast Asia, East Asia, and Oceania** | **4320 (2110–5260)** | **0·344 (0·169–0·422)** | **5150 (2890–6490)** | **0·201 (0·115–0·254)** | **19·3 (-1·66–64·9)** | **-41·4 (-51·8–-19·2)** |
| East Asia | 3110 (1570–3900) | 0·341 (0·170–0·433) | 3000 (1960–4180) | 0·154 (0·101–0·213) | -3·60 (-28·4–57·0) | -54·9 (-66·4–-26·4) |
| China | 3040 (1520–3820) | 0·348 (0·172–0·443) | 2860 (1840–4040) | 0·152 (0·0979–0·215) | -6·03 (-31·1–54·1) | -56·3 (-67·8–-28·3) |
| Democratic People's Republic of Korea | 56·4 (22·7–88·3) | 0·300 (0·121–0·461) | 74·0 (35·2–113) | 0·232 (0·110–0·360) | 31·1 (-11·0–94·3) | -22·8 (-47·2–13·6) |
| Taiwan (Province of China) | 15·2 (13·3–17·2) | 0·0766 (0·0671–0·0865) | 69·0 (56·8–80·8) | 0·160 (0·133–0·187) | 353 (265–462) | 108 (68·5–160) |
| Oceania | 0·405 (0·120–0·763) | 0·0106 (0·00334–0·0196) | 0·558 (0·211–0·966) | 0·00791 (0·00294–0·0138) | 37·7 (-0·876–103) | -25·3 (-46·7–8·41) |
| American Samoa | 0·00678 (0·00359–0·00960) | 0·0223 (0·0118–0·0317) | 0·00966 (0·00448–0·0163) | 0·0225 (0·0101–0·0378) | 42·6 (-12·4–135) | 0·703 (-39·8–71·8) |
| Cook Islands | 0·00155 (0·000402–0·00257) | 0·00988 (0·00265–0·0161) | 0·00131 (0·000501–0·00221) | 0·00513 (0·00200–0·00870) | -15·8 (-49·2–67·5) | -48·1 (-68·8–-0·793) |
| Fiji | 0·0143 (0·00809–0·0358) | 0·00344 (0·00187–0·00868) | 0·0805 (0·0253–0·135) | 0·0113 (0·00345–0·0196) | 462 (27·1–1340) | 227 (-26·1–784) |
| Guam | 0·0118 (0·00311–0·0179) | 0·0100 (0·00267–0·0153) | 0·00393 (0·00191–0·0116) | 0·00197 (0·000951–0·00582) | -66·6 (-88·2–74·7) | -80·4 (-93·2–0·205) |
| Kiribati | 0·0175 (0·00468–0·0319) | 0·0388 (0·0108–0·0706) | 0·0191 (0·00681–0·0330) | 0·0265 (0·00938–0·0466) | 8·98 (-33·2–91·6) | -31·9 (-58·9–15·2) |
| Marshall Islands | 0·00532 (0·00143–0·00993) | 0·0239 (0·00660–0·0431) | 0·00655 (0·00217–0·0120) | 0·0180 (0·00612–0·0310) | 23·0 (-26·5–110) | -24·7 (-55·0–28·1) |
| Micronesia (Federated States of) | 0·0170 (0·00453–0·0309) | 0·0303 (0·00840–0·0550) | 0·0141 (0·00527–0·0241) | 0·0192 (0·00719–0·0325) | -17·1 (-50·4–50·6) | -36·8 (-62·1–12·2) |
| Nauru | 0·00234 (0·000588–0·00453) | 0·0375 (0·00954–0·0666) | 0·00161 (0·000546–0·00282) | 0·0243 (0·00834–0·0411) | -31·4 (-61·1–23·9) | -35·2 (-61·2–12·8) |
| Niue | 0·000367 (0·000104–0·000647) | 0·0171 (0·00486–0·0299) | 0·000270 (0·000102–0·000432) | 0·0128 (0·00488–0·0202) | -26·3 (-54·5–27·4) | -24·8 (-53·4–29·6) |
| Northern Mariana Islands | 0·00307 (0·00113–0·00486) | 0·0102 (0·00356–0·0159) | 0·000698 (0·000225–0·00238) | 0·00154 (0·000503–0·00526) | -77·3 (-94·3–8·69) | -84·9 (-96·1–-27·0) |
| Palau | 0·00330 (0·000928–0·00559) | 0·0235 (0·00681–0·0388) | 0·00347 (0·00133–0·00563) | 0·0164 (0·00625–0·0263) | 5·17 (-34·5–75·9) | -30·1 (-56·6–15·9) |
| Papua New Guinea | 0·217 (0·0460–0·478) | 0·00908 (0·00191–0·0195) | 0·293 (0·0870–0·603) | 0·00624 (0·00179–0·0129) | 34·6 (-16·8–133) | -31·2 (-59·4–23·9) |
| Samoa | 0·0182 (0·00511–0·0321) | 0·0180 (0·00532–0·0320) | 0·0194 (0·00710–0·0327) | 0·0137 (0·00500–0·0227) | 6·56 (-34·3–81·0) | -23·9 (-52·0–27·1) |
| Solomon Islands | 0·0364 (0·00914–0·0760) | 0·0190 (0·00512–0·0378) | 0·0488 (0·0185–0·0872) | 0·0133 (0·00512–0·0235) | 34·0 (-18·3–146) | -30·1 (-57·9–18·5) |
| Tokelau | 0·000212 (0·0000573–0·000385) | 0·0160 (0·00434–0·0295) | 0·000178 (0·0000667–0·000306) | 0·0122 (0·00459–0·0208) | -16·0 (-49·5–56·9) | -23·7 (-53·5–43·7) |
| Tonga | 0·0126 (0·00410–0·0217) | 0·0205 (0·00663–0·0358) | 0·0116 (0·00458–0·0207) | 0·0147 (0·00576–0·0259) | -7·26 (-46·2–49·5) | -28·2 (-59·2–16·2) |
| Tuvalu | 0·00167 (0·000460–0·00307) | 0·0212 (0·00595–0·0380) | 0·00136 (0·000495–0·00238) | 0·0133 (0·00495–0·0232) | -18·4 (-48·7–37·0) | -37·1 (-59·5–3·69) |
| Vanuatu | 0·0118 (0·00344–0·0218) | 0·0136 (0·00394–0·0245) | 0·0178 (0·00682–0·0317) | 0·0105 (0·00421–0·0183) | 51·3 (-2·70–171) | -22·6 (-48·6–37·9) |
| Southeast Asia | 1200 (513–1570) | 0·371 (0·167–0·484) | 2150 (879–2760) | 0·366 (0·157–0·470) | 78·4 (46·0–119) | -1·22 (-18·4–20·7) |
| Cambodia | 19·5 (3·18–33·8) | 0·344 (0·0597–0·592) | 37·6 (5·70–76·4) | 0·339 (0·0555–0·672) | 92·6 (26·0–185) | -1·46 (-33·9–47·9) |
| Indonesia | 354 (41·3–586) | 0·283 (0·0365–0·469) | 679 (83·5–1100) | 0·310 (0·0446–0·495) | 91·8 (41·4–165) | 9·36 (-19·1–49·9) |
| Lao People's Democratic Republic | 8·33 (1·18–15·8) | 0·330 (0·0490–0·629) | 10·6 (1·62–20·2) | 0·253 (0·0412–0·484) | 27·2 (-15·9–102) | -23·6 (-50·4–17·1) |
| Malaysia | 9·44 (5·37–14·7) | 0·0814 (0·0457–0·128) | 25·5 (15·0–53·4) | 0·100 (0·0587–0·213) | 170 (68·9–549) | 23·1 (-23·2–193) |
| Maldives | 0·0174 (0·00834–0·0273) | 0·0171 (0·00807–0·0266) | 0·0309 (0·0143–0·0508) | 0·0105 (0·00486–0·0168) | 77·8 (-6·61–242) | -38·9 (-67·6–19·1) |
| Mauritius | 0·123 (0·111–0·135) | 0·0140 (0·0126–0·0154) | 0·467 (0·400–0·536) | 0·0268 (0·0230–0·0307) | 279 (218–336) | 91·7 (62·9–121) |
| Myanmar | 95·0 (14·7–167) | 0·336 (0·0536–0·588) | 119 (20·0–220) | 0·265 (0·0476–0·487) | 25·0 (-17·1–104) | -21·0 (-47·6–25·6) |
| Philippines | 241 (158–321) | 0·601 (0·404–0·833) | 541 (333–682) | 0·695 (0·456–0·889) | 124 (80·8–178) | 15·6 (-7·49–44·3) |
| Seychelles | 0·108 (0·0187–0·179) | 0·162 (0·0282–0·266) | 0·187 (0·0277–0·320) | 0·175 (0·0266–0·299) | 72·8 (19·8–164) | 8·04 (-24·8–68·3) |
| Sri Lanka | 4·80 (3·14–8·33) | 0·0366 (0·0235–0·0637) | 11·8 (5·40–19·1) | 0·0479 (0·0222–0·0771) | 146 (16·0–341) | 30·9 (-36·8–129) |
| Thailand | 422 (276–611) | 0·887 (0·573–1·25) | 624 (397–894) | 0·588 (0·373–0·842) | 47·9 (1·86–118) | -33·8 (-54·5–-3·70) |
| Timor-Leste | 0·699 (0·105–1·57) | 0·199 (0·0319–0·427) | 1·68 (0·288–3·66) | 0·216 (0·0401–0·479) | 140 (67·1–269) | 8·38 (-27·1–57·0) |
| Viet Nam | 47·2 (4·26–86·1) | 0·0968 (0·00905–0·178) | 94·7 (8·19–172) | 0·106 (0·00976–0·192) | 101 (37·6–195) | 9·82 (-25·7–60·0) |
| **Sub-Saharan Africa** | **579 (281–1300)** | **0·213 (0·0984–0·501)** | **842 (406–1870)** | **0·179 (0·0831–0·413)** | **45·4 (11·9–91·2)** | **-15·8 (-35·2–10·5)** |
| Central Sub-Saharan Africa | 25·4 (11·5–60·4) | 0·0954 (0·0390–0·233) | 45·1 (20·0–99·4) | 0·0899 (0·0385–0·204) | 77·8 (19·4–174) | -5·75 (-37·5–40·8) |
| Angola | 4·99 (2·28–11·1) | 0·101 (0·0399–0·244) | 9·42 (4·33–19·6) | 0·0890 (0·0387–0·195) | 88·8 (19·9–225) | -11·6 (-47·5–51·6) |
| Central African Republic | 1·69 (0·850–4·21) | 0·127 (0·0562–0·325) | 2·43 (1·13–5·71) | 0·113 (0·0465–0·275) | 44·4 (-11·1–144) | -11·0 (-44·0–45·1) |
| Congo | 1·95 (1·04–4·65) | 0·152 (0·0766–0·377) | 3·12 (1·54–7·15) | 0·126 (0·0613–0·303) | 60·2 (1·99–164) | -17·1 (-46·8–32·5) |
| Democratic Republic of the Congo | 15·7 (6·35–38·1) | 0·0866 (0·0311–0·219) | 28·4 (11·2–64·5) | 0·0847 (0·0325–0·201) | 80·6 (11·2–191) | -2·30 (-41·4–57·3) |
| Equatorial Guinea | 0·194 (0·0849–0·442) | 0·0866 (0·0350–0·201) | 0·524 (0·239–1·18) | 0·112 (0·0498–0·256) | 169 (58·7–374) | 28·8 (-24·9–123) |
| Gabon | 0·842 (0·379–1·96) | 0·137 (0·0607–0·330) | 1·22 (0·580–2·84) | 0·130 (0·0607–0·310) | 45·6 (-9·27–137) | -5·36 (-41·2–54·1) |
| Eastern Sub-Saharan Africa | 288 (113–761) | 0·321 (0·116–0·873) | 414 (156–1010) | 0·254 (0·0918–0·621) | 43·7 (11·3–101) | -20·9 (-39·2–7·44) |
| Burundi | 5·79 (1·97–16·0) | 0·234 (0·0781–0·649) | 8·97 (2·77–23·7) | 0·194 (0·0570–0·525) | 55·1 (-10·7–159) | -17·3 (-52·5–38·0) |
| Comoros | 0·563 (0·191–1·51) | 0·227 (0·0734–0·626) | 0·985 (0·341–2·45) | 0·213 (0·0708–0·546) | 74·9 (6·47–195) | -6·40 (-42·4–54·2) |
| Djibouti | 0·761 (0·248–1·88) | 0·348 (0·108–0·853) | 2·03 (0·633–5·07) | 0·348 (0·111–0·853) | 166 (63·5–335) | -0·0656 (-41·1–65·7) |
| Eritrea | 3·99 (1·63–10·4) | 0·272 (0·117–0·720) | 6·85 (2·78–16·7) | 0·240 (0·0959–0·595) | 71·8 (8·77–191) | -12·0 (-42·9–45·7) |
| Ethiopia | 121 (37·1–315) | 0·510 (0·146–1·37) | 128 (34·5–338) | 0·310 (0·0804–0·817) | 6·09 (-31·8–66·9) | -39·2 (-61·5–-6·76) |
| Kenya | 22·1 (10·1–57·8) | 0·202 (0·0872–0·540) | 52·6 (24·4–130) | 0·237 (0·109–0·593) | 139 (56·0–244) | 17·1 (-23·5–69·5) |
| Madagascar | 10·2 (3·86–27·1) | 0·180 (0·0626–0·468) | 18·1 (7·02–45·2) | 0·170 (0·0628–0·445) | 77·7 (13·0–184) | -5·75 (-40·7–51·4) |
| Malawi | 11·2 (4·75–28·0) | 0·258 (0·101–0·649) | 15·7 (7·02–37·1) | 0·209 (0·0893–0·510) | 40·3 (-8·06–122) | -19·2 (-46·1–26·5) |
| Mozambique | 19·7 (6·57–54·3) | 0·292 (0·0907–0·839) | 37·0 (14·5–89·9) | 0·330 (0·125–0·811) | 88·2 (24·3–214) | 12·8 (-27·4–89·9) |
| Rwanda | 7·08 (2·17–19·1) | 0·246 (0·0792–0·693) | 9·04 (2·77–24·3) | 0·150 (0·0451–0·406) | 27·8 (-33·2–152) | -39·0 (-66·8–21·4) |
| Somalia | 13·1 (4·39–36·2) | 0·409 (0·124–1·16) | 23·1 (6·46–62·8) | 0·362 (0·0914–1·01) | 75·7 (-2·42–193) | -11·5 (-46·0–40·5) |
| South Sudan | 9·24 (2·74–27·3) | 0·351 (0·0942–1·05) | 13·1 (4·32–38·1) | 0·359 (0·110–1·04) | 42·1 (-12·9–144) | 2·26 (-38·2–75·0) |
| Uganda | 16·4 (5·38–45·2) | 0·209 (0·0665–0·586) | 25·4 (9·50–61·5) | 0·170 (0·0613–0·430) | 55·0 (-2·14–172) | -18·4 (-48·9–45·1) |
| United Republic of Tanzania | 33·1 (11·7–86·7) | 0·243 (0·0810–0·650) | 50·1 (17·4–123) | 0·200 (0·0684–0·496) | 51·2 (-7·12–159) | -17·5 (-50·4–42·4) |
| Zambia | 13·9 (5·73–34·7) | 0·424 (0·164–1·10) | 22·4 (7·16–57·8) | 0·322 (0·109–0·810) | 60·8 (-28·9–319) | -24·1 (-63·7–79·7) |
| Southern Sub-Saharan Africa | 24·4 (14·7–31·2) | 0·0664 (0·0397–0·0841) | 33·6 (22·2–47·7) | 0·0590 (0·0391–0·0870) | 37·7 (10·3–84·4) | -11·1 (-28·8–21·8) |
| Botswana | 0·731 (0·246–1·37) | 0·0945 (0·0326–0·173) | 0·929 (0·374–1·79) | 0·0661 (0·0281–0·119) | 27·1 (-28·5–162) | -30·1 (-59·8–33·0) |
| Eswatini | 0·659 (0·281–1·11) | 0·164 (0·0721–0·281) | 0·764 (0·329–1·23) | 0·129 (0·0546–0·205) | 15·9 (-26·0–79·1) | -21·6 (-49·2–20·4) |
| Lesotho | 0·867 (0·401–1·36) | 0·0853 (0·0406–0·134) | 1·46 (0·666–2·37) | 0·130 (0·0603–0·208) | 68·0 (1·42–162) | 52·4 (-5·30–141) |
| Namibia | 0·775 (0·298–1·28) | 0·0930 (0·0353–0·155) | 0·948 (0·391–1·56) | 0·0711 (0·0307–0·114) | 22·3 (-23·0–90·6) | -23·6 (-50·8–15·9) |
| South Africa | 17·8 (11·0–24·0) | 0·0620 (0·0380–0·0820) | 24·0 (17·0–37·6) | 0·0534 (0·0374–0·0859) | 35·0 (8·46–93·8) | -13·8 (-31·2–24·5) |
| Zimbabwe | 3·56 (2·03–5·52) | 0·0725 (0·0419–0·109) | 5·46 (2·77–8·51) | 0·0751 (0·0384–0·116) | 53·1 (-4·71–130) | 3·67 (-34·8–53·0) |
| Western Sub-Saharan Africa | 241 (119–513) | 0·205 (0·0950–0·436) | 349 (170–773) | 0·176 (0·0817–0·400) | 44·8 (1·54–96·8) | -14·0 (-41·4–16·8) |
| Benin | 6·32 (2·66–15·2) | 0·221 (0·0849–0·497) | 9·36 (3·69–21·5) | 0·173 (0·0612–0·411) | 48·2 (-9·52–137) | -21·8 (-53·0–16·6) |
| Burkina Faso | 12·5 (4·85–29·2) | 0·220 (0·0766–0·510) | 17·8 (6·49–42·3) | 0·185 (0·0590–0·462) | 42·1 (-25·3–119) | -15·6 (-49·6–26·2) |
| Cabo Verde | 0·192 (0·0681–0·501) | 0·0676 (0·0236–0·176) | 0·439 (0·153–1·29) | 0·100 (0·0347–0·296) | 129 (16·7–358) | 47·9 (-24·9–200) |
| Cameroon | 18·0 (7·84–41·0) | 0·281 (0·123–0·621) | 26·5 (10·8–65·2) | 0·208 (0·0817–0·523) | 47·4 (-13·7–132) | -26·1 (-58·1–9·63) |
| Chad | 7·73 (3·00–18·2) | 0·207 (0·0724–0·494) | 11·9 (4·61–27·9) | 0·186 (0·0616–0·444) | 53·8 (-8·26–137) | -10·3 (-48·2–36·8) |
| Côte d'Ivoire | 14·3 (6·76–32·0) | 0·240 (0·112–0·534) | 19·7 (8·43–46·7) | 0·179 (0·0715–0·443) | 38·0 (-24·9–128) | -25·3 (-59·8–22·6) |
| Gambia | 1·31 (0·540–2·88) | 0·233 (0·0845–0·524) | 2·21 (0·844–5·36) | 0·227 (0·0828–0·555) | 68·6 (-9·39–159) | -2·39 (-46·5–48·1) |
| Ghana | 24·9 (11·6–50·0) | 0·296 (0·140–0·581) | 46·8 (20·4–93·9) | 0·299 (0·131–0·597) | 88·4 (3·87–247) | 0·914 (-49·0–99·4) |
| Guinea | 7·74 (3·15–18·7) | 0·164 (0·0632–0·401) | 9·08 (3·50–21·9) | 0·154 (0·0529–0·389) | 17·2 (-30·6–83·1) | -6·33 (-44·4–45·9) |
| Guinea-Bissau | 1·74 (0·884–4·07) | 0·342 (0·166–0·759) | 2·00 (0·894–4·76) | 0·267 (0·114–0·652) | 14·8 (-42·9–76·5) | -21·8 (-54·1–9·87) |
| Liberia | 2·29 (0·967–4·89) | 0·176 (0·0703–0·390) | 3·67 (1·37–9·17) | 0·173 (0·0593–0·455) | 60·4 (-11·5–150) | -1·46 (-44·8–50·8) |
| Mali | 10·8 (4·18–25·0) | 0·214 (0·0750–0·502) | 16·3 (6·13–38·9) | 0·180 (0·0573–0·440) | 51·2 (-20·4–137) | -15·6 (-52·8–24·1) |
| Mauritania | 2·69 (1·15–6·56) | 0·216 (0·0879–0·527) | 3·95 (1·36–10·4) | 0·190 (0·0637–0·505) | 47·1 (-18·7–116) | -11·9 (-50·8–27·1) |
| Niger | 8·64 (3·29–22·0) | 0·188 (0·0556–0·441) | 12·0 (4·00–27·5) | 0·138 (0·0375–0·340) | 38·6 (-41·7–142) | -26·5 (-61·0–15·6) |
| Nigeria | 105 (46·2–217) | 0·183 (0·0761–0·390) | 140 (64·1–313) | 0·151 (0·0644–0·350) | 33·0 (-17·3–102) | -17·9 (-52·2–25·9) |
| Sao Tome and Principe | 0·129 (0·0559–0·299) | 0·179 (0·0762–0·422) | 0·188 (0·0746–0·449) | 0·172 (0·0660–0·426) | 45·9 (-8·40–114) | -3·53 (-40·0–37·7) |
| Senegal | 8·73 (3·80–19·3) | 0·193 (0·0757–0·431) | 14·3 (4·89–37·3) | 0·190 (0·0619–0·502) | 63·7 (-31·5–157) | -1·61 (-44·9–48·7) |
| Sierra Leone | 4·10 (1·73–9·32) | 0·172 (0·0676–0·382) | 5·77 (2·32–12·6) | 0·150 (0·0562–0·327) | 40·5 (-25·6–118) | -12·9 (-50·8–29·9) |
| Togo | 4·11 (1·94–9·30) | 0·225 (0·0970–0·532) | 7·50 (3·18–16·8) | 0·199 (0·0785–0·458) | 82·6 (0·212–188) | -11·6 (-48·7–34·2) |
|  |  |  |  |  |  |  |

# Reference

1. GBD 2021 Demographics Collaborators. Global age-sex-specific mortality, life expectancy, and population estimates in 204 countries and territories and 811 subnational locations, 1950-2021, and the impact of the COVID-19 pandemic: a comprehensive demographic analysis for the Global Burden of Disease Study 2021. Lancet. 2024 May 18;403(10440):1989-2056. doi: 10.1016/S0140-6736(24)00476-8. Epub 2024 Mar 11. PMID: 38484753; PMCID: PMC11126395.

2. GBD 2021 Causes of Death Collaborators. Global burden of 288 causes of death and life expectancy decomposition in 204 countries and territories and 811 subnational locations, 1990-2021: a systematic analysis for the Global Burden of Disease Study 2021. Lancet. 2024 May 18;403(10440):2100-2132. doi: 10.1016/S0140-6736(24)00367-2. Epub 2024 Apr 3. Erratum in: Lancet. 2024 May 18;403(10440):1988. doi: 10.1016/S0140-6736(24)00824-9. PMID: 38582094; PMCID: PMC11126520.

3. Foreman KJ, Lozano R, Lopez AD, Murray CJ. Modeling causes of death: an integrated approach using CODEm. Popul Health Metr. 2012 Jan 6;10:1. doi: 10.1186/1478-7954-10-1. PMID: 22226226; PMCID: PMC3315398.
