# Supplementary material for: The global, regional, and national burden of urolithiasis in 204 countries and territories, 2000–2021: a systematic analysis for the Global Burden of Disease Study 2021
Source: eClinicalMedicine. 2024 Nov 21;78:102924. doi: 10.1016/j.eclinm.2024.102924 (PMC11618031; doi:10.1016/j.eclinm.2024.102924)
Supplement: Urolithiasis PubMed Table [file mmc3.docx]

**GBD 2021 Urolithiasis Collaborators**

| **Given Names** | **Last Name** |
| --- | --- |
| Atalel Fentahun | Awedew |
| Hannah | Han |
| Bétyna N | Berice |
| Maxwell | Dodge |
| Rachel D | Schneider |
| Mohsen | Abbasi-Kangevari |
| Ziyad | Al-Aly |
| Omar | Almidani |
| Saba | Alvand |
| Jalal | Arabloo |
| Aleksandr Y | Aravkin |
| Tegegn Mulatu | Ayana |
| Nikha | Bhardwaj |
| Pankaj | Bhardwaj |
| Sonu | Bhaskar |
| Boris | Bikbov |
| Florentino Luciano | Caetano dos Santos |
| Jaykaran | Charan |
| Natalia | Cruz-Martins |
| Omid | Dadras |
| Xiaochen | Dai |
| Lankamo Ena | Digesa |
| Muhammed | Elhadi |
| Mohamed A | Elmonem |
| Christopher Imokhuede | Esezobor |
| Ali | Fatehizadeh |
| Teferi Gebru | Gebremeskel |
| Motuma Erena | Getachew |
| Seyyed-Hadi | Ghamari |
| Simon I | Hay |
| Irena M | Ilic |
| Milena D | Ilic |
| Umesh | Jayarajah |
| Seyed Behzad | Jazayeri |
| Min Seo | Kim |
| Sang-woong | Lee |
| Shaun Wen Huey | Lee |
| Stephen S | Lim |
| Mansour Adam | Mahmoud |
| Ahmad Azam | Malik |
| Alexios-Fotios A | Mentis |
| Tomislav | Mestrovic |
| Irmina Maria | Michalek |
| Gedefaye Nibret | Mihrtie |
| Erkin M | Mirrakhimov |
| Ali H | Mokdad |
| Mohammad Ali | Moni |
| Maryam | Moradi |
| Christopher J L | Murray |
| Alberto | Ortiz |
| Shrikant | Pawar |
| Norberto | Perico |
| Mohammad-Mahdi | Rashidi |
| Reza | Rawassizadeh |
| Giuseppe | Remuzzi |
| Austin E | Schumacher |
| Jasvinder A | Singh |
| Valentin Yurievich | Skryabin |
| Anna Aleksandrovna | Skryabina |
| Ker-Kan | Tan |
| Musliu Adetola | Tolani |
| Sahel | Valadan Tahbaz |
| Rohollah | Valizadeh |
| Bay | Vo |
| Asrat Arja | Wolde |
| Seyed Hossein | Yahyazadeh Jabbari |
| Fereshteh | Yazdanpanah |
| Arzu | Yiğit |
| Vahit | Yiğit |
| Mazyar | Zahir |
| Michael | Zastrozhin |
| Zhi-Jiang | Zhang |
| Alimuddin | Zumla |
| Awoke | Misganaw |
| M Ashworth | Dirac |
